# Risk Factors for Invasive *Candida* Infection in Critically Ill Patients: A Systematic Review and Meta-analysis

**DOI:** 10.1016/j.chest.2021.08.081

**Published:** 2021-10-18

**Authors:** Daniel O. Thomas-Rüddel, Peter Schlattmann, Mathias Pletz, Oliver Kurzai, Frank Bloos

**Affiliations:** a Center for Sepsis Control & Care, Jena University Hospital, Jena, Germany; b Department of Anesthesiology and Intensive Care Medicine, Jena University Hospital, Jena, Germany; c Institut für Medizinische Statistik, Informatik und Datenwissenschaften, Jena University Hospital, Jena, Germany; d Institute for Infectious Diseases and Infection Control, Jena University Hospital, Jena, Germany; e National Reference Center for Invasive Fungal Infections NRZMyk, Leibniz Institute for Natural Product Research and Infection Biology – Hans-Knoell-Institute, Jena, Germany; f University of Wuerzburg, Institute for Hygiene and Microbiology, Wuerzburg, Germany

**Keywords:** *Candida*, candidiasis, invasive/epidemiology, candidiasis, invasive, critical care, hospital infections, risk factors, ICI, invasive *Candida* infection

## Abstract

**Background:**

Current guidelines recommend empirical antifungal therapy in patients with sepsis with high risk of invasive *Candida* infection. However, many different risk factors have been derived from multiple studies. These risk factors lack specificity, and broad application would render most ICU patients eligible for empirical antifungal therapy.

**Research Question:**

What risk factors for invasive *Candida* infection can be identified by a systematic review and meta-analysis?

**Study Design and Methods:**

We searched PubMed, Web of Science, ScienceDirect, Biomed Central, and Cochrane and extracted the raw and adjusted OR for each risk factor associated with invasive *Candida* infection. We calculated pooled ORs for risk factors present in more than one study.

**Results:**

We included 34 studies in our meta-analysis resulting in the assessment of 29 possible risk factors. Risk factors for invasive *Candida* infection included demographic factors, comorbid conditions, and medical interventions. Although demographic factors do not play a role for the development of invasive *Candida* infection, comorbid conditions (eg, HIV, *Candida* colonization) and medical interventions have a significant impact. The risk factors associated with the highest risk for invasive *Candida* infection were broad-spectrum antibiotics (OR, 5.6; 95% CI, 3.6-8.8), blood transfusion (OR, 4.9; 95% CI, 1.5-16.3), *Candida* colonization (OR, 4.7; 95% CI, 1.6-14.3), central venous catheter (OR, 4.7; 95% CI, 2.7-8.1), and total parenteral nutrition (OR, 4.6; 95% CI, 3.3-6.3). However, dependence between the various risk factors is probably high.

**Interpretation:**

Our systematic review and meta-analysis identified patient- and treatment-related factors that were associated with the risk for the development of invasive *Candida* infection in the ICU. Most of the factors identified were either related to medical interventions during intensive care or to comorbid conditions.

## Graphical Abstract

**Figure:**
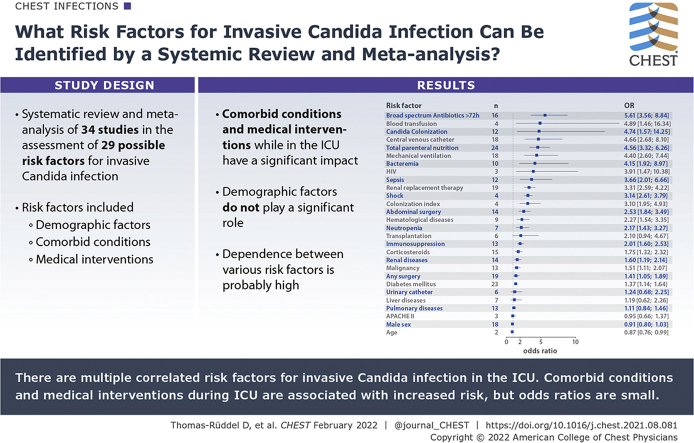

**Take-home Points**

**Study Question:** What risk factors for invasive *Candida* infection can be identified by a systematic review and meta-analysis?

**Results:** We identified 29 risk factors from 34 studies mostly related to medical interventions during intensive care or to comorbid conditions, but most ORs were small.

**Interpretation:** There are multiple correlated risk factors for invasive *Candida* infection in the intensive care setting.

The burden of invasive fungal infections on ICUs is increasing,^1^ and *Candida* species cause approximately 80% of those infections.^2^ Presence of invasive *Candida* infection (ICI) is associated with a high risk of death with an attributable mortality of 49%,^3^ but may increase up to 98% in patients with septic shock with delayed antifungal therapy.^4^ However, early identification of ICI is difficult. Therefore, current guidelines recommend empirical antifungal therapy in patients with sepsis with high risk of ICI.^5^^,^^6^ However, many conditions are known as possible risk factors for ICI.^7^, ^8^, ^9^ These risk factors lack specificity and broad application would render almost every ICU patient eligible for empirical antifungal therapy. The risk factors are mostly derived from retrospective studies reflecting a wide variety of study populations and very different investigated conditions. A systematic review found 13 publications published before 2009 addressing this issue, but heterogeneity of the selected studies was too large to allow a meta-analysis.^10^ Since then, multiple additional studies on this topic have been published. The goal of this meta-analysis is to systematically review the literature on potential risk factors for the development of ICIs in critically ill adult patients and to calculate common ORs for identification of the most important risk factors.

## Methods

The reporting of this study follows the recommendations for Meta-analysis Of Observational Studies in Epidemiology,^11^ and the reporting checklist is provided in e-Table 1. The study protocol was stored locally but not registered prospectively.

### Study Identification

An internet search of relevant publications was performed on five databases (PubMed, Web of Science, ScienceDirect, Biomed Central, and Cochrane) on June 23, 2014. A search algorithm was applied which has been modified from a previous systematic review.^10^ The algorithm contained the following three main criteria: fungal disease, patient population, and risk factors. Each of the three criteria consists of several key words where at least one of the key words in each of the main criteria had to match. The full algorithm is provided in e-Table 2. The PubMed search was updated regularly until December 5, 2018. We reviewed personal files and reference lists of review articles and of articles fulfilling our inclusion criteria for additional relevant publications.

### Eligibility Criteria

We included cohort and case-control studies on adult patients (≥ 18 years of age) admitted to an ICU, which assessed risk factors for the occurrence of ICI either retrospectively or which prospectively followed patients for the development of ICI. The study-specific definition of ICI could be either bloodstream infection (candidemia) or using the European Organization of the Research and Treatment of Cancer/Mycoses Study Group criteria^12^ or using study-specific similar criteria. Studies including the growth of *Candida* species from urine or tracheal aspirates in their case definition were excluded because this mainly reflects colonization and not invasive infection. Control groups had to come from the same ICU population as the cases. Abstracts were included if they contained analyzable results, and no full paper with those data was available. Only publications in English, German, or French were included. If two or more publications were based on the same patient cohort, only one publication was included in the analyses, but all papers were used to extract all available information.

### Study Selection and Data Extraction

Initially, all duplicates from the searches were removed. All titles and in a second step all remaining abstracts were screened by one author (D. T.-R.) for possible eligibility. The full texts of all potentially eligible publications were then rescreened by two authors (F. B. and D. T.-R.); conflicting opinions about eligibility could be resolved in all cases by discussion. Potentially eligible publications identified by the updated PubMed search, personal files, or reference lists were screened the same way.

All reported risk factors and their definition were extracted from the selected publications and thematically grouped by one author (D. T.-R.). Two authors (F. B. and D. T.-R.) decided to collapse identical or nearly identical risk factors into one category and to omit risk factors only assessed by single publications and without reasonable association with ICI from further data extraction. Zero counts in a two-by-two table were replaced by 0.5 to avoid infinite ORs (Haldane-Anscombe-correction). Univariate and, if reported, multivariable ORs and 95% CIs of the applicable risk factors for developing ICI were independently extracted by two authors (F. B. and D. T.-R.) onto standardized data extraction sheets. ORs and CIs were independently calculated by both authors if only frequency data were reported. If a publication reported several cohorts, only data from the cohorts fulfilling inclusion criteria were extracted. Discrepancies were resolved by repeat extraction by both authors, discussion between the data extractors, and if still unresolved discussion with a third author (M. P. and O. K.). The corresponding author was contacted for all articles where data of interest were found to be missing.

### Risk of Bias Assessment

Two authors (F. B. and D. T.-R.) independently assessed the risk of bias for each included study by adapting the Scottish Intercollegiate Guidelines Network quality checklists for cohort studies and case-control studies. The checklists provided measures for assessing internal validity (selection of subjects, assessment of exposure, confounding, and statistical analysis) and overall study quality. Because of the specifics of epidemiologic research in the ICU setting, not all items were applicable, and the checklists were accordingly modified. The modification of the Scottish Intercollegiate Guidelines Network checklist resulted in seven checklist items each for the case-control studies and the cohort studies (e-Table 3). One point was given for each checklist item fulfilled. No points were given if a checklist item was not fulfilled, not applicable, or sufficient information for assessment was not available. In addition, risk of bias was assessed in both study types on a scale of 0 to 2, resulting in a maximum attainable score of 9 in both study types. Discrepancies were resolved by discussion between the data extractors and if still unresolved discussion with a third author (M. P. and O. K.). Both study types were deemed high quality when the score was at 9 points, acceptable quality when the score was 6 to 8 points, and low quality when the score was ≤ 5 points.

### Data Synthesis

Pooled-adjusted univariate and multivariable ORs for each risk factor were calculated using a general inverse variance method with a random effects model. Heterogeneity between studies was investigated using *I*^2^ and the heterogeneity variance τ^2^. Calculations were performed with R (R Foundation for Statistical Computing) and the metagen-function from the meta-package.^13^ SE of the OR was calculated from the OR, the upper limit of the 95% CI, and the probability density of the 97.5% percentile from the normal distribution. Risk of publication bias was assessed by visual inspection of funnel plots for all risk factors reported in at least seven studies and was tested by Egger regression test if at least 10 studies reported a risk factor. In addition, we performed a cumulative meta-analysis for all risk factors with studies ranked starting from the lowest SE for all risk factors. Additional details are provided in e-Figures 1-4.

## Results

### Study Identification

**Figure 1:**
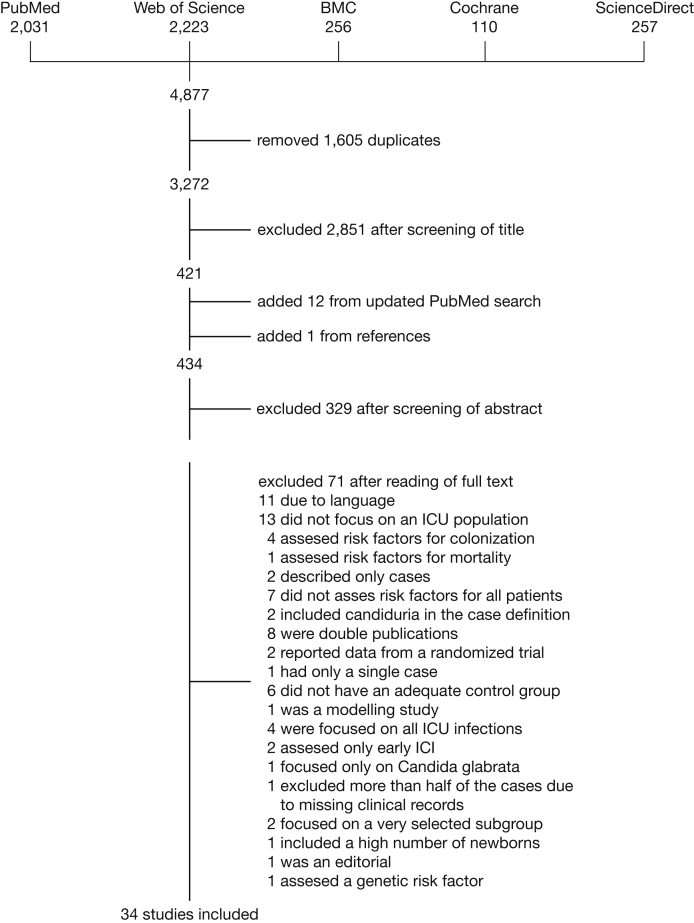
Article flow through different stages of the review. BMC = Biomed Central.

Through the literature search in MEDLINE and Embase, we identified 4,877 references. Duplicate references were identified and removed (n = 1,605), resulting in 3,272 articles. After screening of titles and abstracts and search updates, 104 articles were selected for full-text review, resulting in 34 studies included in this meta-analysis (Fig 1).

### Study Characteristics

A list of the 34 included studies with their characteristics is presented in Table 1.^14^, ^15^, ^16^, ^17^, ^18^, ^19^, ^20^, ^21^, ^22^, ^23^, ^24^, ^25^, ^26^, ^27^, ^28^, ^29^, ^30^, ^31^, ^32^, ^33^, ^34^, ^35^, ^36^, ^37^, ^38^, ^39^, ^40^, ^41^, ^42^, ^43^, ^44^, ^45^, ^46^, ^47^, ^48^, ^49^, ^50^, ^51^ Of these studies,^14^, ^15^, ^16^, ^17^, ^18^, ^19^, ^20^, ^21^, ^22^, ^23^, ^24^, ^25^, ^26^, ^27^, ^28^, ^29^, ^30^, ^31^, ^32^, ^33^, ^34^, ^35^, ^36^, ^37^, ^38^, ^39^, ^40^, ^41^, ^42^, ^43^, ^44^, ^45^, ^46^, ^47^ 12 were prospective cohort studies in design. The remaining 22 studies were either retrospective cohort studies (n = 11) or case-control studies (n = 11). About one-half of the studies (n = 19, 55.9%) recruited patients during the years 2000 and 2010, six studies started recruitment between 1993 and 1999, and nine studies finished recruitment between 2011 and 2015. Median study duration was 2 years (interquartile range, 1-5 years). Most of the studies were monocenter studies (n = 27, 79.4%), whereas only five of the prospective and two of the retrospective cohorts were multicentric in design. In total, cohort studies encompassed 962 cases with ICI out of 86,603 patients and case-control studies encompassed 690 cases and 2,188 patients without ICI. The largest study was the Fungal Infection Risk Evaluation (FIRE) study by the National Institute of Health Research with 16,405 patients observed in 96 UK adult general critical care units.^25^ Table 1 presents the characteristics for each study. Five studies (14.7%) were judged to be of high quality, 18 (52.9%) of acceptable quality, and 11 (32.4%) of low quality (e-Figs 5, 6; e-Table 4). Three datasets were only published as congress abstracts. Two datasets were analyzed in several papers,^27^^,^^30^^,^^48^, ^49^, ^50^, ^51^ sometimes with variations in the included patients or outcomes.

**Table 1:** Characteristics of Selected Studies

Study	Inclusion Period	Design	Patients	Control Patients	Cases	Quality Indicator
Adiguzel et al^14^	2006	Retrospective monocenter cohort	163	…	26	4
Agvald-Ohman et al^15^	2004-2005	Prospective monocenter cohort	59	…	10	6
Ahmed et al^16^	2013-2014	Prospective monocenter cohort	198	…	17	7
Arslan et al^17^	Not reported	1:1 case control	…	140	139	6
Blumberg et al^18^	1993-1995	Prospective multicenter (n = 6) cohort	4,276	…	42	9
Burghi et al^19^^,^^a^	2005-2010	Retrospective monocenter cohort	86	…	7	2
Chander et al^20^	2009	Retrospective monocenter cohort	205	…	24	6
Chow et al^21^	1995-2005	1:5 case-control	…	780	146	8
Eneh et al^22^^,^^a^	2007-2009	Retrospective monocenter cohort	260	…	Not reported	0
Hall et al^23^	2003-2011	Retrospective monocenter cohort	101	…	18	5
Han et al^24^	2000-2006	1:3 case-control	…	147	49	7
Harrison et al^25^	2008-2010	Prospective multicenter (n = 96) cohort	16,405	…	85	8
Hermsen et al^26^	2003-2008	1:3 case control	…	264	88	9
Jorda-Marcos et al,^27^ cohort also analyzed in two other publications^48^^,^^49^	1998-1999	Prospective multicenter (n = 70) cohort	1,765	…	63	9
Kautzky et al^28^	2010-2011	Prospective monocenter cohort	65	…	5	5
Kontopoulou et al^29^^,^^a^	2010-2013	Prospective monocenter cohort	588	…	30	1
Lau et al,^30^ cohort seems to overlap with two other publications^50^^,^^51^^,^^b^	2007-2012	Prospective multicenter (n = 7) cohort	6,015	…	73	7
Leleu et al^31^	1995-1997	1:1 case control	49,063	…	149	4
Leon et al^32^	2006-2007	Prospective multicenter (n = 36) cohort	1,107	…	58	6
Liao et al^33^	2008-2011	Retrospective monocenter cohort	1,253	…	89	8
Manolakaki et al^34^	2002-2007	Retrospective monocenter cohort	374	…	23	5
Michalopoulos et al^35^	1997-1999	1:4 case control	…	120	30	9
Ortiz Ruiz et al^36^	2008-2012	1:2 case control	…	162	81	9
Ostrosky-Zeichner et al^38^	2000-2002	Retrospective multicenter (n = 12) cohort	2,890	…	88	7
Ostrosky-Zeichner et al^37^	2005	Retrospective multicenter (n = 6) cohort	597	…	22	5
Papadimitriou-Olivgeris et al^39^	2012-2015	1:7 case control	…	371	53	8
Paphitou et al^40^	2000	Retrospective monocenter cohort	327	…	36	6
Pasero et al^41^	2005-2007	Prospective monocenter cohort	349	…	26	8
Peres-Bota et al^42^	1999-2000	Retrospective monocenter cohort	280	…	31	6
Posteraro et al^43^	2010	Prospective monocenter cohort	95	…	16	6
Pratikaki et al^44^	2004-2006	1:4 case control	…	132	33	6
Presterl et al^45^	2004	Prospective monocenter cohort	82	…	24	6
Tukenmez et al^46^	2011-2013	1:1 case control	…	37	36	4
Vardakas et al^47^	2001-2007	1:1 case control	…	35	35	5

a Data only reported as abstracts.

b Authors were contacted for clarification several times but did not provide any additional information.

### Unadjusted Risk Factors

Twenty-nine risk factors were extracted from the selected publications. The results for all risk factors except ICU length of stay (e-Fig 7) are presented in Figure 2 (e-Fig 8). The risk factors associated with the highest risk for ICI were broad-spectrum antibiotics (OR, 5.6; 95% CI, 3.6-8.8), blood transfusion (OR, 4.9; 95% CI, 1.5-16.3), *Candida* colonization (OR, 4.7; 95% CI, 1.6-14.3), central venous catheter (OR, 4.7; 95% CI, 2.7-8.1), and total parenteral nutrition (OR, 4.6; 95% CI, 3.3-6.3). ICU length of stay, assessed by four studies, was an outlier with an extremely high risk (OR, 17.3; 95% CI, 4.1-73.0). Meta-analyses, differing definitions, funnel plots, and cumulative meta-analyses for individual risk factors are presented in e-Figures 9-85.

**Figure 2:**
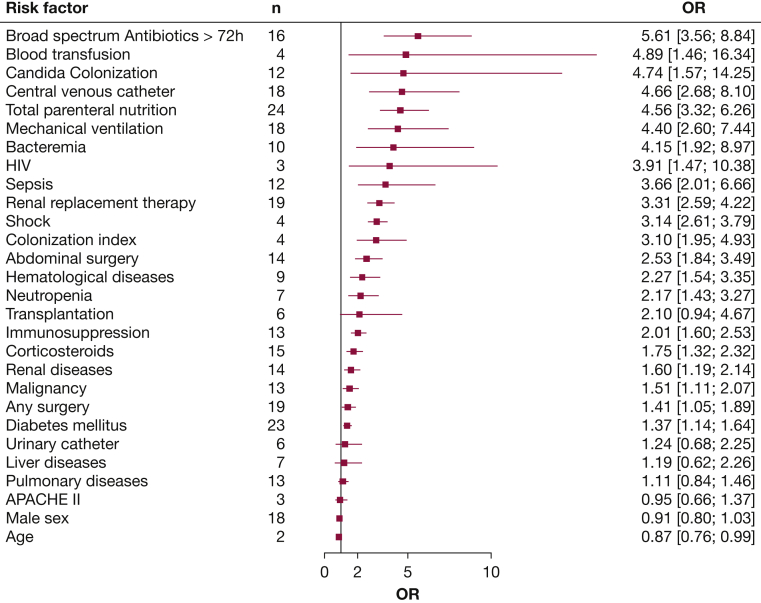
Pooled ORs with 95% CIs for each risk factor. ICU length of stay as an extreme outlier was omitted and is presented in the Supplement (e-Fig 7). Meta-analyses for individual risk factors together with heterogeneity and sequential analysis are presented in the Supplement (e-Figs 9-85). APACHE = Acute Physiology And Chronic Health Evaluation.

Age^17^^,^^28^^,^^34^^,^^39^^,^^42^^,^^47^ (e-Fig 9), APACHE II^17^^,^^39^^,^^42^^,^^47^ (e-Fig 14), ICU length of stay^46^^,^^47^ (e-Fig 16), colonization index^16^ (e-Fig 70), and days of mechanical ventilation and renal replacement therapy^42^ (e-Figs 18, 21) were presented as continuous variables without ORs in several studies. The associations (and lack of associations) of the continuous variables with the risk of ICI were consistent with the findings from the pooled ORs. Heterogeneity measured by *I*^2^ showed a wide range (0%-96%), resulting in significant heterogeneity in 12 of 29 of the observed risk factors. Egger test showed a significant asymmetry of funnel plots only for central venous catheter (e-Fig 63). Cumulative meta-analyses revealed a relevant influence of less accurate studies only for bacteremia (e-Fig 55).

Restricting the analysis to cohort studies or studies of at least acceptable quality did not change results substantially (e-Figs 86-89). Restricting the analysis to the five highest-quality studies resulted in only 18 risk factors reported in median by 3 studies (interquartile range, 2-4 studies) (e-Fig 90), comprising 6,786 patients with 304 cases with ICI. Therefore, risk factor estimates supported by high-quality studies are limited.

### Adjusted Risk Factors

Multivariable analysis results were reported by 17 studies^15^^,^^18^^,^^19^^,^^21^^,^^23^, ^24^, ^25^^,^^27^^,^^29^^,^^33^, ^34^, ^35^, ^36^^,^^39^, ^40^, ^41^^,^^44^ (e-Table 5). All but one study^23^ used stepwise selection in their regression analysis and reported only the final model. Therefore, risk factors not added to the final model were unavailable for meta-analysis. Authors from two studies^21^^,^^24^ were able to provide regression coefficients for all predictors analyzed in their model on request. All others did either not respond, had no access to the modeling data anymore, or had used a forward selection approach.

Pooled ORs for 15 risk factors derived from multivariable analysis in at least two studies are presented in Figure 3 (e-Fig 91). ICU length of stay, which was associated with the highest OR in unadjusted analysis, was associated with no significant risk for ICI in the pooled adjusted analyses. Meta-analyses for individual risk factors including information on risk factors omitted from the final models are presented in e-Figures 92-131. Nine risk factors were only reported in one study and were not included in this meta-analysis (e-Figs 92, 93, 100-102, 105, 108, 121, 125). Funnel plot for parenteral nutrition gave no indication of publication bias (e-Fig 123).

**Figure 3:**
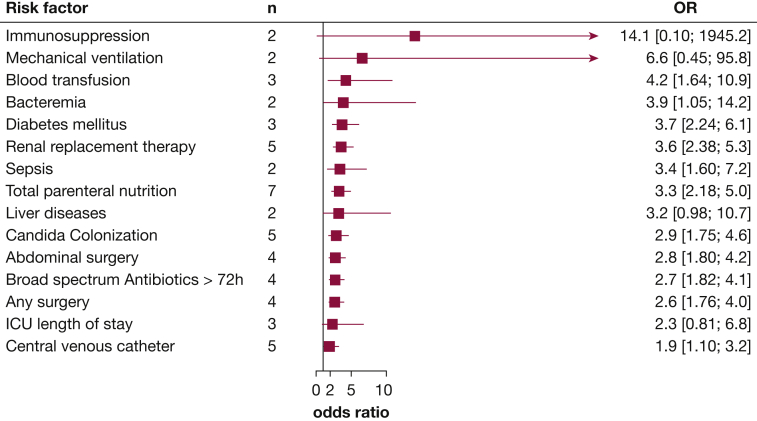
Pooled ORs with 95% CIs for each risk factor from multivariable analyses. Meta-analyses for individual risk factors from multivariable analyses are presented in the Supplement (e-Figs 92-131).

## Discussion

We identified 34 articles that investigated risk factors for ICI in critically ill adults. Apart from confirming the widely recognized risk factors (eg, total parenteral nutrition, *Candida* colonization, [abdominal] surgery, broad-spectrum antibiotics, sepsis), our meta-analysis also identified renal replacement therapy, mechanical ventilation, blood transfusion, and diabetes as important risk factors.

There is biological plausibility for almost all risk factors significantly associated with ICI acquired in the ICU. In health, the immune system is capable in avoiding invasive fungal infections despite presence of these pathogens in primary unsterile body fluids or surfaces. Therefore, immunosuppression, including patients after transplantation or with HIV infections, solid and hematologic malignancies,^52^ neutropenia, and corticosteroid therapy, is an obvious risk factor for developing ICI. Liver cirrhosis,^53^ renal disease,^54^ diabetes,^55^ and blood transfusions^56^ are also associated with immune dysfunction. *Candida albicans* is the most common fungal pathogen that can form biofilms on host-associated abiotic surfaces, including implanted medical devices (eg, central venous, dialysis, or urinary catheters). Therefore, implanted medical devices or *Candida* colonization per se obviously provide a potential source for ICI. Surgery or chemotherapy^52^ might impair the natural body barrier. Especially, translocation from the GI tract may be an important issue because the gut is colonized with *Candida* species.^57^ Broad-spectrum antibiotic therapy increases *Candida* colonization^58^ and might severely affect bacterial and fungal microbiome interaction resulting in a higher pathogenic potential of *Candida* species.^59^ Similar effects have been postulated for oncological chemotherapy.^52^ *Candida* species are capable of multiplying in several parenteral nutrition solutions even in preparations where bacteria cannot grow.^60^^,^^61^ Parenteral nutrition solutions may therefore be contaminated with *C albicans*.^62^ Lipid-containing solutions propagate biofilm formation and germination of *Candida* species.^63^ Similarly, high glucose serum concentrations, also present in diabetes, can increase *Candida* biofilm formation and pathogenicity.^55^ Critically ill patients with renal failure are at risk for ICI because of immune dysfunction^54^ and renal replacement therapy via a vascular catheter instead of a shunt.^64^ Other factors (eg, contamination of the dialysate, colonization of the dialysis machines) have been described for the intermittent dialysis setting^65^^,^^66^ but have not been assessed for the continuous or slow extended daily dialysis on the ICU. Patients with sepsis or septic shock exhibit several risks for developing ICU including antibiotic therapy, invasive therapeutic strategies (implanted medical devices, surgery for source control), gut barrier dysfunction,^67^ and sepsis-induced immunosuppression.^68^

Length of stay in the ICU is the risk factor with the highest OR in the unadjusted analyses. However, there was significant heterogeneity between the studies, likely caused by different cutoffs, and we could not observe an e-table OR in the cumulative meta-analysis. ICU length of stay was extensively analyzed in a recent meta-analyses using wider inclusion criteria than ours but reaching similar results.^69^ This risk factor reflects time at risk and time under observation. A long ICU stay selects patients with severe disease and associated invasive therapies. Therefore, ICU length of stay as a risk factor for ICI is probably highly correlated with other risk factors. Indeed, the effect of ICU length of stay disappears in a study of patients with pancreatitis with 18 ICI cases after adjustment for *Candida* colonization.^23^ In our meta-analysis, ICU length of stay was not associated with the development of ICI in the meta-analysis of multivariable models. However, only three studies included this variable in their model.

Our wide meta-analysis approach has several limitations. Most of the included studies have only a limited number of ICI cases while assessing a large number of risk factors. Such an approach may result in chance findings. On the other hand, the large number of small studies published together with funnel plot inspection makes relevant publication bias unlikely. The assessed patient populations are very heterogeneous. Inclusion and exclusion criteria of the studies, their definitions of ICI, and their matching algorithms for case-control studies differ among the included studies. Some results from special populations might not be generalizable to less selected populations and vice versa. In addition, risk factor definitions or cutoffs differ between studies; most studies do not report definitions at all. Sometimes the wording for a risk factor changes within the same article. Because of these imprecisions in the underlying studies, we might in some cases have calculated common ORs of incongruous risk factors. To help readers with interpretation, we transparently report cutoffs and definitions in the supplement. Many of the assessed risk factors are highly correlated with each other. Therefore, multivariable analyses are of great importance to elucidate the real independent importance of each risk factor. However, only about one-half of the studies performed such an analysis, and the limited number of studies reporting each risk factor limits interpretability. Calculation of reliable common ORs was further hampered by the fact that most studies only reported statistically significant variables in their multivariable analysis. Model building for multivariable analysis including variable selection differed considerably between the studies. Ideally, all studies on such a topic should be consistent in their definitions and report complete multivariable models. A wider use of data repositories for epidemiologic studies in the ICU setting would enable individual patient data meta-analyses. Some studies were excluded because of language restrictions. They were published in six different languages not available in the study team and tended to be smaller studies. Because cumulative meta-analysis showed e-Table ORs for most risk factors, we do not think their inclusion would substantially change our findings. Quality assessment was difficult because all tools found by us were more focused on epidemiologic research in the population and not in a hospitalized cohort and a lot of the checked items were not reported in detail by most studies. Therefore, quality assessment might be less precise than in meta-analyses of randomized trials.

Our central finding is that a large number of risk factors are associated with ICU-acquired ICI, but the risk increase by each factor is relatively small or moderate at best. Future very large epidemiologic studies or individual patient data meta-analyses could only result in more precise estimators for this multitude of risk factors. The success of a risk-driven antifungal therapy^5^^,^^6^ is limited by these low ORs. It would require treating a large number of patients with only moderately elevated risk of ICI. This would result in overtreatment for a large proportion, or it would require a complicated risk assessment incorporating multiple factors to treat only the highest risk patients. Strategies using biomarkers in addition to clinical risk factors^70^, ^71^, ^72^ could be a solution for this dilemma and should be further assessed in large high-quality studies.

## Interpretation

Our systematic review and meta-analysis identified acute and chronic factors that were associated with the risk for the development of ICI in the ICU. Most of the factors identified were either related to medical interventions during intensive care or to comorbid conditions. However, dependence between the various risk factors is probably high. The underlying studies do not sufficiently allow to identify those risk factors independently associated with ICI.

## Supplementary Data

e-Online Data
