# Supplementary material for: Risk Factors for Invasive Candida Infection in Critically Ill Patients: A Systematic Review and Meta-analysis
Source: Chest. 2021 Oct 18;161(2):345–55. doi: 10.1016/j.chest.2021.08.081 (PMC8941622; doi:10.1016/j.chest.2021.08.081)
Supplement: e-Online Data [file mmc1.pdf]

CHEST Online Supplement

Risk Factors for Invasive *Candida* Infection in Critically Ill Patients

A Systematic Review and Meta-analysis

Daniel O. Thomas-Rüddel, MD; Peter Schlattmann, MD; Mathias Pletz, MD; Oliver Kurzai, MD; and Frank Bloos, MD, PhD

CHEST 2022; 161(2):345-355

Online supplements are not copyedited prior to posting and the author(s) take full responsibility for the accuracy of all data.

© 2022 AMERICAN COLLEGE OF CHEST PHYSICIANS. Reproduction of this article is prohibited without written permission from the American College of Chest Physicians. See online for more details. **DOI:** 10.1016/j.chest.2021.08.081

CHEST Online Supplement

Online Supplement to:

"Risk factors for invasive candida infection in critically ill patients: a systematic review and meta-analysis"

Full analysis report and supplementary data

211456

Online supplements are not copyedited prior to posting and the author(s) take full responsibility for the accuracy of all data.

Table of Contents

MOOSE Checklist for Meta-analyses of Observational Studies 2
Background of the analysis 4
Study goal 4
Study protocol 4
Search terms 4
Statistics 5
Risk of bias assessment 6
Publication bias assesment 7
Results Unadjusted data 11
Assessment of bias 11
Complete analysis 14
Unadjusted Meta-analyses of single risk factors 15
Sensitivity Analysis 93
Results adjusted data 96
Assessment of bias 96
Complete analysis 97
Adjusted Meta-analyses of single risk factors 98

MOOSE Checklist for Meta-analyses of Observational Studies

e-Table 1: MOOSE checklist

Item No	Recommendation	Reported on Page No
Reporting of background should include		
1	Problem definition	1
2	Hypothesis statement	1
3	Description of study outcome(s)	1
4	Type of exposure or intervention used	2
5	Type of study designs used	1
6	Study population	1
Reporting of search strategy should include		
7	Qualifications of searchers (eg, librarians and investigators)	S4
8	Search strategy, including time period included in the synthesis and key words	1, S4
9	Effort to include all available studies, including contact with authors	1, 2
10	Databases and registries searched	1
11	Search software used, name and version, including special features used (eg, explosion)	2
12	Use of hand searching (eg, reference lists of obtained articles)	1
13	List of citations located and those excluded, including justification	F1
14	Method of addressing articles published in languages other than English	1
15	Method of handling abstracts and unpublished studies	1
16	Description of any contact with authors	1, T1, 6
Reporting of methods should include		
17	Description of relevance or appropriateness of studies assembled for assessing the hypothesis to be tested	1
18	Rationale for the selection and coding of data (eg, sound clinical principles or convenience)	2
19	Documentation of how data were classified and coded (eg, multiple raters, blinding and interrater reliability)	2
20	Assessment of confounding (eg, comparability of cases and controls in studies where appropriate)	1
21	Assessment of study quality, including blinding of quality assessors, stratification or regression on possible predictors of study results	2
22	Assessment of heterogeneity	2
23	Description of statistical methods (eg, complete description of fixed or random effects models, justification of whether the chosen models account for predictors of study results, dose-response models, or cumulative meta-analysis) in sufficient detail to be replicated	2, S5
24	Provision of appropriate tables and graphics	T1, F1-3, S11-138

Item No	Recommendation	Reported on Page No
Reporting of results should include		
25	Graphic summarizing individual study estimates and overall estimate	F2-3, S11-138
26	Table giving descriptive information for each study included	T1
27	Results of sensitivity testing (eg, subgroup analysis)	6, S93-95
28	Indication of statistical uncertainty of findings	F2-3, S11-138
Reporting of discussion should include		
29	Quantitative assessment of bias (eg, publication bias)	8
30	Justification for exclusion (eg, exclusion of non-English language citations)	8
31	Assessment of quality of included studies	8
Reporting of conclusions should include		
32	Consideration of alternative explanations for observed results	N/A
33	Generalization of the conclusions (ie, appropriate for the data presented and within the domain of the literature review)	8
34	Guidelines for future research	8
35	Disclosure of funding source	9

From: Stroup DF, Berlin JA, Morton SC, et al, for the Meta-analysis Of Observational Studies in Epidemiology (MOOSE) Group. Meta-analysis of Observational Studies in Epidemiology. A Proposal for Reporting. JAMA. 2000;283(15):2008-2012. doi: 10.1001/jama.283.15.2008.

Page numbers anteceded by an S refer to this Supplement. Numbers anteced by an F refer to Figures of the main manuscript. Numbers antecede by an T refer to tables from the main manuscript. N/A means not applicable to our study.

Background of the analysis

Study goal

The goal of this meta-analysis is to systematically review the literature on potential risk factors for the development of invasive candida infections in critically ill patients and to develop common odds ratios for identification of the most important risk factors.

Study protocol

This analysis plan is based on the study protocol from Dec.16, 2014.

Search terms

e-Table 2: search terms

**Fungal disease**	
MeSH terms	mycoses, candida, candidemia
Title/Abstract	fung, candida*, candidemia, candidaemia, candidiasis
**Patient population**	
MeSH terms	critical care, intensive care
Title/Abstract	intensive care, critical care, critical illness, critically ill
**Risk factors**	
MeSH terms	Risk, "models, statistical", regression analysis, logistic models, odds ratio, sensitivity and specificity, survival analysis, operations research", multivariate analysis, decision support techniques, clinical protocols, practice guidelines as topic, patient selection
Title/Abstract	score, risk*, predict*, odds, rule

Modified from Muskett H, Shahin J, Eyres G, Harvey S, Rowan K, Harrison D. Risk factors for invasive fungal disease in critically ill adult patients: a systematic review. Critical care (London, England) 2011; 15: R287.

The search was designed and performed by FB, a experienced clinical researcher. The search was performed on https://pubmed.ncbi.nlm.nih.gov, Web of Science, https://www.sciencedirect.com, https://www.biomedcentral.com and https://www.cochranelibrary.com/advanced-search on 23rd of June 2014. Search results were exported to Endnote (Calirvate Analytics, New York City). The search on Pubmed was saved and regular email alerts for new results were received until the 5th of December 2018.

Statistics

This analysis is based on the data-file **2021-02-16_Datenextraktion_final.xlsx** containing the data extraction from Jan.10, 2020 including the correction of the adjusted data from Jan.20, 2021 (Removal of the study by Adrigüzel) and the addition of the study by *Ortiz Ruiz*. Meta-analysis was done with the R-package meta:

```
library (meta)
```

Schwarzer G. meta: An R package for meta-analysis. R news 2007; 7(3): 40-5.

Odds ratios and 95% confidence intervals were either taken directly from the publication or calculated by DTR and FB if only frequency tables were given in the publication. Zero counts in a two-by-two table were replaced by 0.5 to avoid infinite odds ratios (Haldane-Anscombe-correction).

Standard error of the odds ratio was calculated from the OR and the upper limit of the 95% confidence interval as follows:

```
calcse<-function(or,ul)
  {
  logor<-log(or)
  logul<-log(ul)
  se<-(logul-logor)/qnorm(0.975)
  return(se)
}
```

function written by Schlattmann

Metanalysis was performed with the metagen command by using the following syntax:

```
metagen(log(x[, c(OR, se_OR)]),
             sm="OR")
```

where x is the data.frame containing the ORs taken from the studies.

Problem Study Chow 2008: This study separately reports ORs for non-*albicans* and *albicans* candidemia against the same control group. For this analysis, ORs and confidence intervals are combined by the average of the log odds ratios: or = exp((ln(OR1)+ln(OR2))/2). However, this procedure might underestimate the confidence intervall. (see: **http://www.metafor-project.org/doku.php/analyses:gleser2009**)

Risk of bias assessment

Two authors (FB and DTR) independently assessed the risk of bias for each included study by adapting the Scottish Intercollegiate Guidelines Network (SIGN) quality checklists for cohort studies and case control studies 10. The checklists provided measures for assessing internal validity (selection of subjects, assessment of exposure, confounding, and statistical analysis) and overall study quality. The modification of the SIGN checklist resulted in 9 checklist-items for the case control studies and the cohort studies (eTable 2 in the electronic supplement). One point was given for each checklist-item fulfilled. No points were given if a checklist-item was not fulfilled, not applicable, or sufficient information for assessment was not available. In addition, risk of bias was assessed in both study types on a scale of 0 to 2 resulting in a maximum attainable score of 10 in both study types. Discrepancies were resolved by discussion between the data extractors and if still unresolved discussion with a third author (MP, OK). Both study-types were deemed high quality (HQ) when the score was at least 9 points, acceptable quality (AQ) when the score was 6-8, and low quality (LQ) when the score was less than or equal to 5 points.

e-Table 3: Modified SIGN Criteria

Cohort studies	Case control studies
The two groups being studied are selected from source populations that are comparable in all respects other than the factor under investigation.<br><br>yes/no/can't say/does not apply	The cases and controls are taken from comparable populations.<br><br>yes/no
The likelihood that some eligible subjects might have the outcome at the time of enrolment is assessed and taken into account in the analysis.<br><br>yes/no/can't say/does not apply	The same exclusion criteria are used for both cases and controls.<br><br>yes/no/can't say
The outcomes are clearly defined.<br><br>yes/no/can't say	Cases are clearly defined and differentiated from controls.<br><br>yes/no/can't say
The assessment of outcome is made blind to exposure status. If the study is retrospective this may not be applicable.<br><br>yes/no/can't say/does not apply	It is clearly established that controls are non-cases.<br><br>yes/no/can't say
The method of assessment of exposure is reliable.<br><br>yes/no/can't say	Measures will have been taken to prevent knowledge of primary exposure influencing case ascertainment.<br><br>yes/no/can't say/does not apply
Evidence from other sources is used to demonstrate that the method of outcome assessment is valid and reliable.<br><br>yes/no/can't say/does not apply	Exposure status is measured in a standard, valid and reliable way.<br><br>yes/no/can't say
The main potential confounders are identified and taken into account in the design and analysis.<br><br>yes/no/can't say	
Have confidence intervals been provided?<br><br>yes/no	
How well was the study done to minimise the risk of bias or confounding?<br><br>High quality/acceptable/unacceptable	

Modified from http://www.sign.ac.uk/checklists-and-notes.html (accessed Feb 20, 2018).

Publication bias assesment

Risk factors *male sex* and *parenteral nutrition* are selected as marker variables for publication bias since they have been assessed in most studies. See appendix for the other variables. Funnel plot asymmetry was tested with the linear regression test according to Egger (`metabias` of the meta-package).

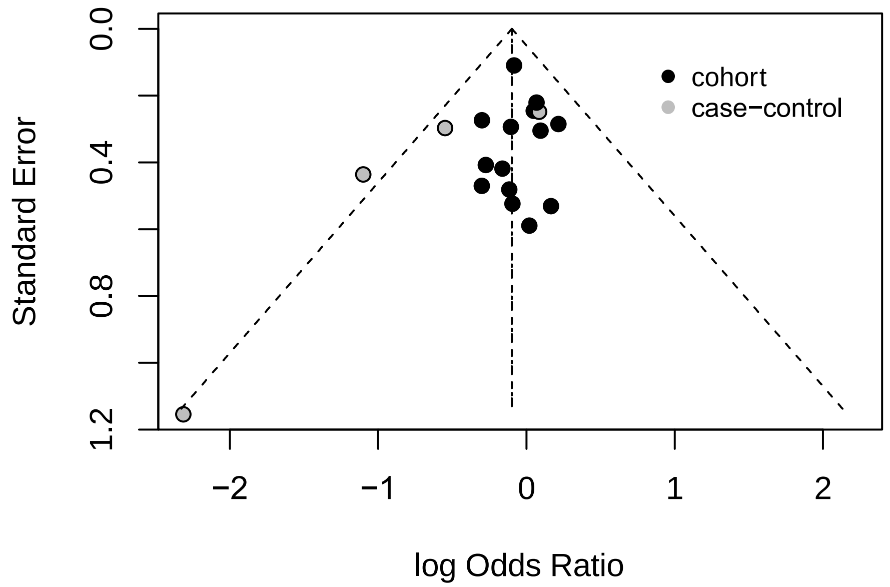

e-Figure 1: Visually, there is no publication bias (funnel plot asymmetry p = 0.145) and no significant heterogeneity for sex (I^2 = 0% [0%, 50%]).

The following analysis is showing a cumulative metaanalyis with the function `metacum(x, pooled = "random", sortvar = .seTE)`. Studies are added step by step ranked by the standard error for the odds ratio of interest. First study added has the lowest standard error.

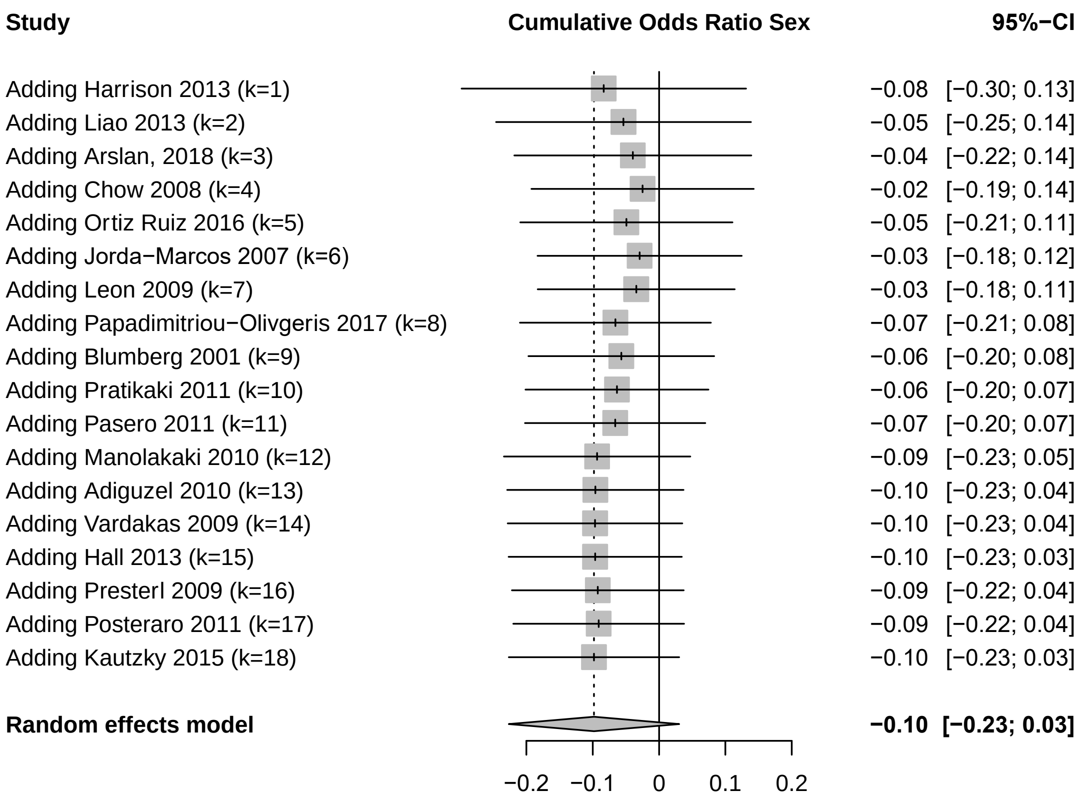

e-Figure 2: The impact on the common odds ratio for sex remains e-Table after 11 studies added. However, changes in the common odds ratios are not really relevant.

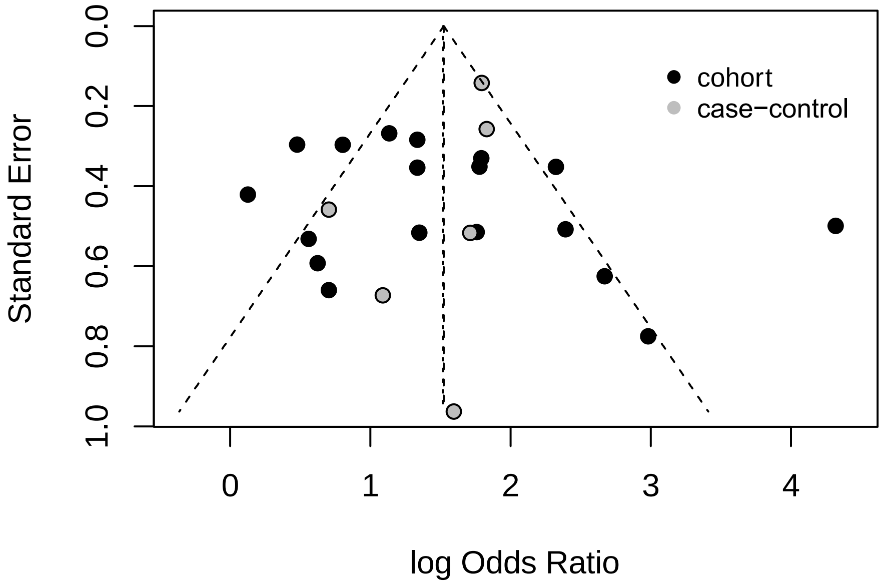

e-Figure 3: Visually, the funnel plot is asymmetric but this is not significant (funnel plot asymmetry p = 0.921) and there is significant heterogeneity for *total parenteral nutrition* (I^2 = 76.1% [64.6%, 83.8%]).

The following analysis is showing a cumulative meta-analyis with the function `metacum(x, pooled = "fixed", sortvar = .seTE)`. Studies are added step by step ranked by the standard error for the odds ratio of interest. First study added has the lowest standard error.

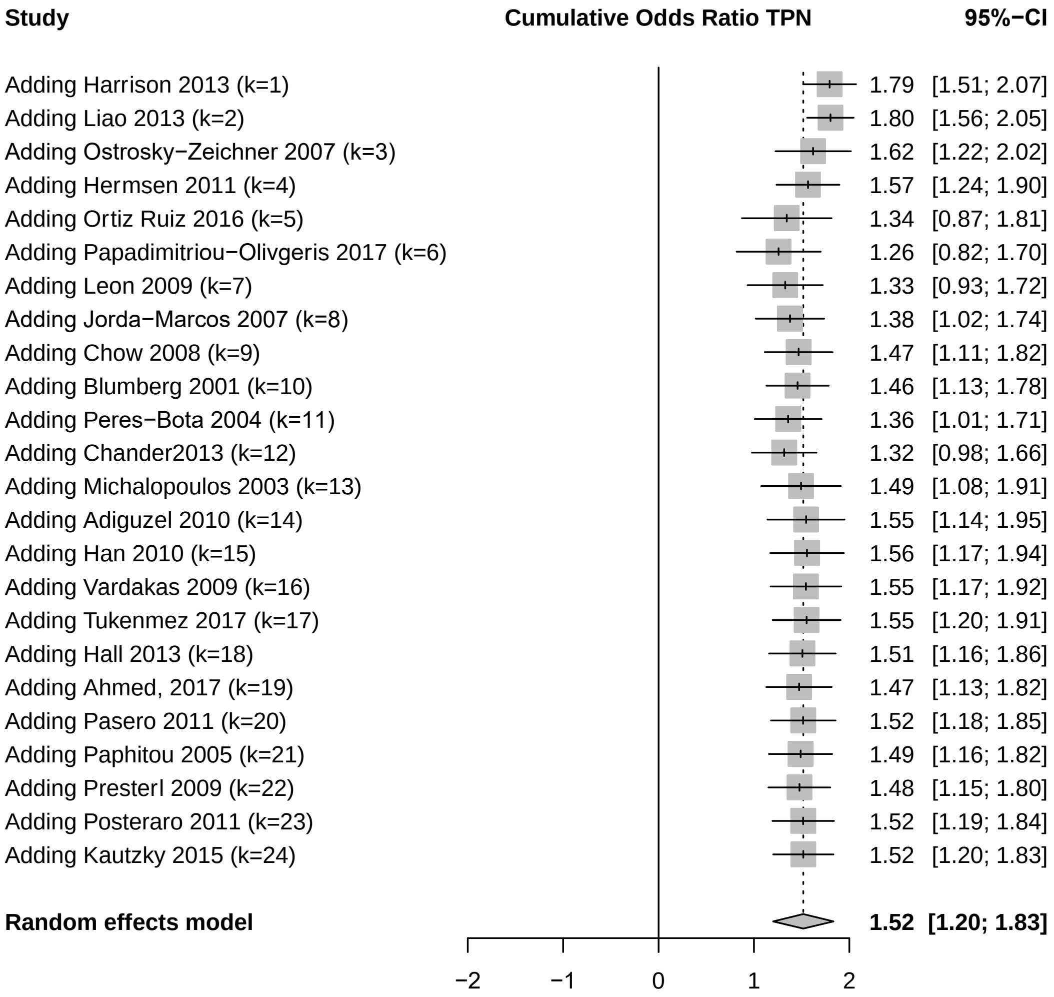

e-Figure 4: The combined odds ratio for *total parenteral nutrition* seems to be accurate despite significant heterogeneity.The combined odds ratio remains e-Table after 6 studies were added to the meta-analysis. Less accurate studies do not have an impact on the final result.

Results Unadjusted data

Assessment of bias

This meta-analysis inludes 34 studies: 24 cohort and 10 case control studies. Overall quality was moderate with a median of 6 where 11 studies were of low, 18 studies of acceptable and 5 studies of high quality.

e-Table 4: Bias assessment for each study

Study	Design	Quality indicator
Adiguzel 2010	cohort	4
Agvald-Ohman 2008	cohort	6
Ahmed, 2017	cohort	7
Arslan, 2018	case control	6
Blumberg 2001	cohort	9
Burghi 2011**	cohort	2
Chander2013	cohort	6
Chow 2008	case control	8
Eneh 2010*	cohort	0
Hall 2013	cohort	5
Han 2010	case control	7
Harrison 2013	cohort	8
Hermsen 2011	case control	9
Jorda-Marcos 2007	cohort	9
Kautzky 2015	cohort	5
Kontopoulou 2014*	cohort	1
Lau 2015	cohort	7
Leleu 2002	cohort	4
Leon 2009	cohort	6
Liao 2013	cohort	8
Manolakaki 2010	cohort	5
Michalopoulos 2003	case control	9
Ortiz Ruiz 2016	case control	9
Ostrosky-Zeichner 2007	cohort	7
Ostrosky-Zeichner 2011	cohort	5
Papadimitriou-Olivgeris 2017	case control	8
Paphitou 2005	cohort	6
Pasero 2011	cohort	8
Peres-Bota 2004	cohort	6
Posteraro 2011	cohort	6
Pratikaki 2011	case control	6
Presterl 2009	cohort	6
Tukenmez 2017	case control	4
Vardakas 2009	case control	5

*: study with abstract only.

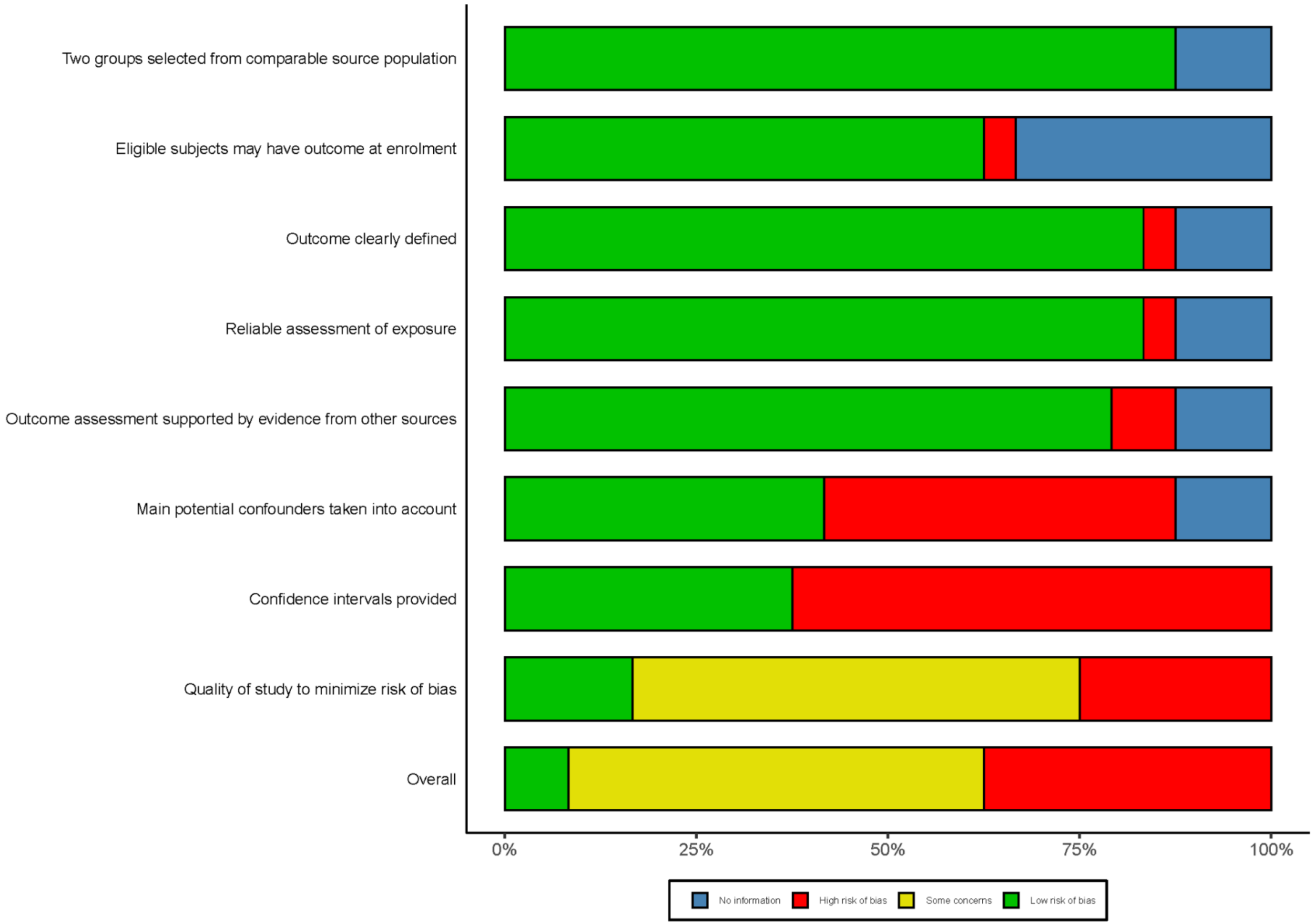

e-Figure 5: Bias Cohort Studies - Items

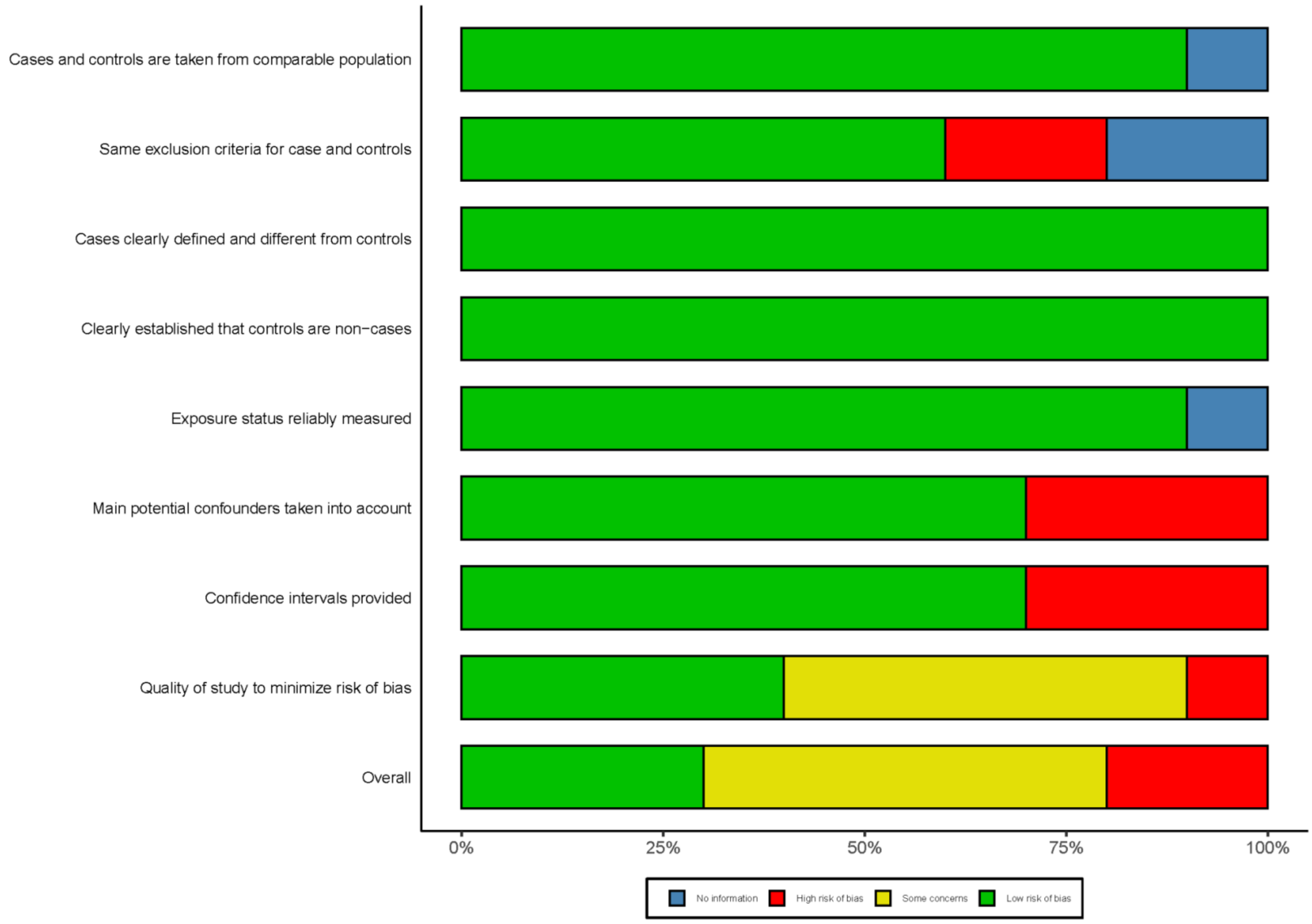

e-Figure 6: Bias Case Control Studies - Items

Complete analysis

The following figure shows the common odds ratios (**random effects model**) of all obtained risk factors for invasive candida infection. Risk factors represented in one study only are excluded. Forest plot for all single risk factors are shown in the appendix.

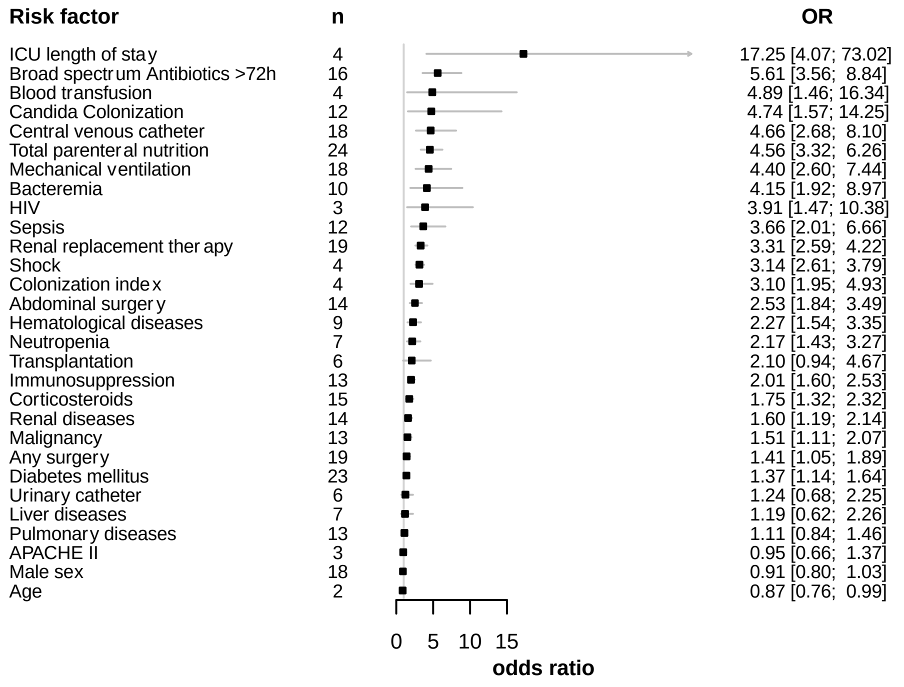

e-Figure 7: n: number of studies assessing this risk factor; or: odds ratio with 95% confidence intervals

Odds ratio for ICU length of stay is a significant outlier. The following figure presents the odds ratios with this parameter exluded.

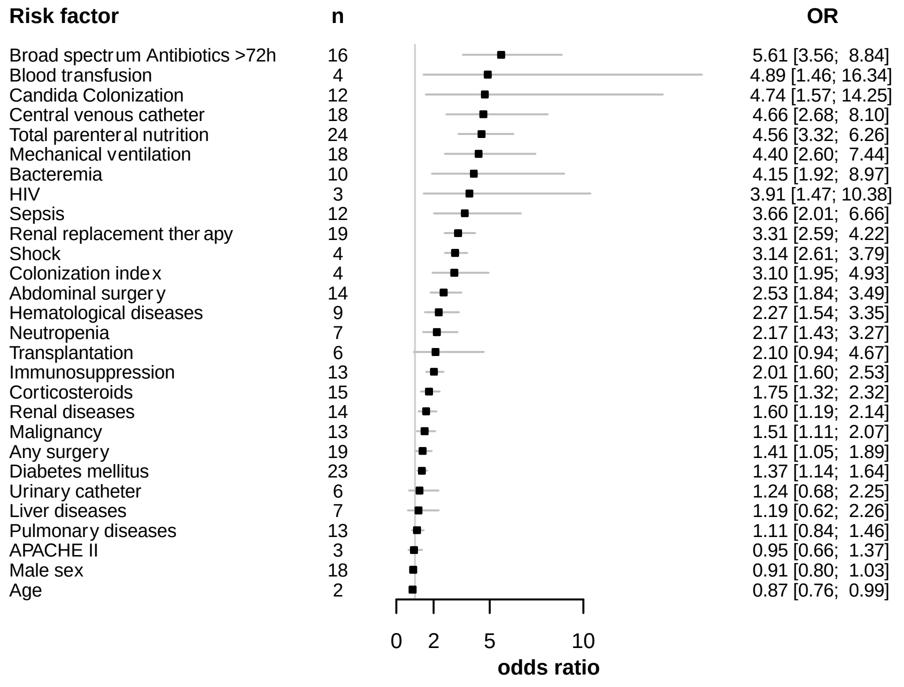

e-Figure 8: n: number of studies assessing this risk factor; or: odds ratio with 95% confidence intervals

Unadjusted Meta-analyses of single risk factors

Cumulative meta-analysis is only done if two studies observed the risk factor.

Funnel plot is presented if at least seven studies reported the risk factor and Egger test for Funnel plot asymmetry was performed if at least ten studies reported the risk factor.

Cutoffs used to dichotomize continuous variables or units of measurement used by individual studies are reported in the footnotes for each risk factor meta-analysis. Definitions of risk factors significantly deviating from the terms or definitions used by the majority of studies are also reported in the footnotes.

Where continuous variables where only reported as summary statistics they are reported in the footnote either as mean±SD or median [IQR Q1-Q3] or median (range Min-Max) with values for ICI first versus (vs.) controls followed by the reported p value or not significant (NS) in parentheses as reported in the individual studies.

When a case control study did match for a certain risk factor, or when a risk factor was inclusion or exclusion criterion of a study no data was extracted but it is reported in the footnote.

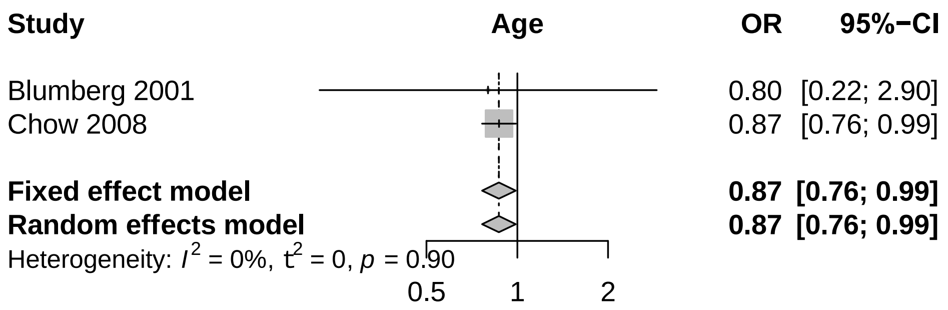

Age ≥ 65 years in Blumberg 2001
Per decade in Chow 2008

65 [55-73] years vs. 64 [53-79] (p=0.5) in Arslan 2018
28.2±9.7 years vs. 52.7±15.7 (p=0.003) in Kautzky 2015
42.7±20.3 years vs. 45.4±19.7 (p=0.5) in Manonalaki 2010
62.0±17.6 years vs. 61.2±16.9 (p=0.8) in Papadimitiou-Olivgeris 2017
64 (24-81) years vs. 58 (18-81) (NS) in Peres-Bota 2004

Age was matched for in Turkmenez 2017, Ortiz Ruiz 2016 & Vardakas 2009

e-Figure 9

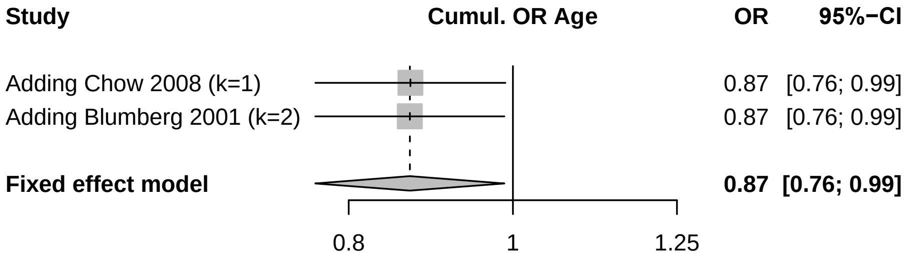
Study
Cumul. OR Age
OR
95%−CI
Adding Chow 2008 (k=1)
0.87 [0.76; 0.99]
Adding Blumberg 2001 (k=2)
0.87 [0.76; 0.99]
Fixed effect model
0.87 [0.76; 0.99]
0.8
1
1.25

e-Figure 10

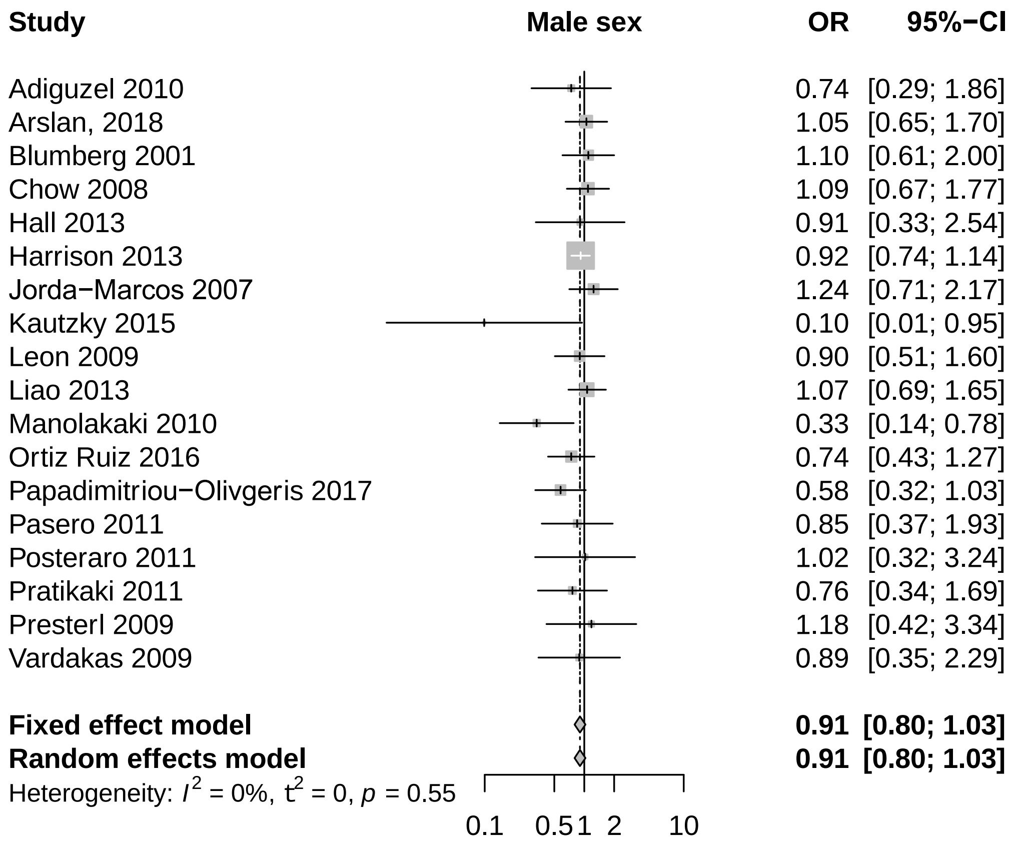

Sex was matched for in Turkenez 2017

e-Figure 11

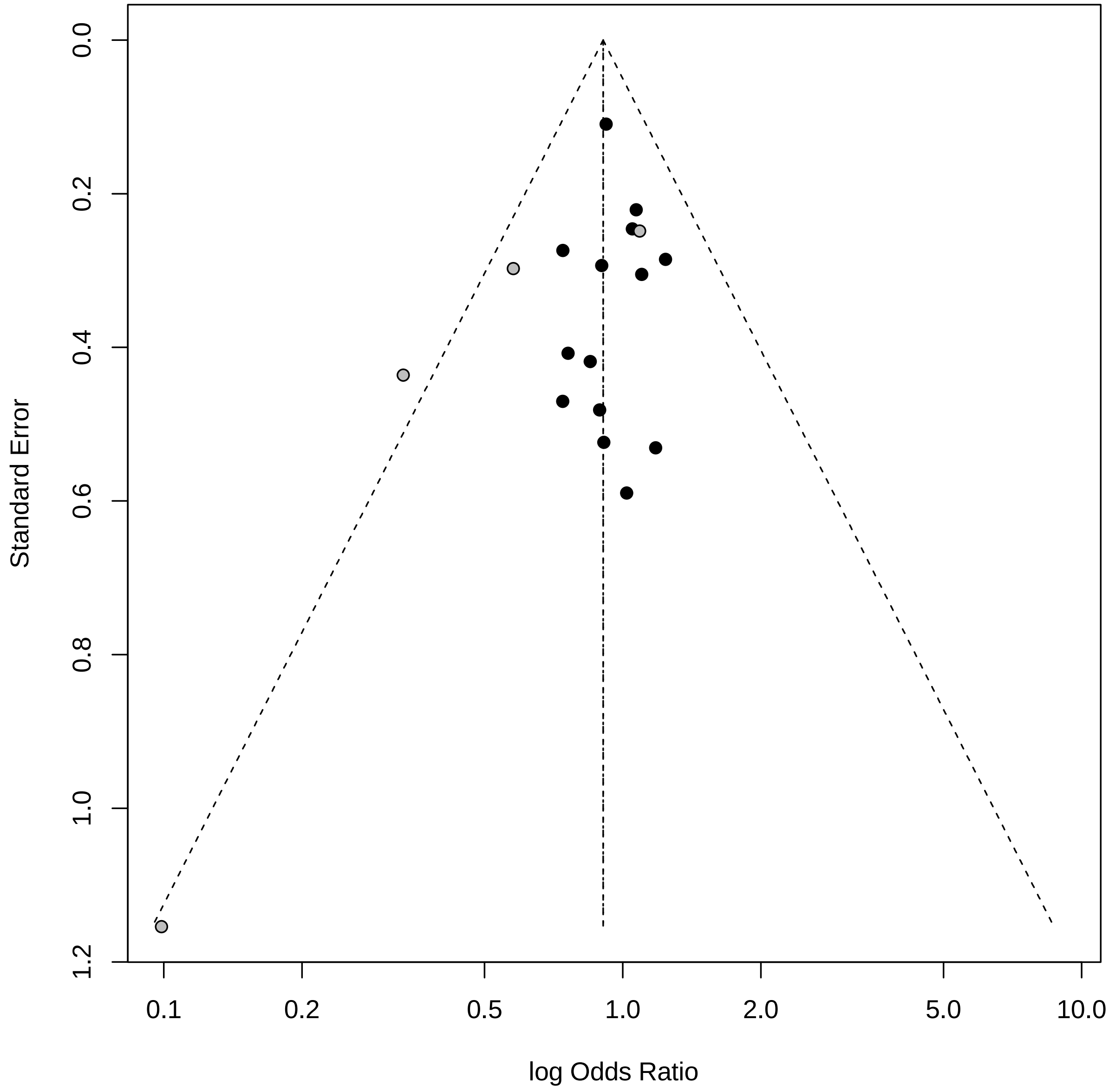

e-Figure 12

```
## [1] "p-value for Funnel-plot asymmetry (Eggers-test):  0.145"
```

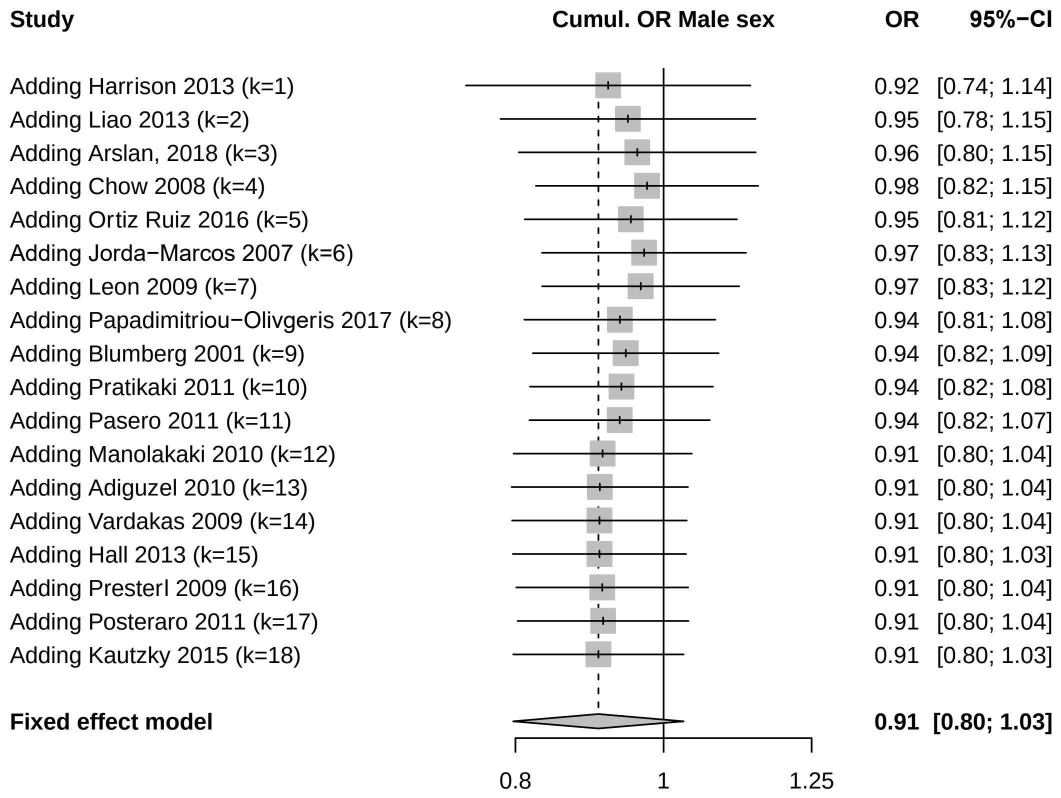
Study
Cumul. OR Male sex
OR
95%−CI
Adding Harrison 2013 (k=1) 0.92 [0.74; 1.14]
Adding Liao 2013 (k=2) 0.95 [0.78; 1.15]
Adding Arslan, 2018 (k=3) 0.96 [0.80; 1.15]
Adding Chow 2008 (k=4) 0.98 [0.82; 1.15]
Adding Ortiz Ruiz 2016 (k=5) 0.95 [0.81; 1.12]
Adding Jorda−Marcos 2007 (k=6) 0.97 [0.83; 1.13]
Adding Leon 2009 (k=7) 0.97 [0.83; 1.12]
Adding Papadimitriou−Olivgeris 2017 (k=8) 0.94 [0.81; 1.08]
Adding Blumberg 2001 (k=9) 0.94 [0.82; 1.09]
Adding Pratikaki 2011 (k=10) 0.94 [0.82; 1.08]
Adding Pasero 2011 (k=11) 0.94 [0.82; 1.07]
Adding Manolakaki 2010 (k=12) 0.91 [0.80; 1.04]
Adding Adiguzel 2010 (k=13) 0.91 [0.80; 1.04]
Adding Vardakas 2009 (k=14) 0.91 [0.80; 1.04]
Adding Hall 2013 (k=15) 0.91 [0.80; 1.03]
Adding Presterl 2009 (k=16) 0.91 [0.80; 1.04]
Adding Posteraro 2011 (k=17) 0.91 [0.80; 1.04]
Adding Kautzky 2015 (k=18) 0.91 [0.80; 1.03]
Fixed effect model 0.91 [0.80; 1.03]
0.8
1
1.25

e-Figure 13

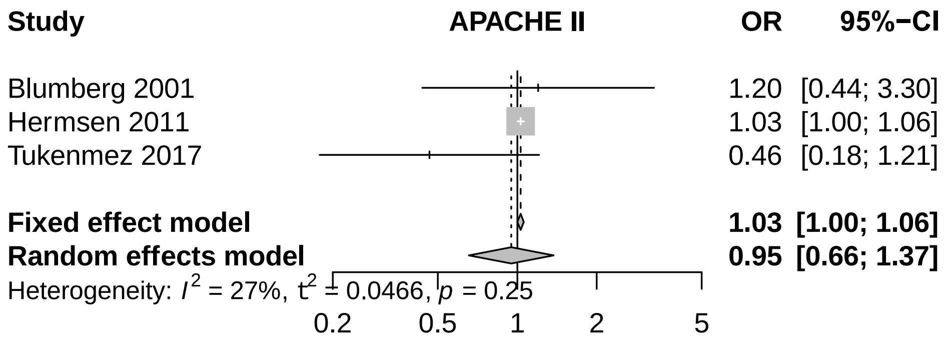

≥ 25 points in Blumberg 2001
Per point increase in Hermsen 2011
> 20 points in Turkenez 2017

16 [11-22]	points vs. 16 [12-21]	(p=0.7)	in Arslan 2018
18 [13-22]	points vs. 17 [12-22]	(p=0.6)	in Ortiz Ruiz 2016
17.4±7.0	points vs. 17.8±7.0	(p=0.7)	in Papadimitriou-Olivgeris 2017
18.4±6.0	points vs. 17.0±7.0	(NS)	in Peres-Bota 2004
20.3±7.9	points vs. 20.4±7.2	(p=0.9)	in Vardakas 2009

e-Figure 14

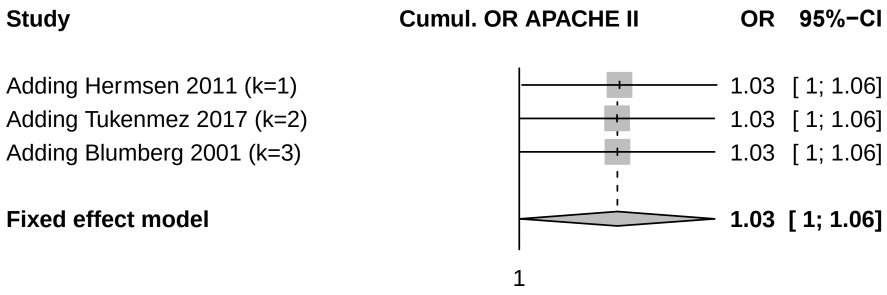
Study
Cumul. OR APACHE II
OR 95%−CI
Adding Hermsen 2011 (k=1)
1.03 [1; 1.06]
Adding Tukenmez 2017 (k=2)
1.03 [1; 1.06]
Adding Blumberg 2001 (k=3)
1.03 [1; 1.06]
Fixed effect model
1.03 [1; 1.06]
1

e-Figure 15

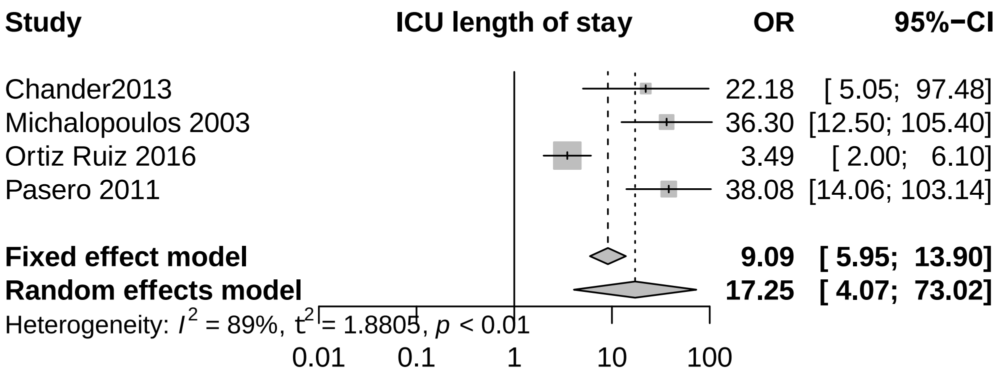

> 15 days in Chander 2013
≥ 9 days in Michalopoulos 2003
> 14 days in Ortiz Ruiz 2016
> 20 days in Pasero 2011

22 [15-33]days vs. 3.0 [3-6] (p<0.01) in Chow 2008 for Candida albicans
25 [14-40]days vs. 3.0 [3-6] (p<0.01) in Chow 2008 for Candida non albicans
17 [9-23] days vs. 7.3 [3-15] (p<0.01) in Hall 2013
27 ± 7.5 days vs. 2.0 ± 1.6 (p<0.01) in Michalopoulos 2003, see above for dichotomized data
22 ± 1.7 days vs. 14 ± 1.4 (p<0.01) in Ortiz Ruiz 2016, see above for dichotomized data
22 [18;30]days vs. 5.5 [2;16] (p<0.01) in Tukenmez 2017

matched for in Vardakas 2009 & Pratikaki 2011

e-Figure 16

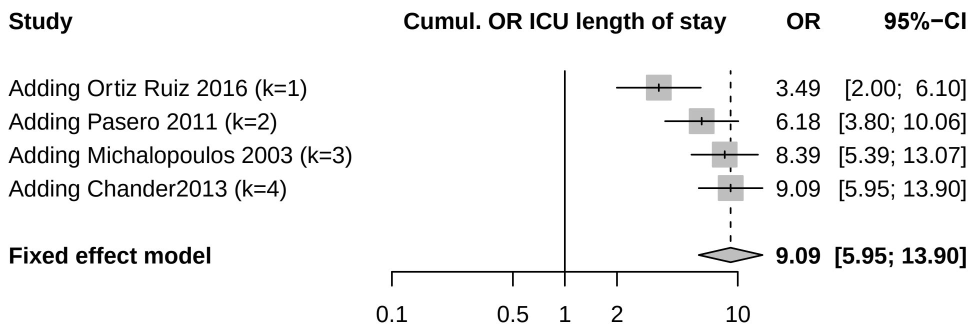
Study
Cumul. OR ICU length of stay
OR
95%−CI
Adding Ortiz Ruiz 2016 (k=1)
3.49 [2.00; 6.10]
Adding Pasero 2011 (k=2)
6.18 [3.80; 10.06]
Adding Michalopoulos 2003 (k=3)
8.39 [5.39; 13.07]
Adding Chander2013 (k=4)
9.09 [5.95; 13.90]
Fixed effect model
9.09 [5.95; 13.90]
0.1
0.5
1
2
10

e-Figure 17

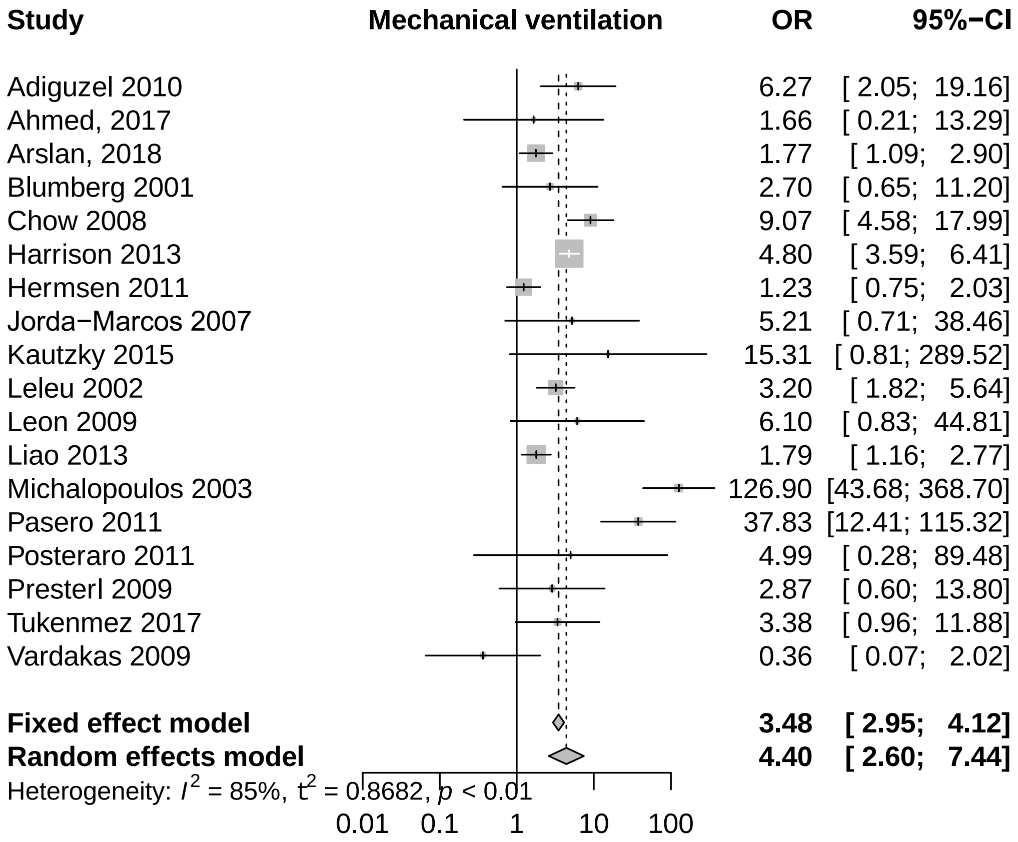

≥ 2 days in Liao 2013
≥ 10 days in Michalopoulos 2003
> 10 days in Pasero 2011

All patients were ventilated in Papadimitriou-Olivgeris 2017

10.9±4.1 days vs. 4.0±4.7 (0.03) in Peres-Bota 2004

e-Figure 18

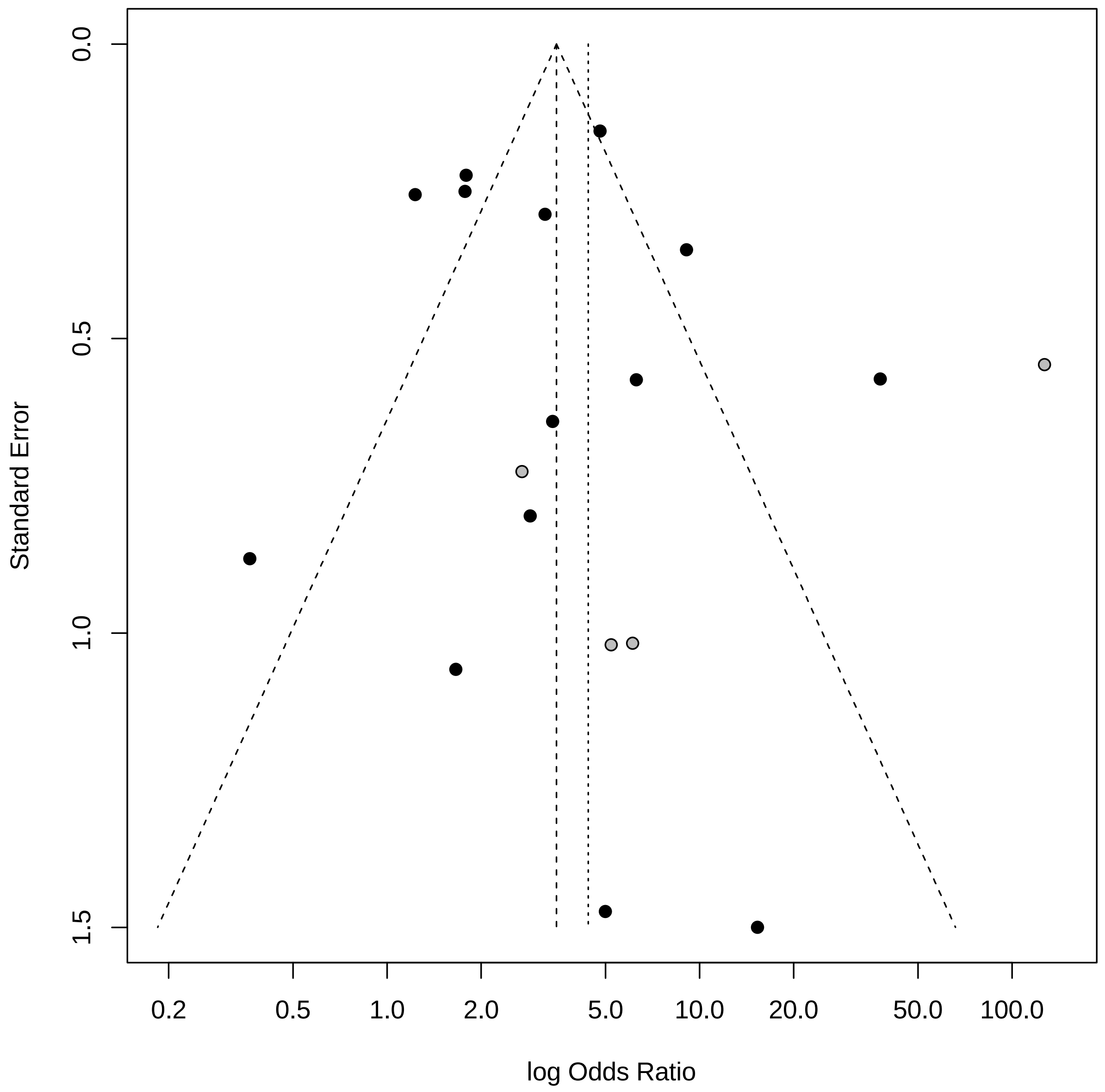

e-Figure 19

```
## [1] "p-value for Funnel-plot asymmetry (Eggers-test):  0.463"
```

Study	OR	95%−CI
Adding Harrison 2013 (k=1)	4.80	[3.59; 6.41]
Adding Liao 2013 (k=2)	3.55	[2.79; 4.52]
Adding Arslan, 2018 (k=3)	3.10	[2.50; 3.85]
Adding Hermsen 2011 (k=4)	2.68	[2.20; 3.27]
Adding Leleu 2002 (k=5)	2.74	[2.27; 3.30]
Adding Chow 2008 (k=6)	2.97	[2.48; 3.56]
Adding Michalopoulos 2003 (k=7)	3.30	[2.76; 3.95]
Adding Pasero 2011 (k=8)	3.51	[2.94; 4.19]
Adding Adiguzel 2010 (k=9)	3.56	[2.99; 4.24]
Adding Tukenmez 2017 (k=10)	3.56	[2.99; 4.23]
Adding Blumberg 2001 (k=11)	3.54	[2.99; 4.20]
Adding Presterl 2009 (k=12)	3.53	[2.98; 4.19]
Adding Vardakas 2009 (k=13)	3.46	[2.92; 4.09]
Adding Leon 2009 (k=14)	3.47	[2.93; 4.11]
Adding Jorda−Marcos 2007 (k=15)	3.48	[2.94; 4.12]
Adding Ahmed, 2017 (k=16)	3.46	[2.93; 4.09]
Adding Posteraro 2011 (k=17)	3.47	[2.93; 4.10]
Adding Kautzky 2015 (k=18)	3.48	[2.95; 4.12]
**Fixed effect model**	**3.48**	**[2.95; 4.12]**

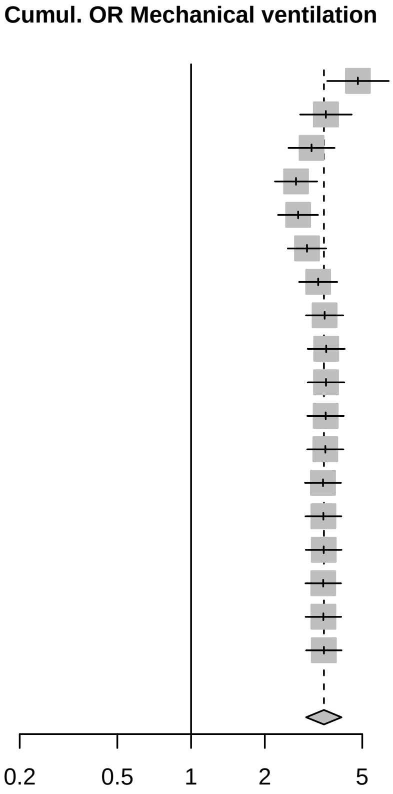

e-Figure 20

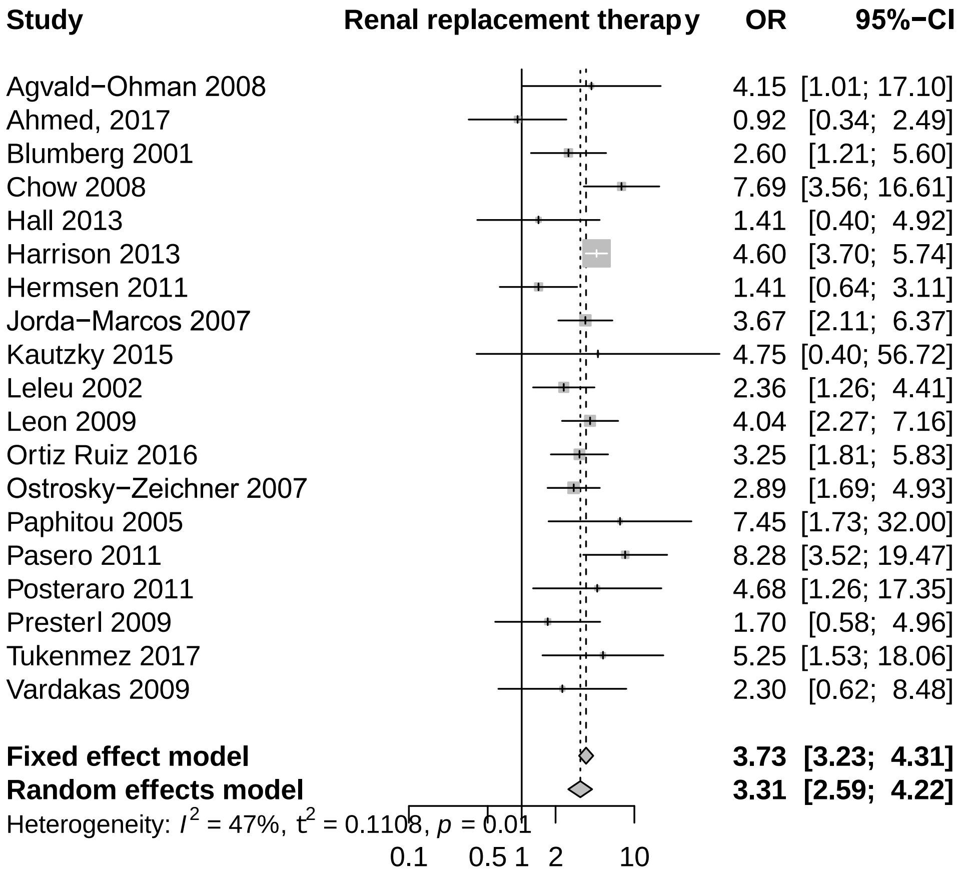

Any RRT up to day 3 in Papithou 2005
Any RRT up day 1-3 in Ostrosky-Zeichner 2007

1.7±2.2 days vs. 0.6±2.3 (p=0.03) in Peres-Bota 2004

e-Figure 21

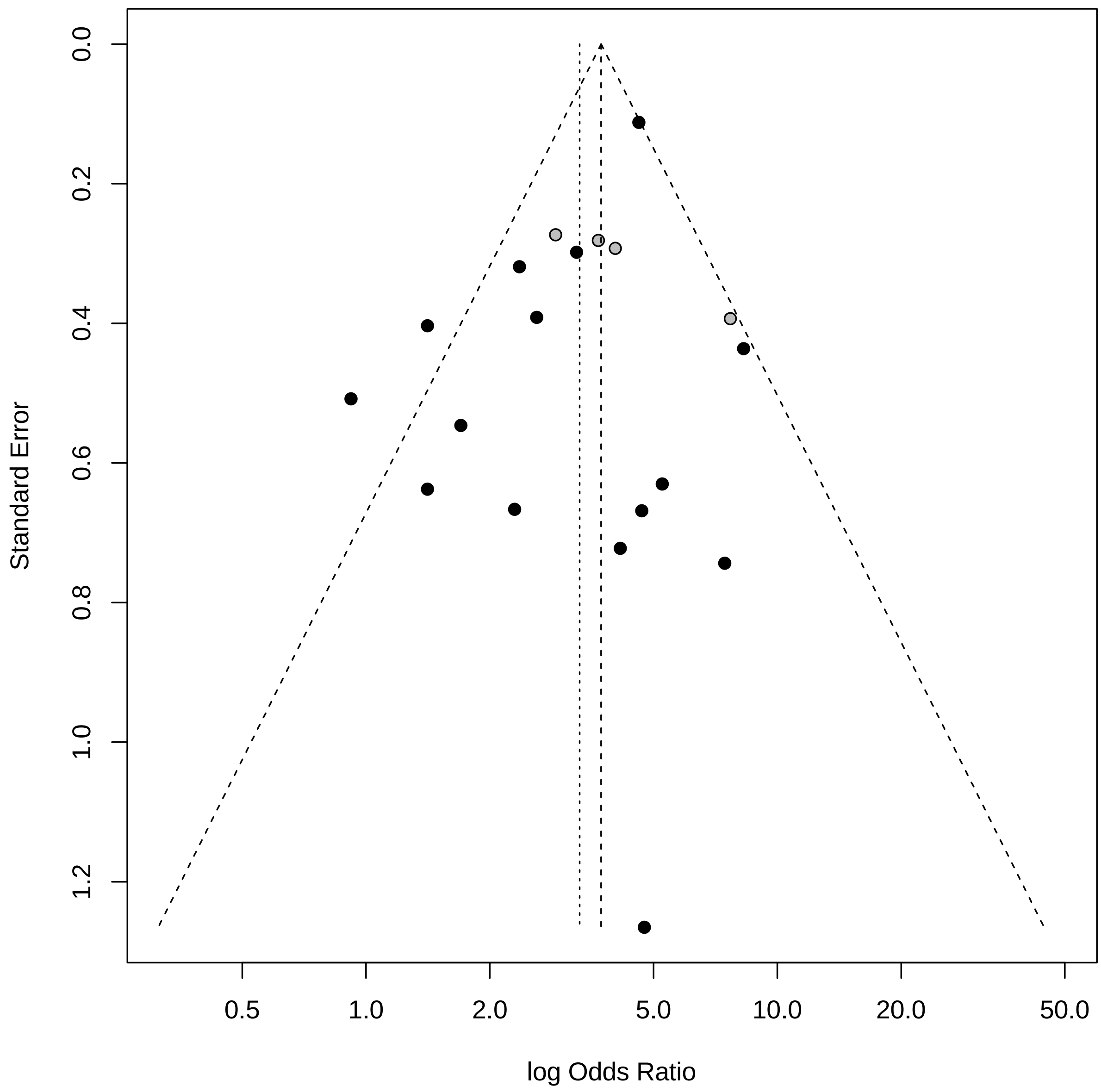

e-Figure 22

```
## [1] "p-value for Funnel-plot asymmetry (Eggers-test):  0.159"
```

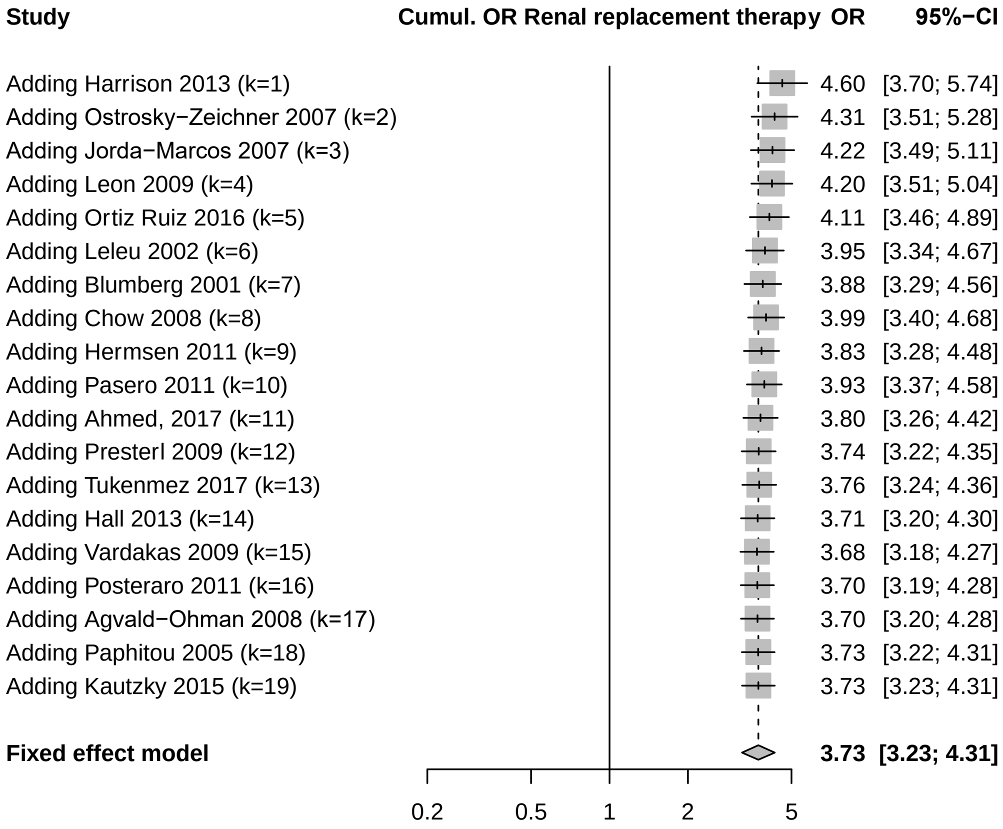
Study
Cumul. OR Renal replacement therapy
OR
95%−CI
Adding Harrison 2013 (k=1) 4.60 [3.70; 5.74]
Adding Ostrosky−Zeichner 2007 (k=2) 4.31 [3.51; 5.28]
Adding Jorda−Marcos 2007 (k=3) 4.22 [3.49; 5.11]
Adding Leon 2009 (k=4) 4.20 [3.51; 5.04]
Adding Ortiz Ruiz 2016 (k=5) 4.11 [3.46; 4.89]
Adding Leleu 2002 (k=6) 3.95 [3.34; 4.67]
Adding Blumberg 2001 (k=7) 3.88 [3.29; 4.56]
Adding Chow 2008 (k=8) 3.99 [3.40; 4.68]
Adding Hermsen 2011 (k=9) 3.83 [3.28; 4.48]
Adding Pasero 2011 (k=10) 3.93 [3.37; 4.58]
Adding Ahmed, 2017 (k=11) 3.80 [3.26; 4.42]
Adding Presterl 2009 (k=12) 3.74 [3.22; 4.35]
Adding Tukenmez 2017 (k=13) 3.76 [3.24; 4.36]
Adding Hall 2013 (k=14) 3.71 [3.20; 4.30]
Adding Vardakas 2009 (k=15) 3.68 [3.18; 4.27]
Adding Posteraro 2011 (k=16) 3.70 [3.19; 4.28]
Adding Agvald−Ohman 2008 (k=17) 3.70 [3.20; 4.28]
Adding Paphitou 2005 (k=18) 3.73 [3.22; 4.31]
Adding Kautzky 2015 (k=19) 3.73 [3.23; 4.31]
Fixed effect model 3.73 [3.23; 4.31]
0.2
0.5
1
2
5

e-Figure 23

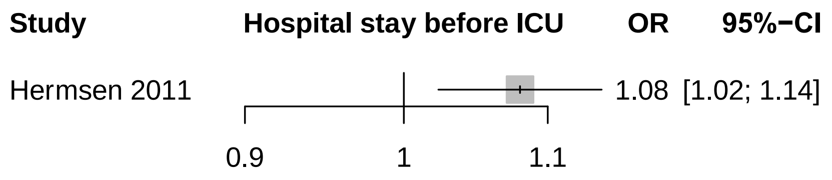

14 days [7-25.5] vs. 7 [3-12] ($p<0.01$) in Ahmed 2017
4.6±5.6 days vs. 3.0±3.3 (NS) in Peres-Bota 2004

Total days of stay including ICU:
35.4±3.0 days vs. 21.4±1.8 ($p=0.001$) in Ortiz-Ruiz 2016

e-Figure 24

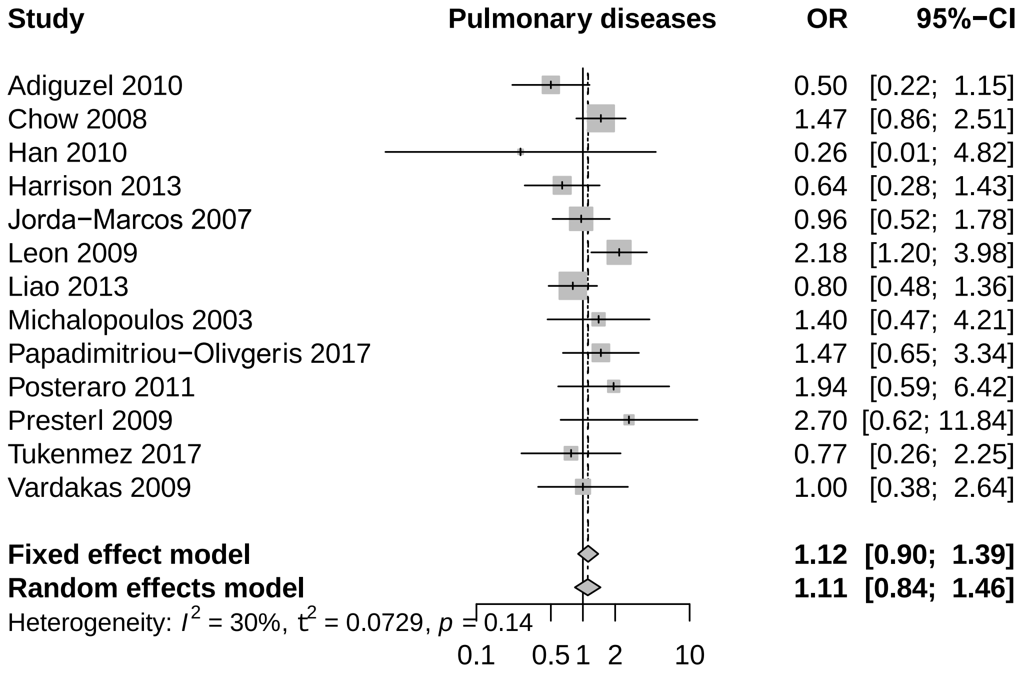

Only COPD in Adiguzel 2010, Han 2010, Leon 2009, Liao 2010 & Michalopoulos 2003

e-Figure 25

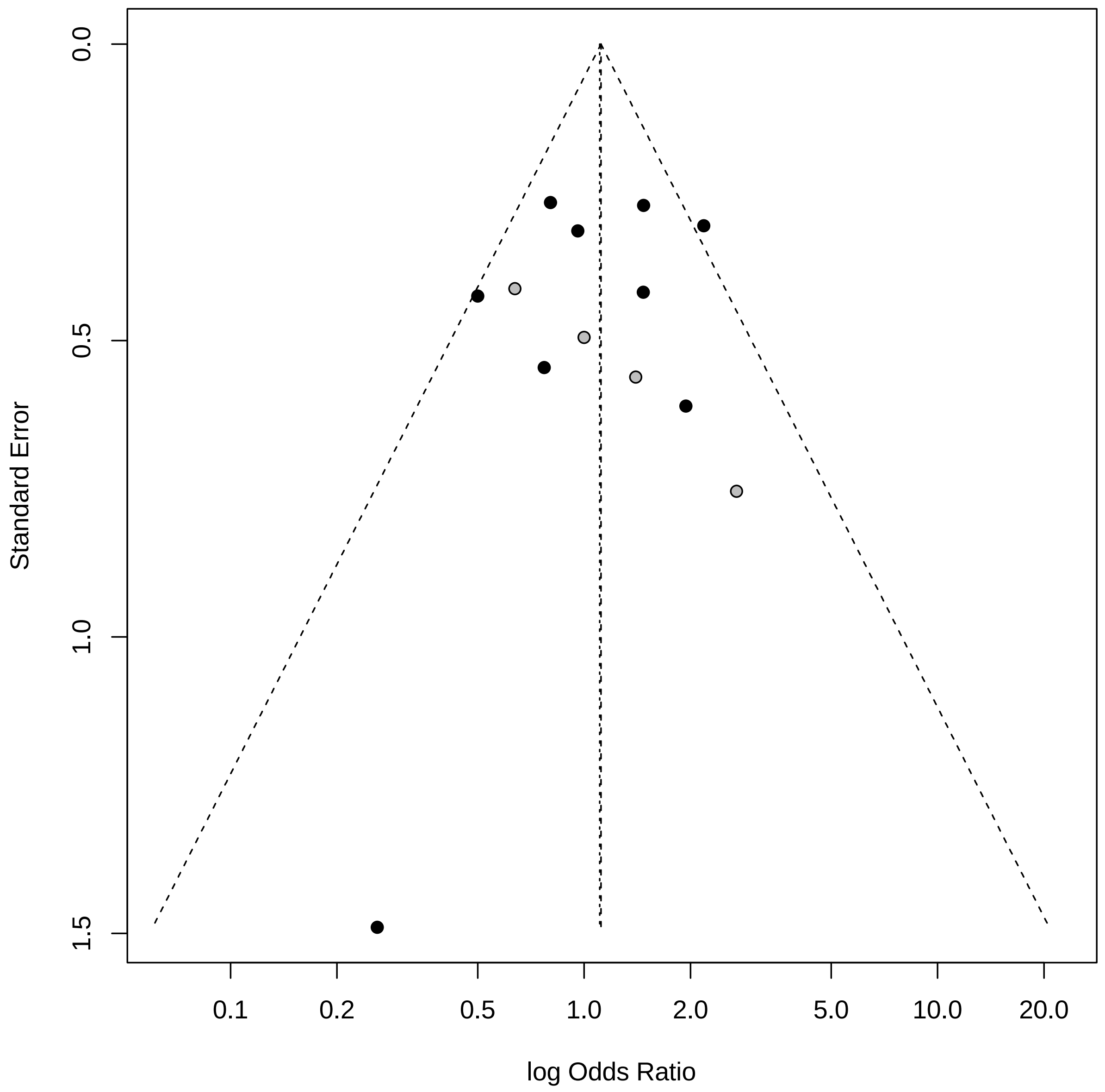

e-Figure 26

```
## [1] "p-value for Funnel-plot asymmetry (Eggers-test):  0.765"
```

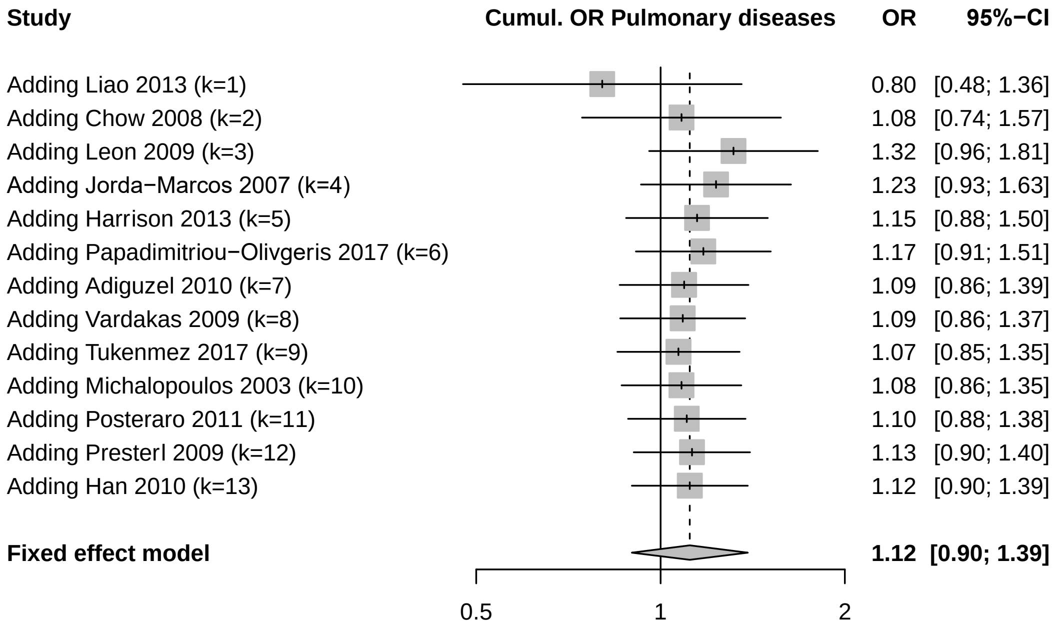
Study
Cumul. OR Pulmonary diseases
OR
95%−CI
Adding Liao 2013 (k=1)
0.80 [0.48; 1.36]
Adding Chow 2008 (k=2)
1.08 [0.74; 1.57]
Adding Leon 2009 (k=3)
1.32 [0.96; 1.81]
Adding Jorda−Marcos 2007 (k=4)
1.23 [0.93; 1.63]
Adding Harrison 2013 (k=5)
1.15 [0.88; 1.50]
Adding Papadimitriou−Olivgeris 2017 (k=6)
1.17 [0.91; 1.51]
Adding Adiguzel 2010 (k=7)
1.09 [0.86; 1.39]
Adding Vardakas 2009 (k=8)
1.09 [0.86; 1.37]
Adding Tukenmez 2017 (k=9)
1.07 [0.85; 1.35]
Adding Michalopoulos 2003 (k=10)
1.08 [0.86; 1.35]
Adding Posteraro 2011 (k=11)
1.10 [0.88; 1.38]
Adding Presterl 2009 (k=12)
1.13 [0.90; 1.40]
Adding Han 2010 (k=13)
1.12 [0.90; 1.39]
Fixed effect model
1.12 [0.90; 1.39]
0.5
1
2

e-Figure 27

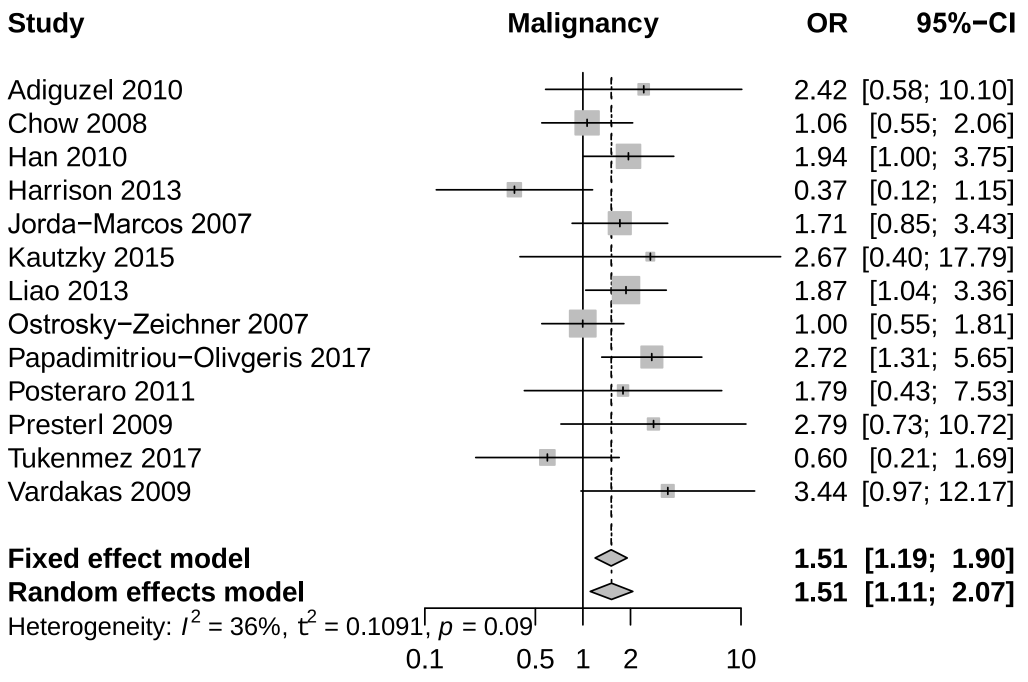
Study
Malignancy
OR
95%−CI
Adiguzel 2010 2.42 [0.58; 10.10]
Chow 2008 1.06 [0.55; 2.06]
Han 2010 1.94 [1.00; 3.75]
Harrison 2013 0.37 [0.12; 1.15]
Jorda−Marcos 2007 1.71 [0.85; 3.43]
Kautzky 2015 2.67 [0.40; 17.79]
Liao 2013 1.87 [1.04; 3.36]
Ostrosky−Zeichner 2007 1.00 [0.55; 1.81]
Papadimitriou−Olivgeris 2017 2.72 [1.31; 5.65]
Posteraro 2011 1.79 [0.43; 7.53]
Presterl 2009 2.79 [0.73; 10.72]
Tukenmez 2017 0.60 [0.21; 1.69]
Vardakas 2009 3.44 [0.97; 12.17]
Fixed effect model 1.51 [1.19; 1.90]
Random effects model 1.51 [1.11; 2.07]
Heterogeneity: I^2 = 36%, τ^2 = 0.1091, p = 0.09
0.1
0.5
1
2
10

e-Figure 28

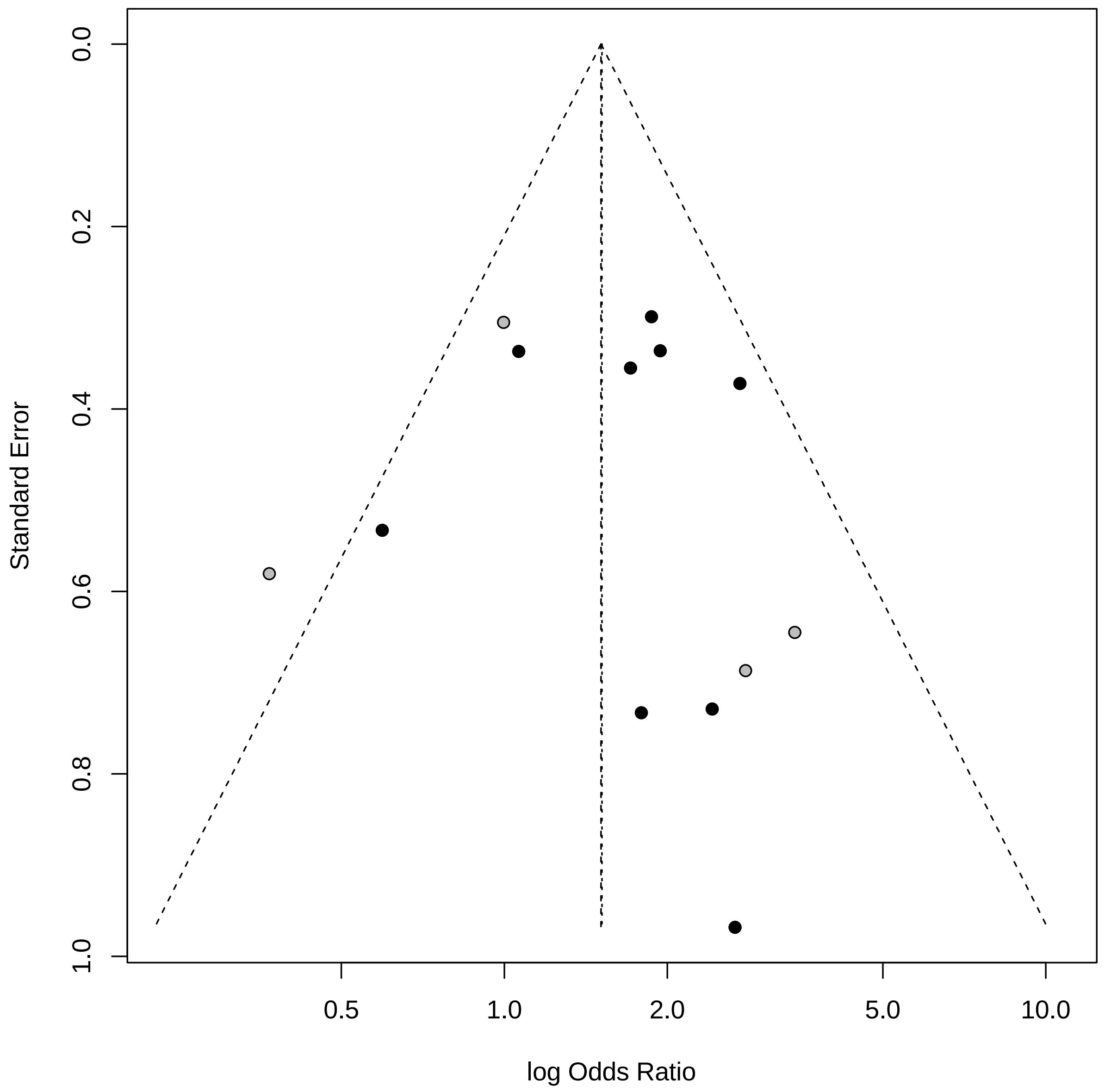

e-Figure 29

```
## [1] "p-value for Funnel-plot asymmetry (Eggers-test):  0.771"
```

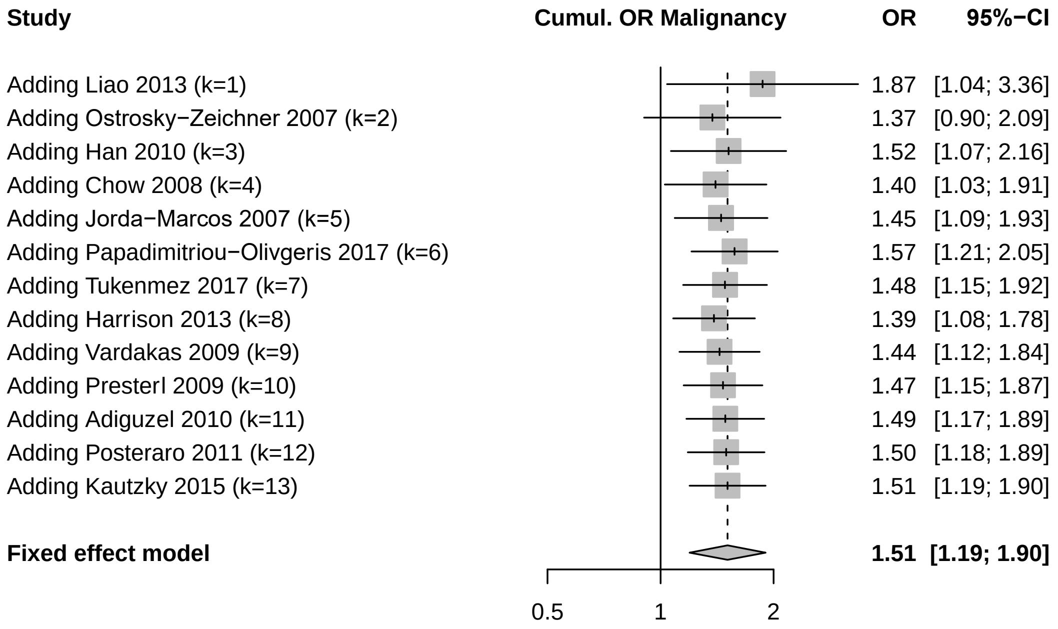
Study
Cumul. OR Malignancy
OR
95%−CI
Adding Liao 2013 (k=1) 1.87 [1.04; 3.36]
Adding Ostrosky−Zeichner 2007 (k=2) 1.37 [0.90; 2.09]
Adding Han 2010 (k=3) 1.52 [1.07; 2.16]
Adding Chow 2008 (k=4) 1.40 [1.03; 1.91]
Adding Jorda−Marcos 2007 (k=5) 1.45 [1.09; 1.93]
Adding Papadimitriou−Olivgeris 2017 (k=6) 1.57 [1.21; 2.05]
Adding Tukenmez 2017 (k=7) 1.48 [1.15; 1.92]
Adding Harrison 2013 (k=8) 1.39 [1.08; 1.78]
Adding Vardakas 2009 (k=9) 1.44 [1.12; 1.84]
Adding Presterl 2009 (k=10) 1.47 [1.15; 1.87]
Adding Adiguzel 2010 (k=11) 1.49 [1.17; 1.89]
Adding Posteraro 2011 (k=12) 1.50 [1.18; 1.89]
Adding Kautzky 2015 (k=13) 1.51 [1.19; 1.90]
Fixed effect model 1.51 [1.19; 1.90]
0.5
1
2

e-Figure 30

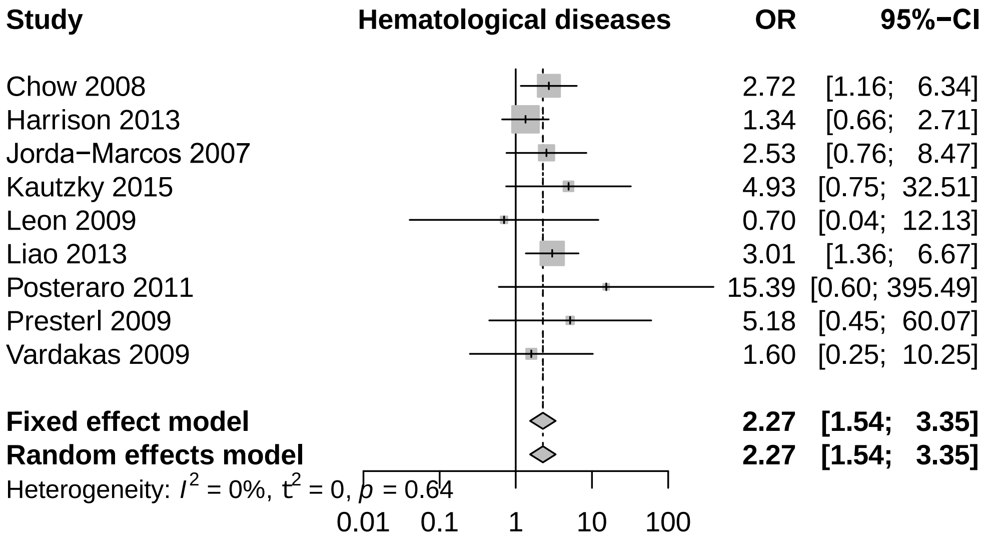

Study
Hematological diseases
OR
95%−CI
Chow 2008
2.72 [1.16; 6.34]
Harrison 2013
1.34 [0.66; 2.71]
Jorda−Marcos 2007
2.53 [0.76; 8.47]
Kautzky 2015
4.93 [0.75; 32.51]
Leon 2009
0.70 [0.04; 12.13]
Liao 2013
3.01 [1.36; 6.67]
Posteraro 2011
15.39 [0.60; 395.49]
Presterl 2009
5.18 [0.45; 60.07]
Vardakas 2009
1.60 [0.25; 10.25]
Fixed effect model
2.27 [1.54; 3.35]
Random effects model
2.27 [1.54; 3.35]
Heterogeneity: I^2 = 0%, τ^2 = 0, p = 0.64
0.01
0.1
1
10
100

e-Figure 31

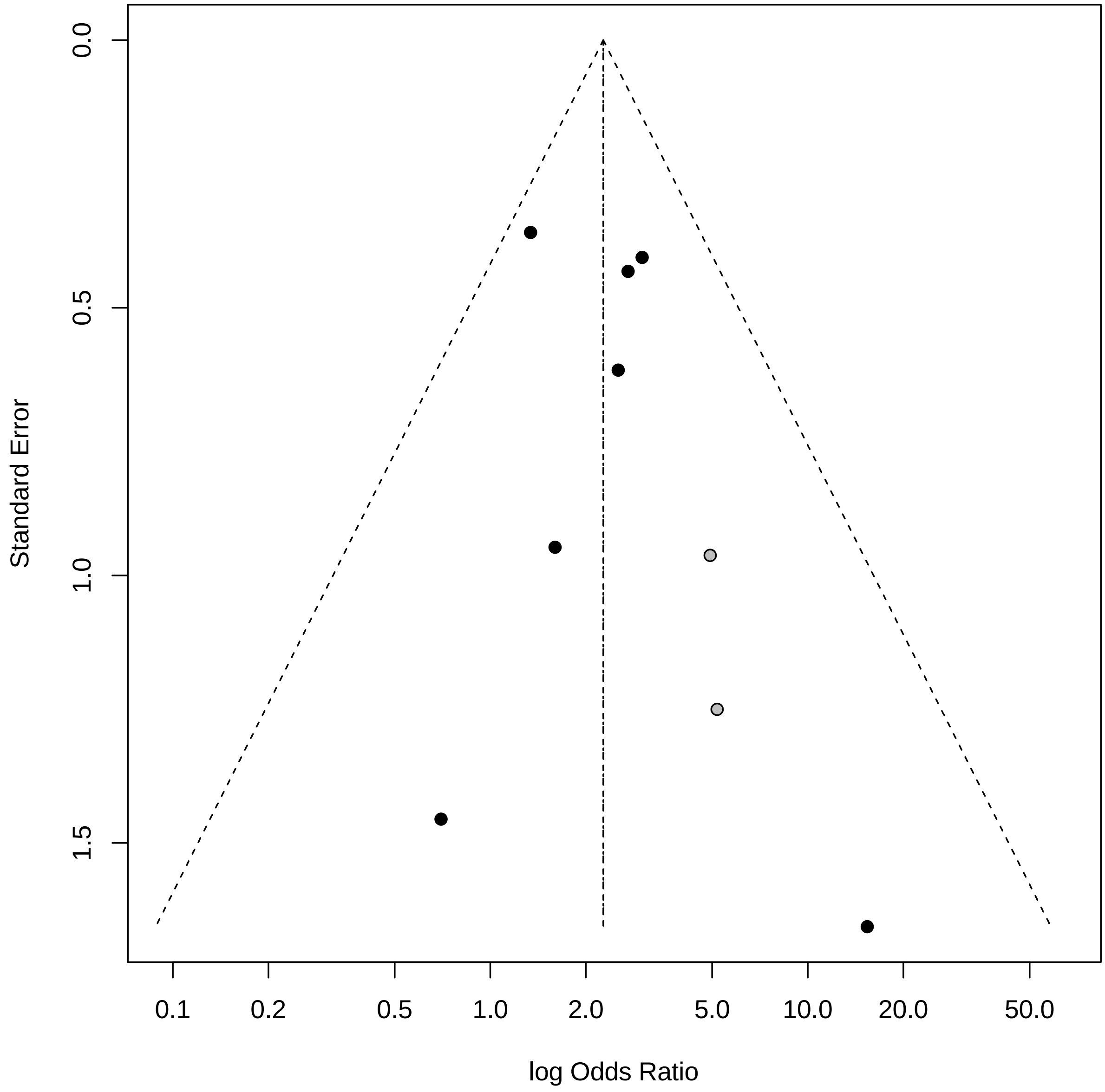

e-Figure 32

```
## [1] "Eggers test not available for less that 10 studies"
```

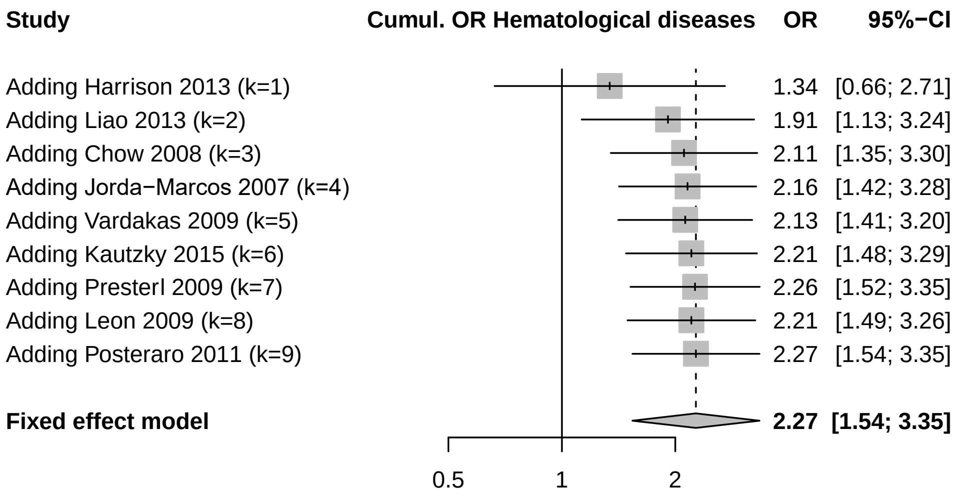
Study
Cumul. OR Hematological diseases
OR
95%−CI
Adding Harrison 2013 (k=1)
1.34
[0.66; 2.71]
Adding Liao 2013 (k=2)
1.91
[1.13; 3.24]
Adding Chow 2008 (k=3)
2.11
[1.35; 3.30]
Adding Jorda−Marcos 2007 (k=4)
2.16
[1.42; 3.28]
Adding Vardakas 2009 (k=5)
2.13
[1.41; 3.20]
Adding Kautzky 2015 (k=6)
2.21
[1.48; 3.29]
Adding Presterl 2009 (k=7)
2.26
[1.52; 3.35]
Adding Leon 2009 (k=8)
2.21
[1.49; 3.26]
Adding Posteraro 2011 (k=9)
2.27
[1.54; 3.35]
Fixed effect model
2.27
[1.54; 3.35]
0.5
1
2

e-Figure 33

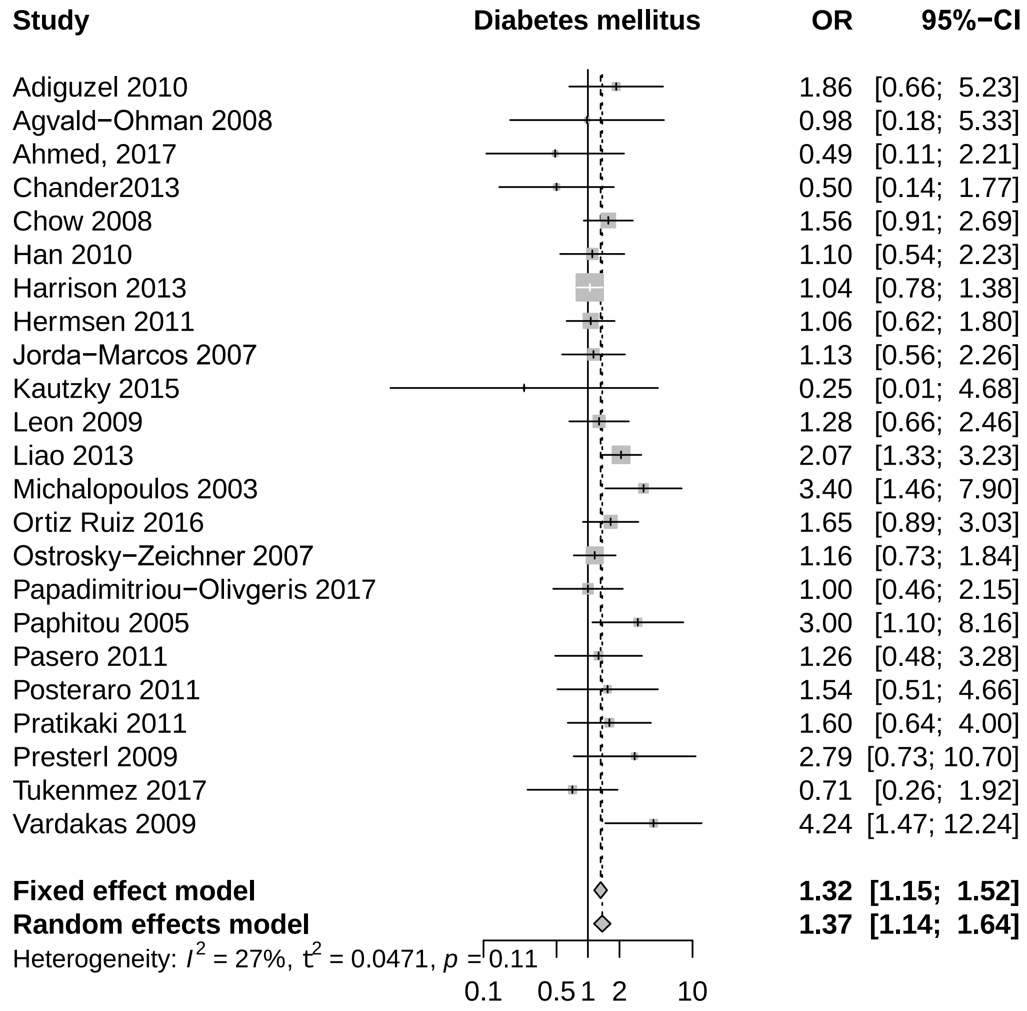
Study
Diabetes mellitus
OR
95%−CI
Adiguzel 2010 1.86 [0.66; 5.23]
Agvald−Ohman 2008 0.98 [0.18; 5.33]
Ahmed, 2017 0.49 [0.11; 2.21]
Chander2013 0.50 [0.14; 1.77]
Chow 2008 1.56 [0.91; 2.69]
Han 2010 1.10 [0.54; 2.23]
Harrison 2013 1.04 [0.78; 1.38]
Hermsen 2011 1.06 [0.62; 1.80]
Jorda−Marcos 2007 1.13 [0.56; 2.26]
Kautzky 2015 0.25 [0.01; 4.68]
Leon 2009 1.28 [0.66; 2.46]
Liao 2013 2.07 [1.33; 3.23]
Michalopoulos 2003 3.40 [1.46; 7.90]
Ortiz Ruiz 2016 1.65 [0.89; 3.03]
Ostrosky−Zeichner 2007 1.16 [0.73; 1.84]
Papadimitriou−Olivgeris 2017 1.00 [0.46; 2.15]
Paphitou 2005 3.00 [1.10; 8.16]
Pasero 2011 1.26 [0.48; 3.28]
Posteraro 2011 1.54 [0.51; 4.66]
Pratikaki 2011 1.60 [0.64; 4.00]
Presterl 2009 2.79 [0.73; 10.70]
Tukenmez 2017 0.71 [0.26; 1.92]
Vardakas 2009 4.24 [1.47; 12.24]
Fixed effect model 1.32 [1.15; 1.52]
Random effects model 1.37 [1.14; 1.64]
Heterogeneity: I^2 = 27%, τ^2 = 0.0471, p = 0.11
0.1 0.5 1 2 10

e-Figure 34

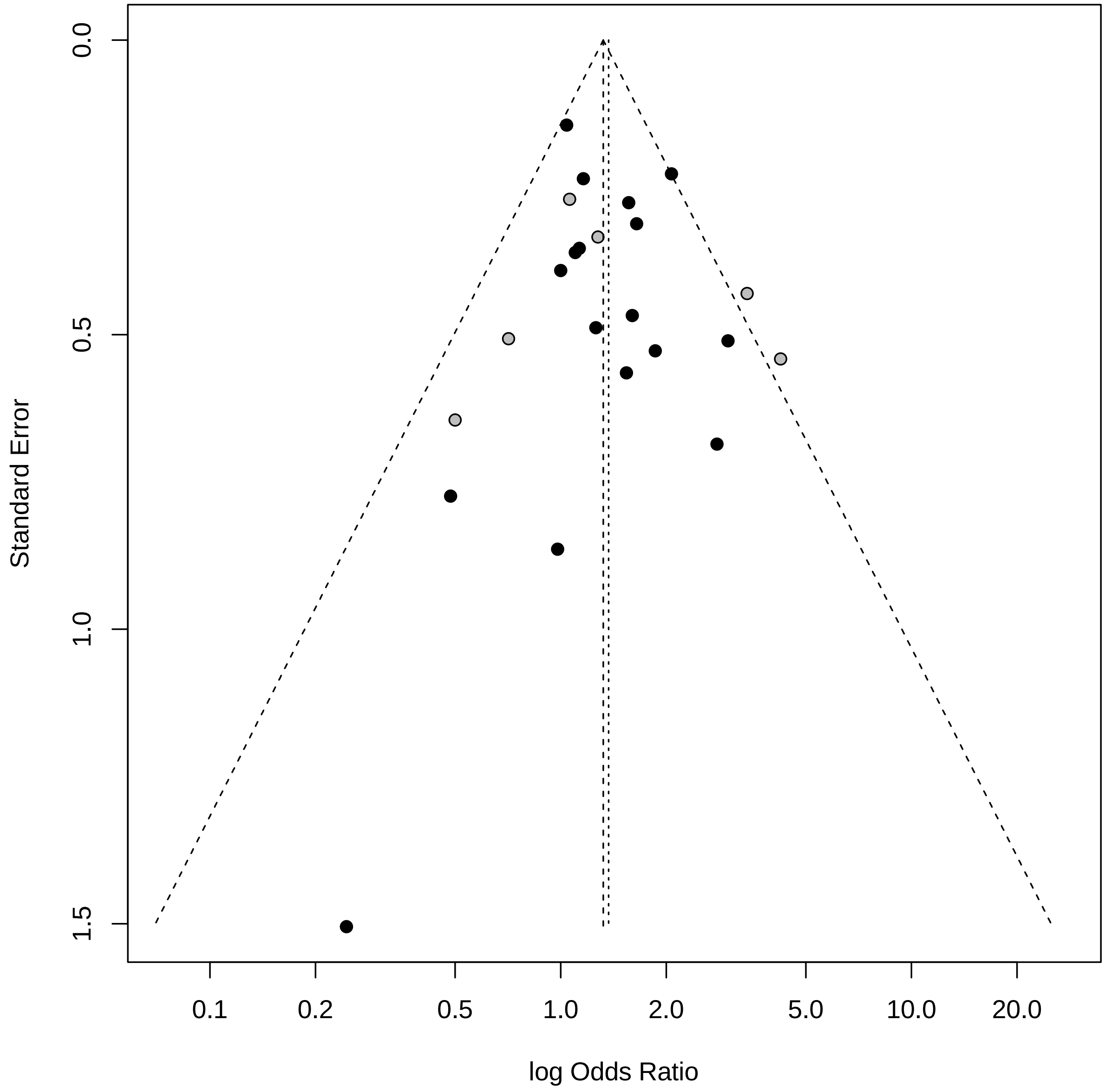

e-Figure 35

```
## [1] "p-value for Funnel-plot asymmetry (Eggers-test):  0.642"
```

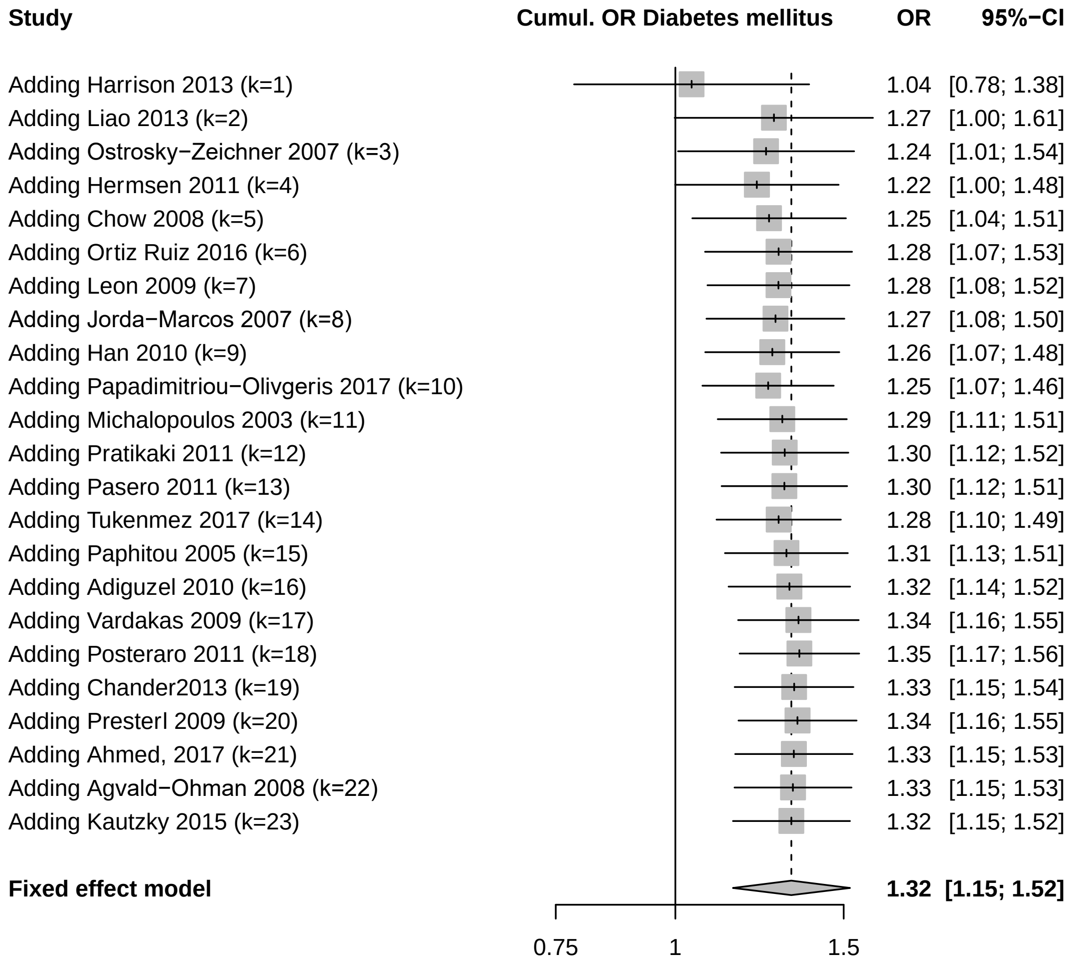
Study
Cumul. OR Diabetes mellitus
OR
95%−CI
Adding Harrison 2013 (k=1) 1.04 [0.78; 1.38]
Adding Liao 2013 (k=2) 1.27 [1.00; 1.61]
Adding Ostrosky−Zeichner 2007 (k=3) 1.24 [1.01; 1.54]
Adding Hermsen 2011 (k=4) 1.22 [1.00; 1.48]
Adding Chow 2008 (k=5) 1.25 [1.04; 1.51]
Adding Ortiz Ruiz 2016 (k=6) 1.28 [1.07; 1.53]
Adding Leon 2009 (k=7) 1.28 [1.08; 1.52]
Adding Jorda−Marcos 2007 (k=8) 1.27 [1.08; 1.50]
Adding Han 2010 (k=9) 1.26 [1.07; 1.48]
Adding Papadimitriou−Olivgeris 2017 (k=10) 1.25 [1.07; 1.46]
Adding Michalopoulos 2003 (k=11) 1.29 [1.11; 1.51]
Adding Pratikaki 2011 (k=12) 1.30 [1.12; 1.52]
Adding Pasero 2011 (k=13) 1.30 [1.12; 1.51]
Adding Tukenmez 2017 (k=14) 1.28 [1.10; 1.49]
Adding Paphitou 2005 (k=15) 1.31 [1.13; 1.51]
Adding Adiguzel 2010 (k=16) 1.32 [1.14; 1.52]
Adding Vardakas 2009 (k=17) 1.34 [1.16; 1.55]
Adding Posteraro 2011 (k=18) 1.35 [1.17; 1.56]
Adding Chander2013 (k=19) 1.33 [1.15; 1.54]
Adding Presterl 2009 (k=20) 1.34 [1.16; 1.55]
Adding Ahmed, 2017 (k=21) 1.33 [1.15; 1.53]
Adding Agvald−Ohman 2008 (k=22) 1.33 [1.15; 1.53]
Adding Kautzky 2015 (k=23) 1.32 [1.15; 1.52]
Fixed effect model 1.32 [1.15; 1.52]
0.75
1
1.5

e-Figure 36

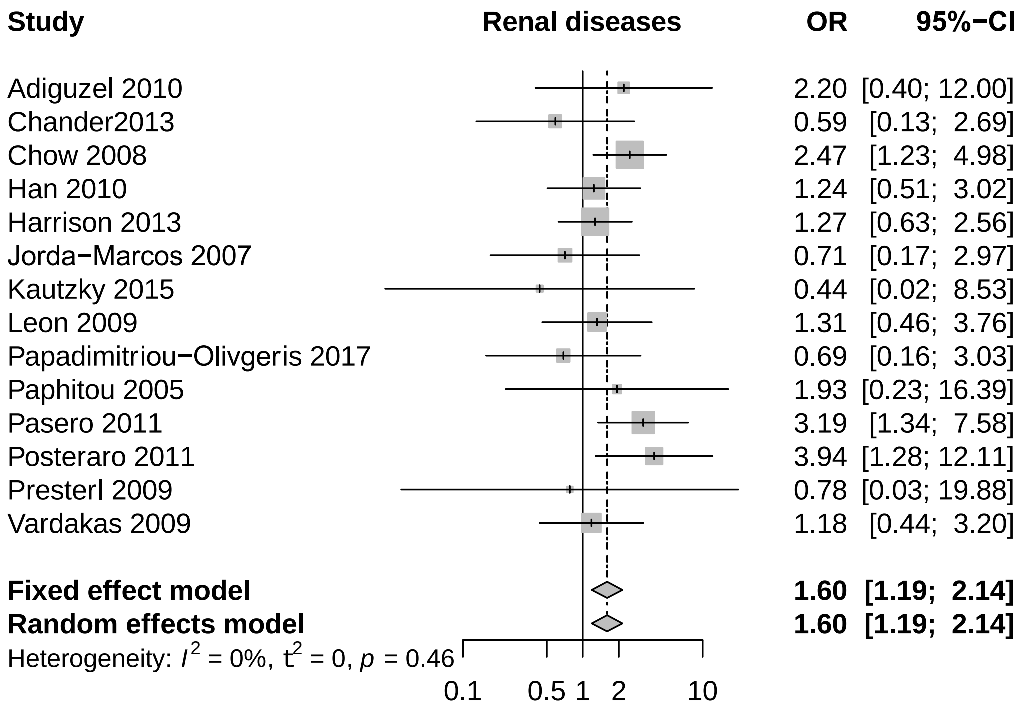

Creatinine > 2mg/dl in Pasero 2011
Chronic renal failure in Papadimitriou-Olivgeris 2017 & Posteraro 2011

e-Figure 37

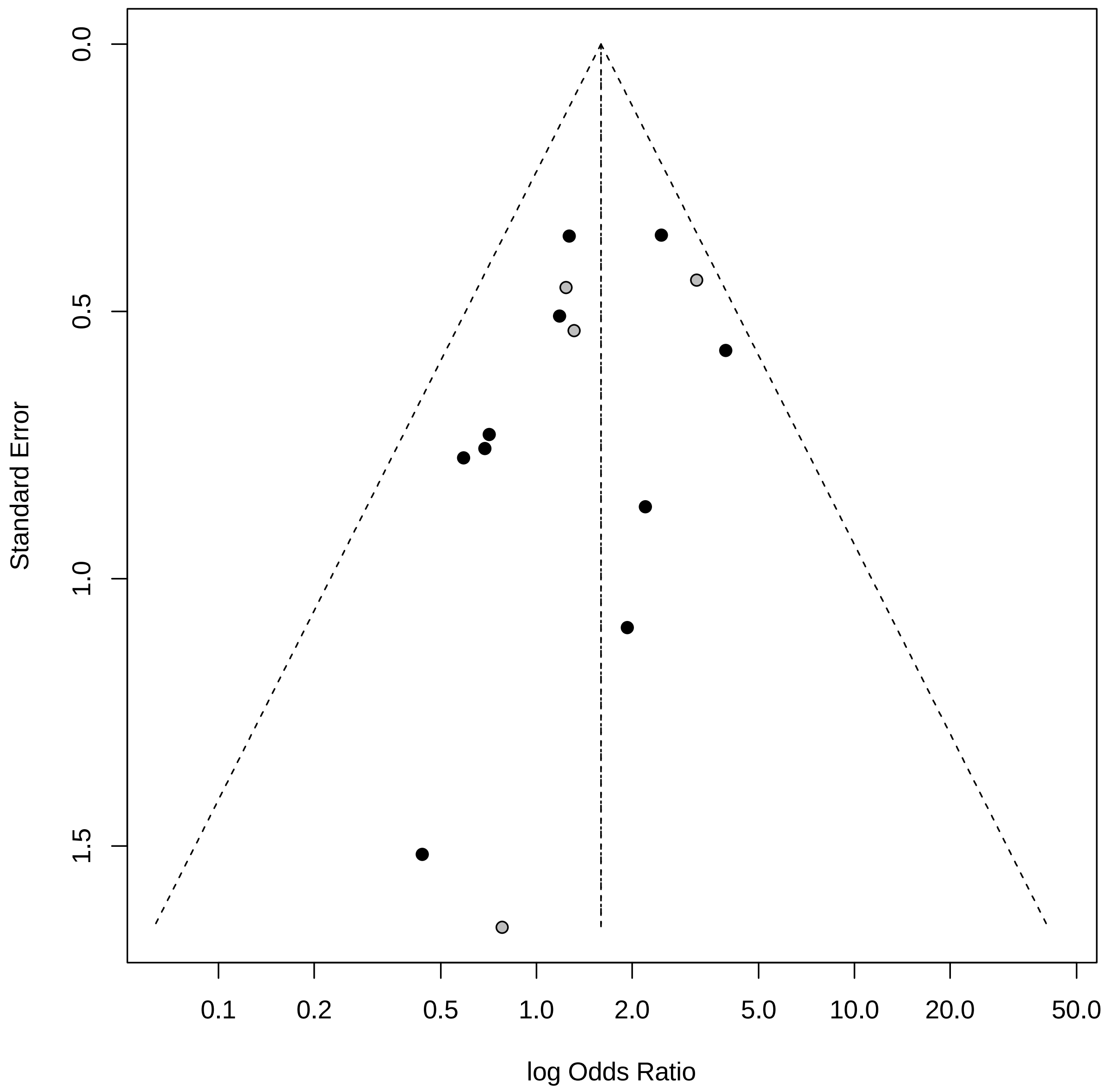

e-Figure 38

```
## [1] "p-value for Funnel-plot asymmetry (Eggers-test):  0.166"
```

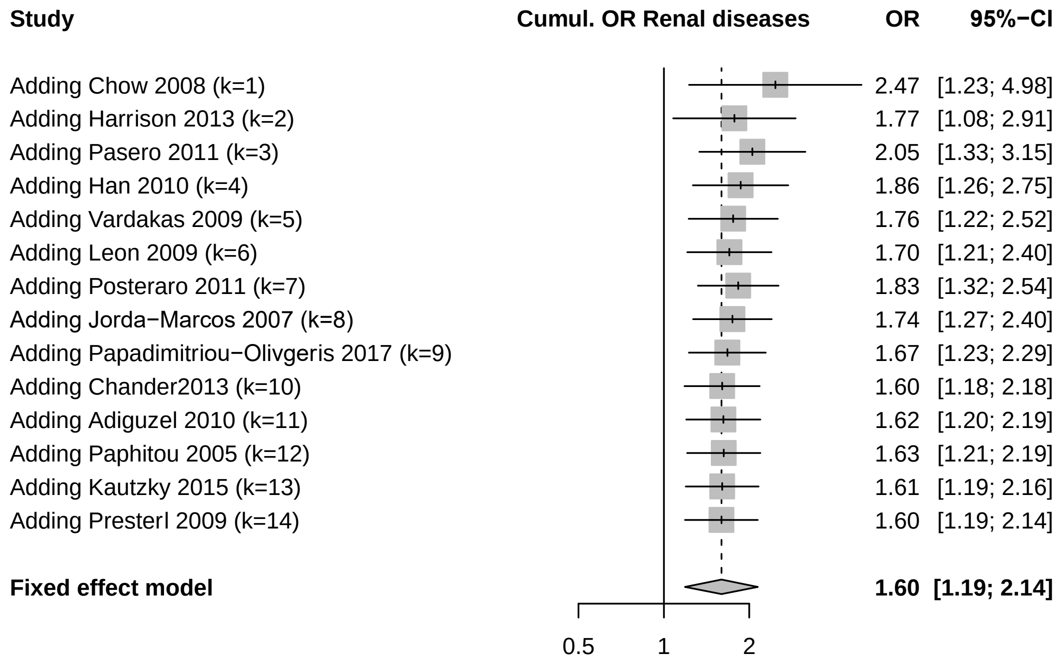
Study
Cumul. OR Renal diseases
OR
95%−CI
Adding Chow 2008 (k=1) 2.47 [1.23; 4.98]
Adding Harrison 2013 (k=2) 1.77 [1.08; 2.91]
Adding Pasero 2011 (k=3) 2.05 [1.33; 3.15]
Adding Han 2010 (k=4) 1.86 [1.26; 2.75]
Adding Vardakas 2009 (k=5) 1.76 [1.22; 2.52]
Adding Leon 2009 (k=6) 1.70 [1.21; 2.40]
Adding Posteraro 2011 (k=7) 1.83 [1.32; 2.54]
Adding Jorda−Marcos 2007 (k=8) 1.74 [1.27; 2.40]
Adding Papadimitriou−Olivgeris 2017 (k=9) 1.67 [1.23; 2.29]
Adding Chander2013 (k=10) 1.60 [1.18; 2.18]
Adding Adiguzel 2010 (k=11) 1.62 [1.20; 2.19]
Adding Paphitou 2005 (k=12) 1.63 [1.21; 2.19]
Adding Kautzky 2015 (k=13) 1.61 [1.19; 2.16]
Adding Presterl 2009 (k=14) 1.60 [1.19; 2.14]
Fixed effect model 1.60 [1.19; 2.14]
0.5
1
2

e-Figure 39

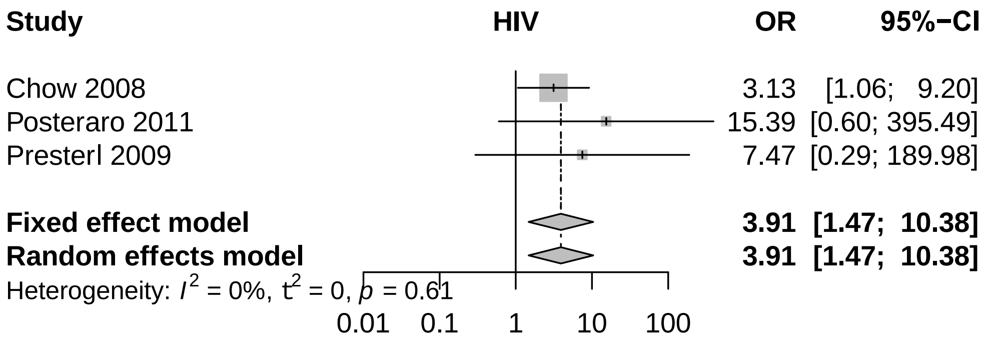

Study
HIV
OR
95%−CI
Chow 2008
3.13 [1.06; 9.20]
Posteraro 2011
15.39 [0.60; 395.49]
Presterl 2009
7.47 [0.29; 189.98]
Fixed effect model
3.91 [1.47; 10.38]
Random effects model
3.91 [1.47; 10.38]
Heterogeneity: I2 = 0%, τ2 = 0, p = 0.61
0.01
0.1
1
10
100

e-Figure 40

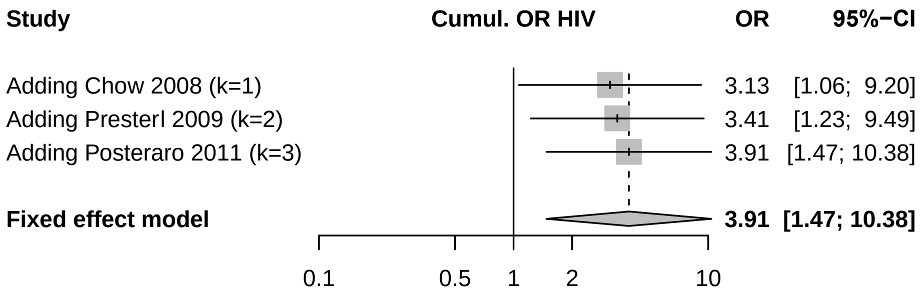
Study
Cumul. OR HIV
OR
95%−CI
Adding Chow 2008 (k=1)
3.13 [1.06; 9.20]
Adding Presterl 2009 (k=2)
3.41 [1.23; 9.49]
Adding Posteraro 2011 (k=3)
3.91 [1.47; 10.38]
Fixed effect model
3.91 [1.47; 10.38]
0.1
0.5
1
2
10

e-Figure 41

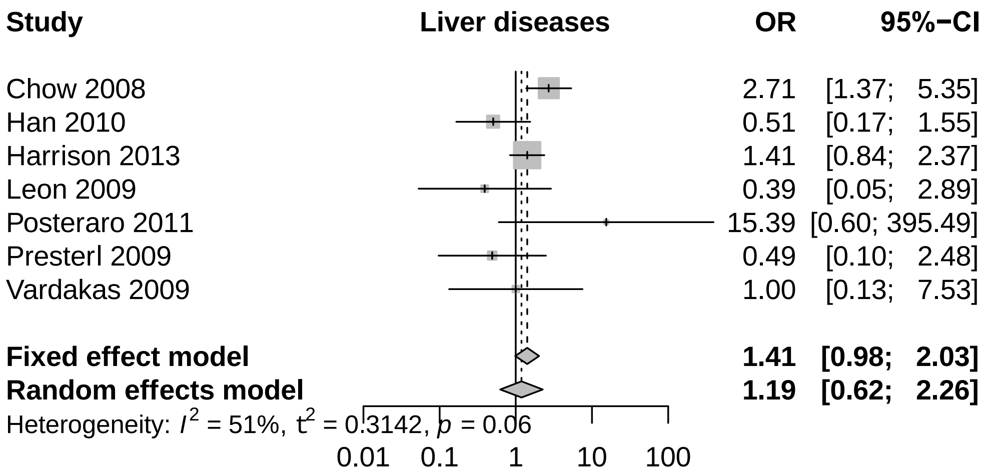

Only cirrhosis in Chowl 2009 & Posteraro 2011

e-Figure 42

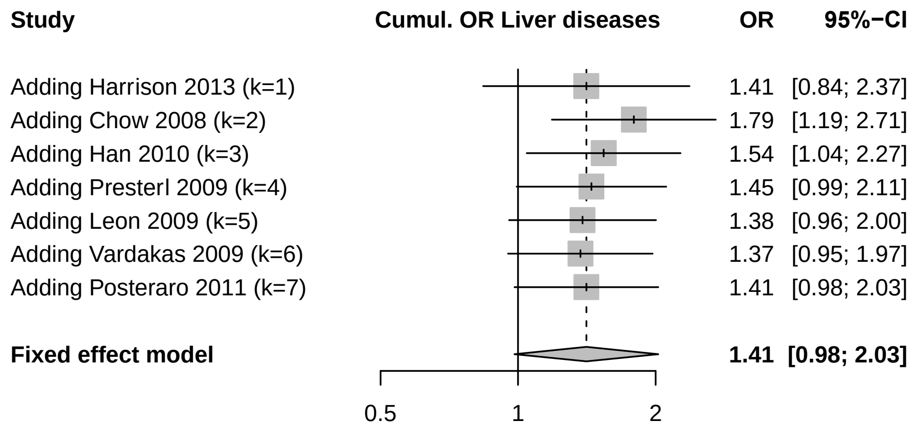
Study
Cumul. OR Liver diseases
OR
95%−CI
Adding Harrison 2013 (k=1)
1.41 [0.84; 2.37]
Adding Chow 2008 (k=2)
1.79 [1.19; 2.71]
Adding Han 2010 (k=3)
1.54 [1.04; 2.27]
Adding Presterl 2009 (k=4)
1.45 [0.99; 2.11]
Adding Leon 2009 (k=5)
1.38 [0.96; 2.00]
Adding Vardakas 2009 (k=6)
1.37 [0.95; 1.97]
Adding Posteraro 2011 (k=7)
1.41 [0.98; 2.03]
Fixed effect model
1.41 [0.98; 2.03]
0.5
1
2

e-Figure 43

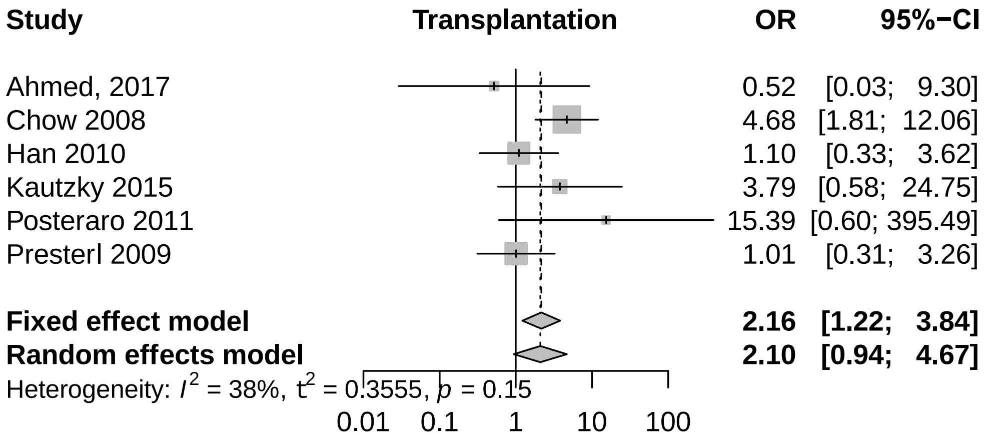
Study
Transplantation
OR
95%−CI
Ahmed, 2017 0.52 [0.03; 9.30]
Chow 2008 4.68 [1.81; 12.06]
Han 2010 1.10 [0.33; 3.62]
Kautzky 2015 3.79 [0.58; 24.75]
Posteraro 2011 15.39 [0.60; 395.49]
Presterl 2009 1.01 [0.31; 3.26]
Fixed effect model 2.16 [1.22; 3.84]
Random effects model 2.10 [0.94; 4.67]
Heterogeneity: I^2 = 38%, τ^2 = 0.3555, p = 0.15
0.01
0.1
1
10
100

e-Figure 44

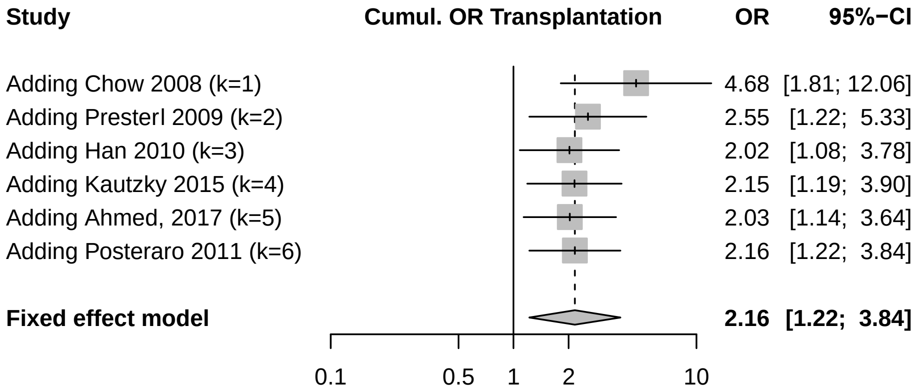
Study
Cumul. OR Transplantation
OR
95%−CI
Adding Chow 2008 (k=1)
Adding Presterl 2009 (k=2)
Adding Han 2010 (k=3)
Adding Kautzky 2015 (k=4)
Adding Ahmed, 2017 (k=5)
Adding Posteraro 2011 (k=6)
4.68 [1.81; 12.06]
2.55 [1.22; 5.33]
2.02 [1.08; 3.78]
2.15 [1.19; 3.90]
2.03 [1.14; 3.64]
2.16 [1.22; 3.84]
Fixed effect model
2.16 [1.22; 3.84]
0.1
0.5
1
2
10

e-Figure 45

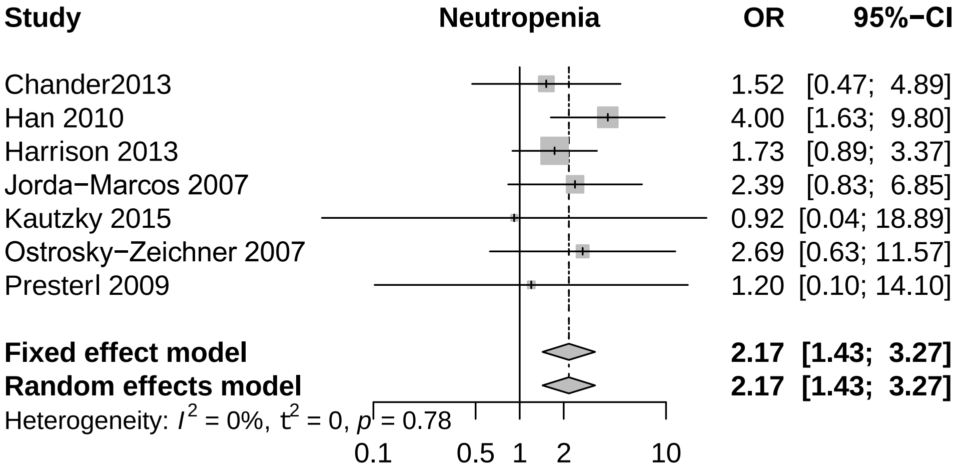
Study
Neutropenia
OR
95%−CI
Chander2013
1.52 [0.47; 4.89]
Han 2010
4.00 [1.63; 9.80]
Harrison 2013
1.73 [0.89; 3.37]
Jorda−Marcos 2007
2.39 [0.83; 6.85]
Kautzky 2015
0.92 [0.04; 18.89]
Ostrosky−Zeichner 2007
2.69 [0.63; 11.57]
Presterl 2009
1.20 [0.10; 14.10]
Fixed effect model
2.17 [1.43; 3.27]
Random effects model
2.17 [1.43; 3.27]
Heterogeneity: I^2 = 0%, τ^2 = 0, p = 0.78
0.1
0.5
1
2
10

e-Figure 46

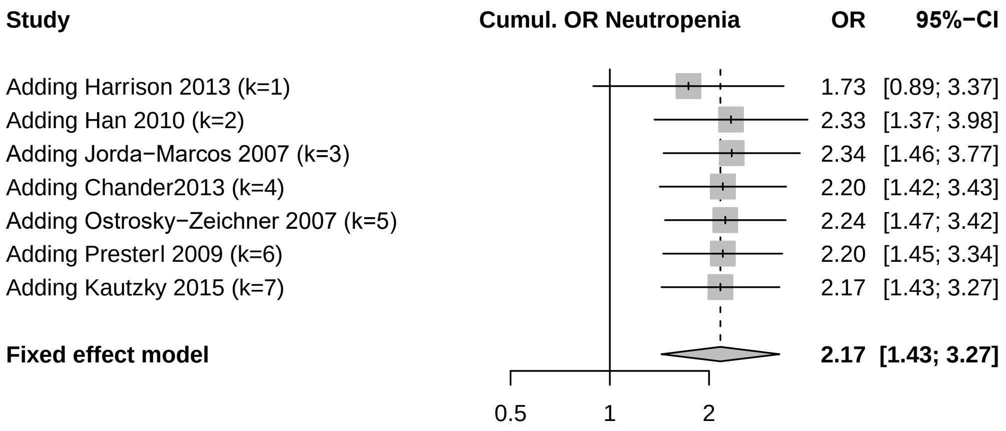
Study
Cumul. OR Neutropenia
OR
95%−CI
Adding Harrison 2013 (k=1)
1.73 [0.89; 3.37]
Adding Han 2010 (k=2)
2.33 [1.37; 3.98]
Adding Jorda−Marcos 2007 (k=3)
2.34 [1.46; 3.77]
Adding Chander2013 (k=4)
2.20 [1.42; 3.43]
Adding Ostrosky−Zeichner 2007 (k=5)
2.24 [1.47; 3.42]
Adding Presterl 2009 (k=6)
2.20 [1.45; 3.34]
Adding Kautzky 2015 (k=7)
2.17 [1.43; 3.27]
Fixed effect model
2.17 [1.43; 3.27]
0.5
1
2

e-Figure 47

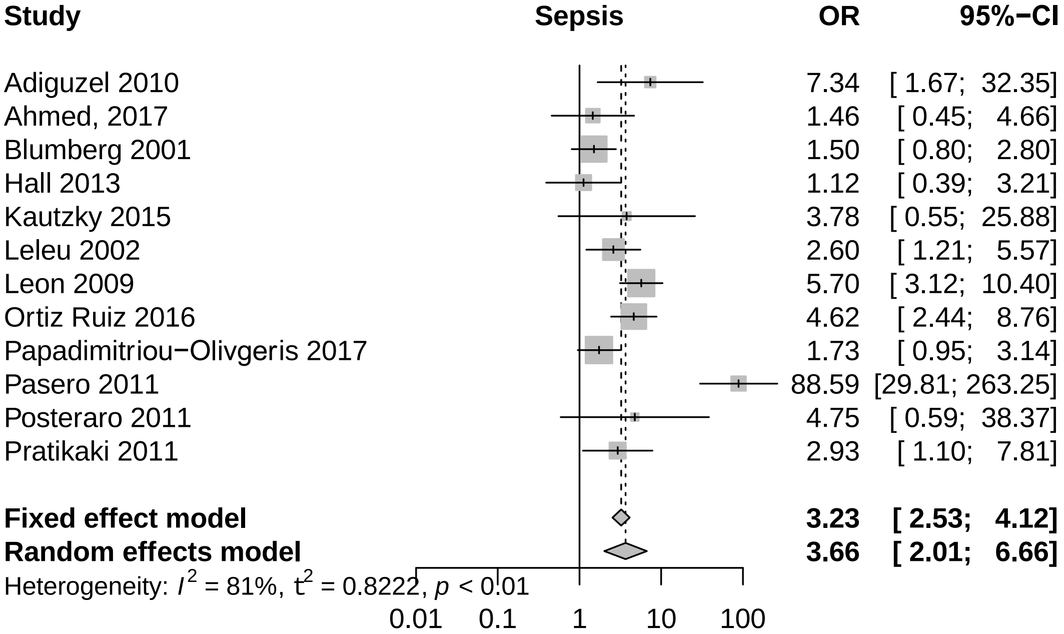

ARDS in Blumberg 2001 & Leleu 2002
severe sepsis in Hall 2013 & Pasero 2011
severe sepsis/septic shock in Leon 2009, Ortiz Ruiz 2016 & Posteraro 2011
KPC shock and sepsis in Papadimitriou-Olivgeris 2017

e-Figure 48

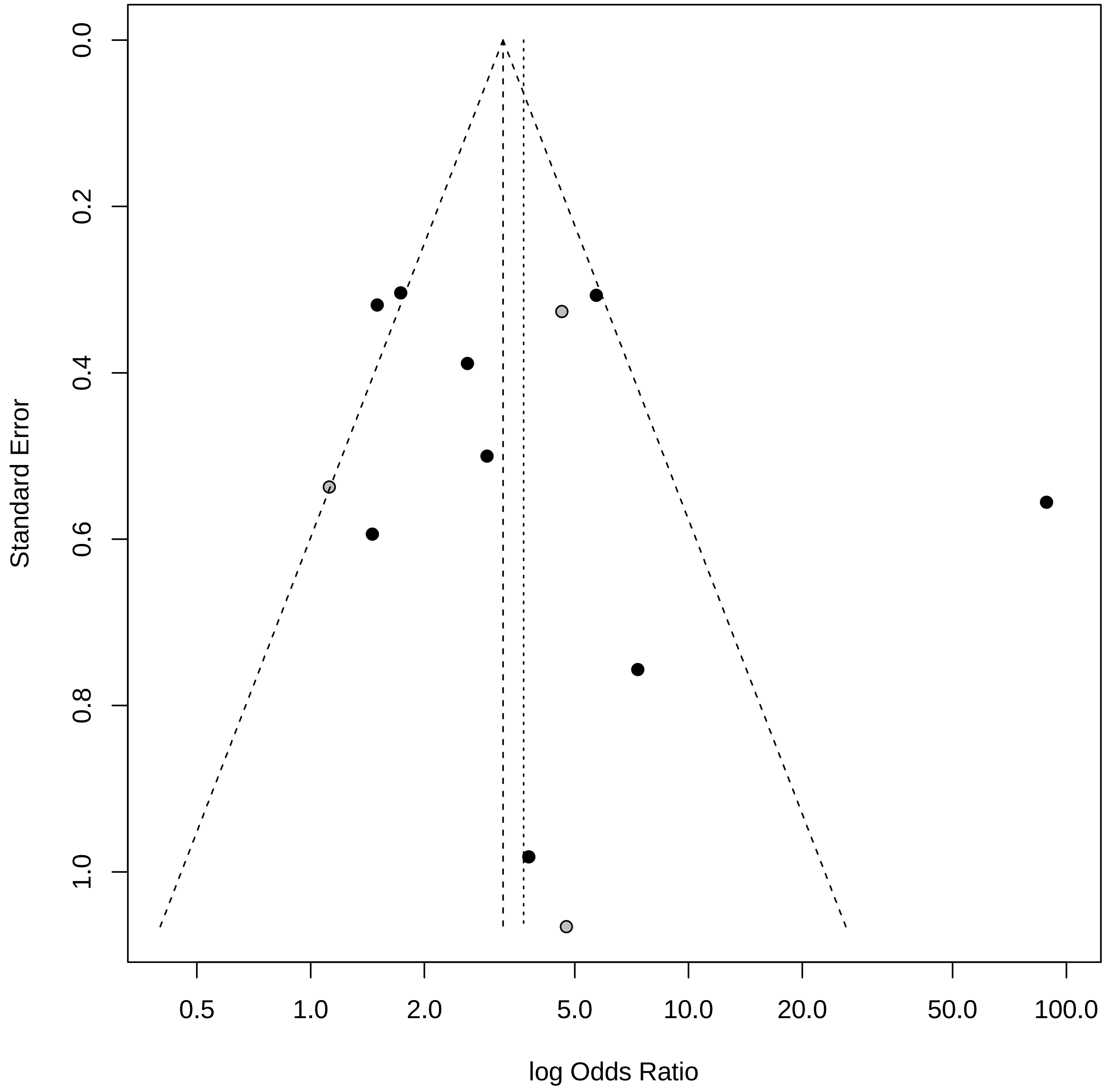

e-Figure 49

```
## [1] "p-value for Funnel-plot asymmetry (Eggers-test):  0.512"
```

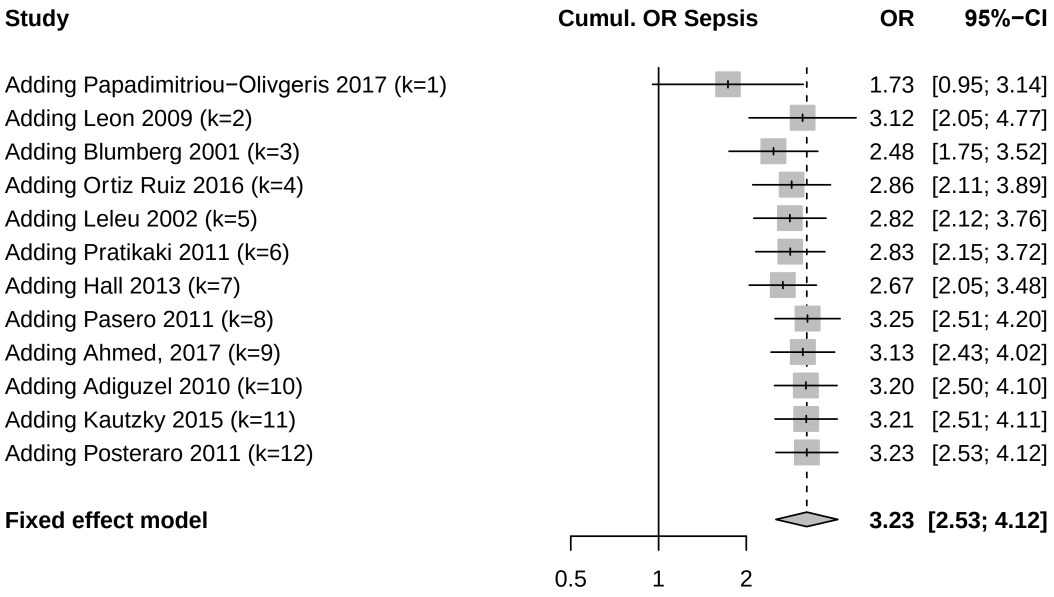
Study
Cumul. OR Sepsis
OR
95%−CI
Adding Papadimitriou−Olivgeris 2017 (k=1)
1.73 [0.95; 3.14]
Adding Leon 2009 (k=2)
3.12 [2.05; 4.77]
Adding Blumberg 2001 (k=3)
2.48 [1.75; 3.52]
Adding Ortiz Ruiz 2016 (k=4)
2.86 [2.11; 3.89]
Adding Leleu 2002 (k=5)
2.82 [2.12; 3.76]
Adding Pratikaki 2011 (k=6)
2.83 [2.15; 3.72]
Adding Hall 2013 (k=7)
2.67 [2.05; 3.48]
Adding Pasero 2011 (k=8)
3.25 [2.51; 4.20]
Adding Ahmed, 2017 (k=9)
3.13 [2.43; 4.02]
Adding Adiguzel 2010 (k=10)
3.20 [2.50; 4.10]
Adding Kautzky 2015 (k=11)
3.21 [2.51; 4.11]
Adding Posteraro 2011 (k=12)
3.23 [2.53; 4.12]
Fixed effect model
3.23 [2.53; 4.12]
0.5
1
2

e-Figure 50

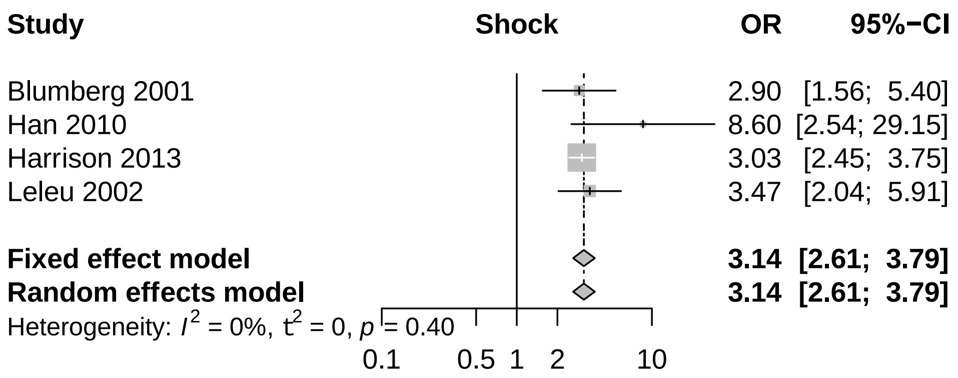

hemodynamic instability in Han 2010
advanced cardiovascular support in Harrison 2013

e-Figure 51

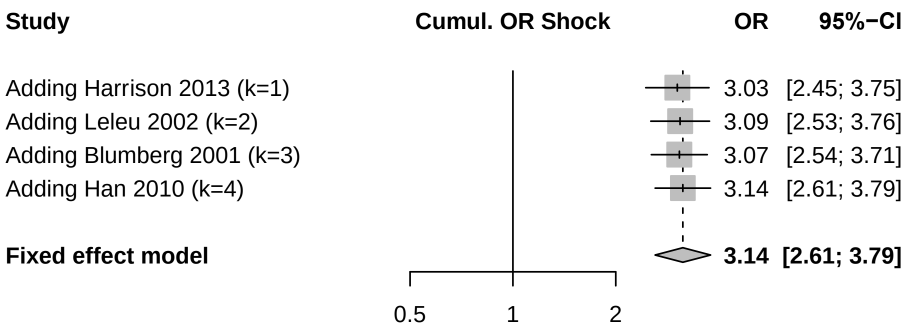
Study
Cumul. OR Shock
OR
95%−CI
Adding Harrison 2013 (k=1)
3.03 [2.45; 3.75]
Adding Leleu 2002 (k=2)
3.09 [2.53; 3.76]
Adding Blumberg 2001 (k=3)
3.07 [2.54; 3.71]
Adding Han 2010 (k=4)
3.14 [2.61; 3.79]
Fixed effect model
3.14 [2.61; 3.79]
0.5
1
2

e-Figure 52

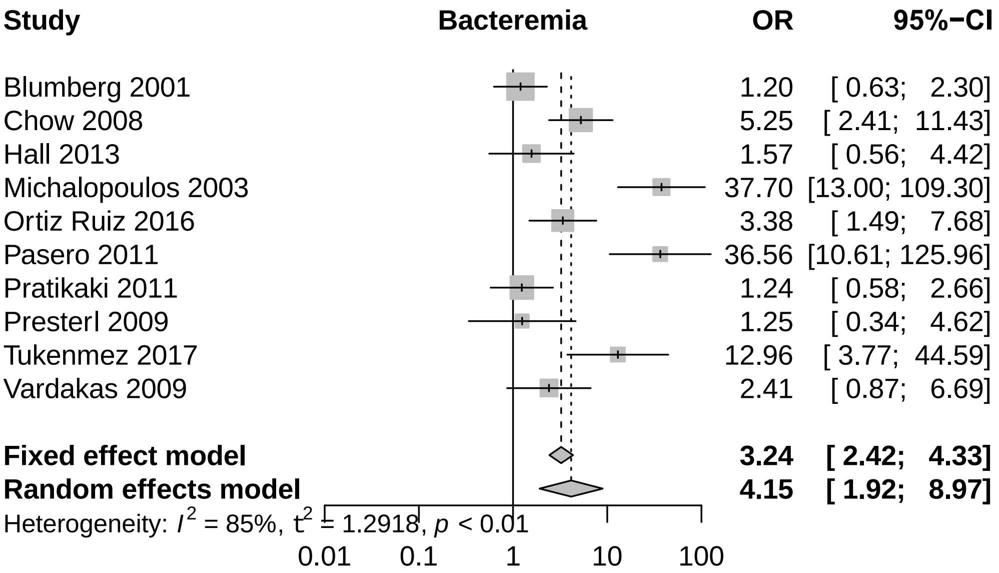
Study
Bacteremia
OR
95%−CI
Blumberg 2001
1.20
[0.63; 2.30]
Chow 2008
5.25
[2.41; 11.43]
Hall 2013
1.57
[0.56; 4.42]
Michalopoulos 2003
37.70
[13.00; 109.30]
Ortiz Ruiz 2016
3.38
[1.49; 7.68]
Pasero 2011
36.56
[10.61; 125.96]
Pratikaki 2011
1.24
[0.58; 2.66]
Presterl 2009
1.25
[0.34; 4.62]
Tukenmez 2017
12.96
[3.77; 44.59]
Vardakas 2009
2.41
[0.87; 6.69]
Fixed effect model
3.24
[2.42; 4.33]
Random effects model
4.15
[1.92; 8.97]
Heterogeneity: I^2 = 85%, τ^2 = 1.2918, p < 0.01
0.01
0.1
1
10
100

e-Figure 53

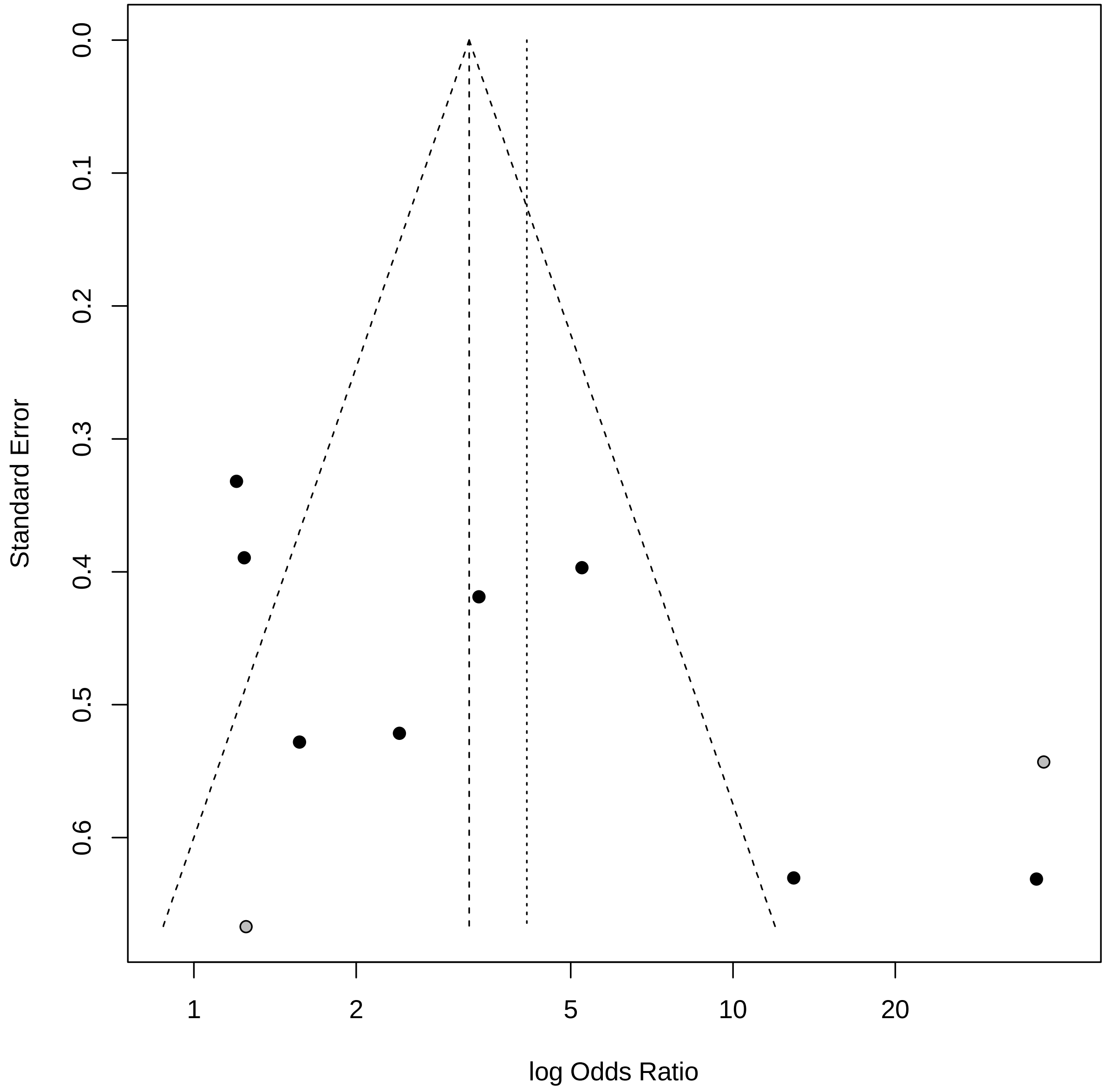

e-Figure 54

```
## [1] "p-value for Funnel-plot asymmetry (Eggers-test):  0.104"
```

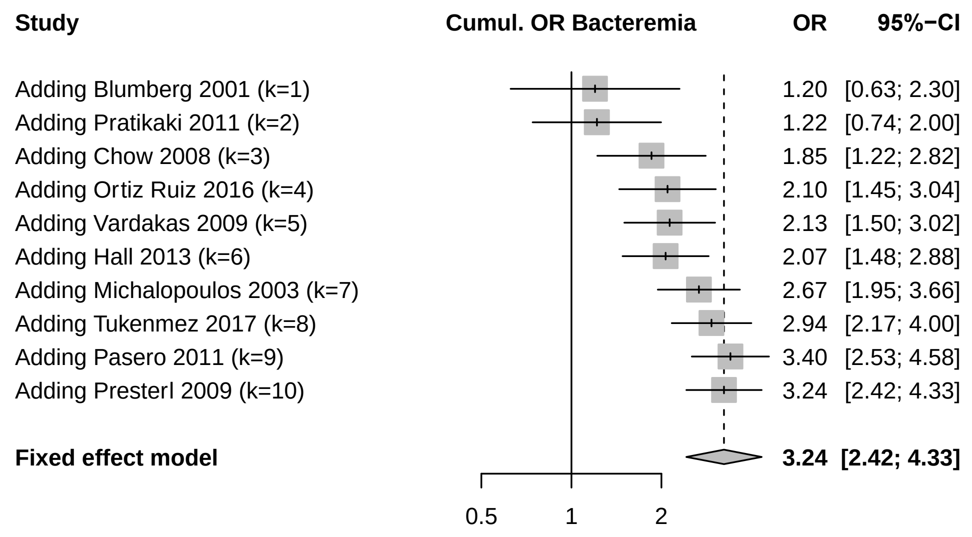
Study
Cumul. OR Bacteremia
OR
95%−CI
Adding Blumberg 2001 (k=1) 1.20 [0.63; 2.30]
Adding Pratikaki 2011 (k=2) 1.22 [0.74; 2.00]
Adding Chow 2008 (k=3) 1.85 [1.22; 2.82]
Adding Ortiz Ruiz 2016 (k=4) 2.10 [1.45; 3.04]
Adding Vardakas 2009 (k=5) 2.13 [1.50; 3.02]
Adding Hall 2013 (k=6) 2.07 [1.48; 2.88]
Adding Michalopoulos 2003 (k=7) 2.67 [1.95; 3.66]
Adding Tukenmez 2017 (k=8) 2.94 [2.17; 4.00]
Adding Pasero 2011 (k=9) 3.40 [2.53; 4.58]
Adding Presterl 2009 (k=10) 3.24 [2.42; 4.33]
Fixed effect model 3.24 [2.42; 4.33]
0.5
1
2

e-Figure 55

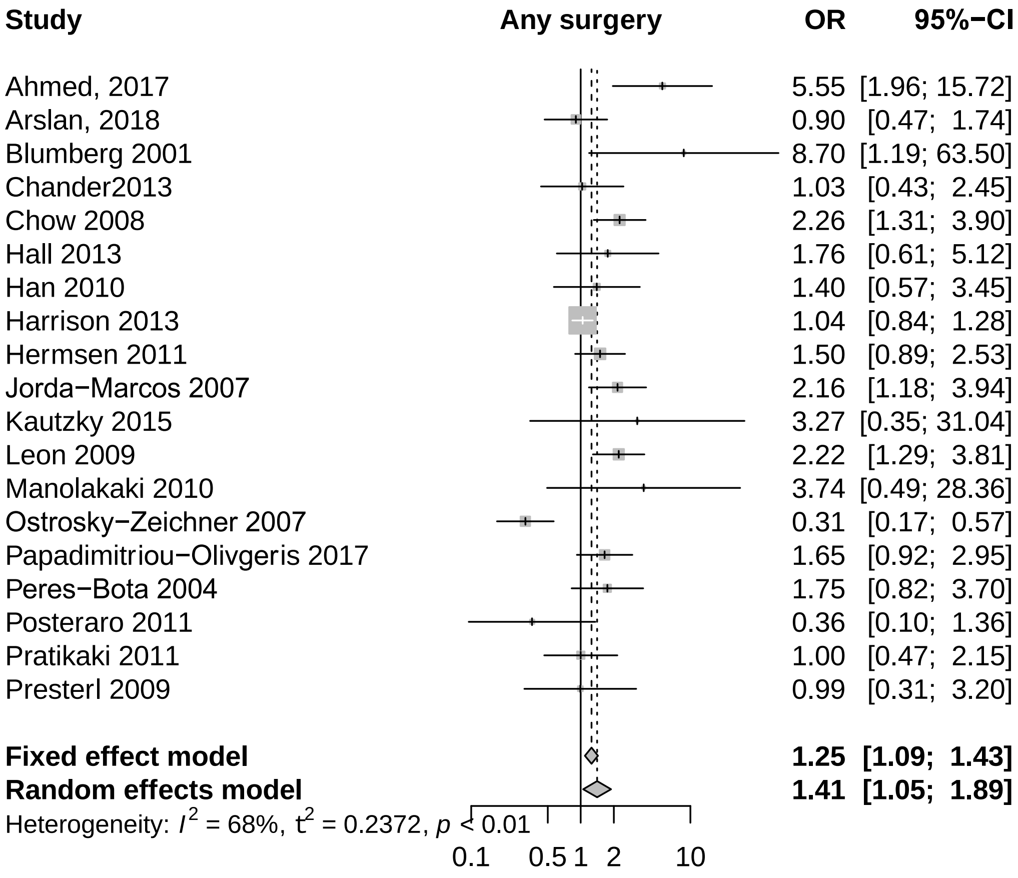

e-Figure 56

major surgery in Chander 2013
major pre-ICU operation in Chow 2008
day -7-3 used from Hermesen 2011
elective surgery in Jorda-Marcos 2007
at least 1 surgery in Leon 2009
d1-3 used from Ostrosky-Zeichner 2007
previous emergency surgery in Papadimitriou-Olivgeris 2017
trauma and surgical admission in Posteraro 2011

Ortiz-Ruiz 2016 reports an OR of 1.1 [0.7; 2.1] for other surgeries and 0.8 [0.5; 1.5] for cardiovascular surgery; as numbers with abdominal surgery add up to more than 100% no pooling was performed.

All patients had cardiac surgery in Michalopoulos 2003

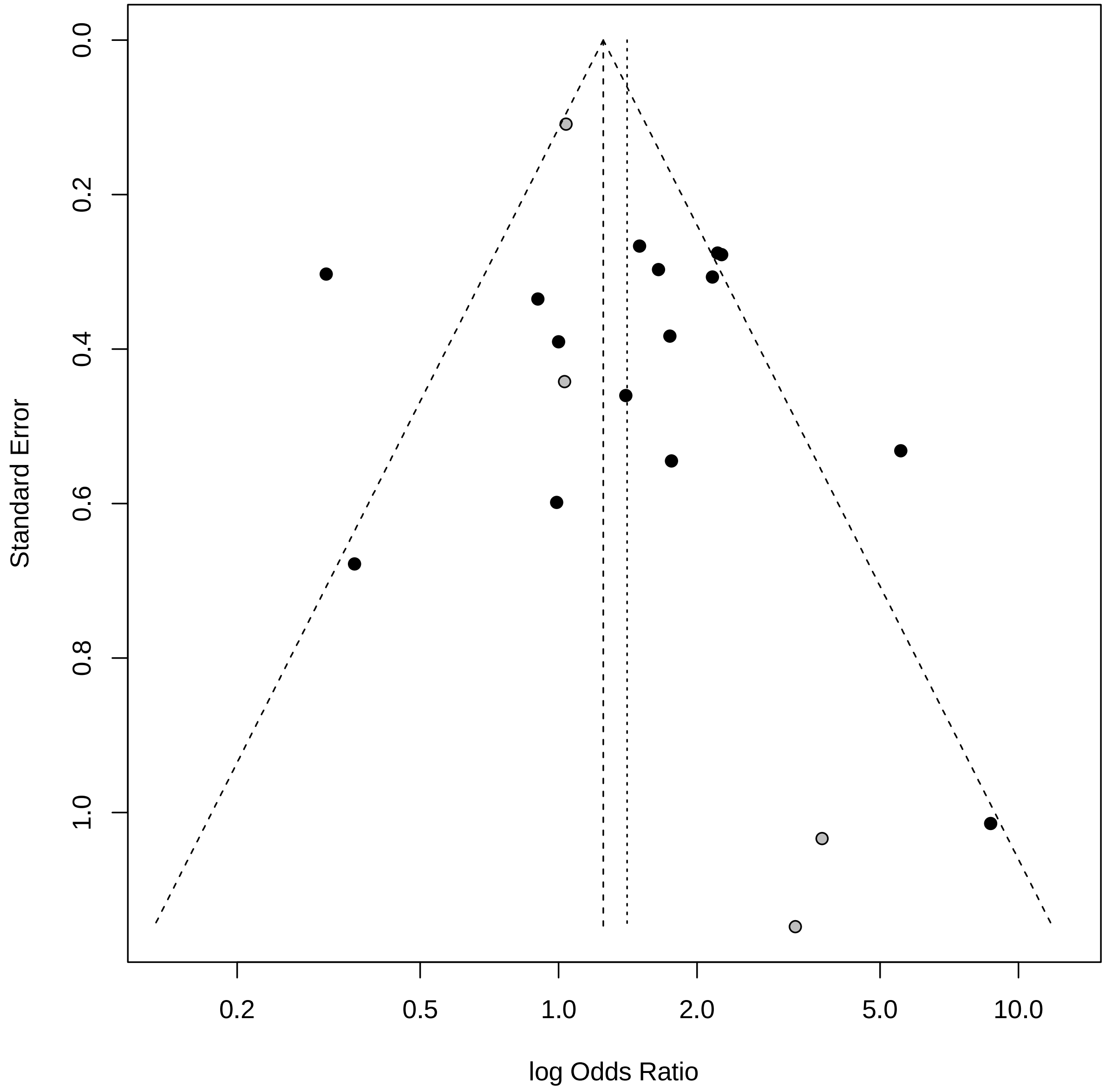

e-Figure 57

```
## [1] "p-value for Funnel-plot asymmetry (Eggers-test):  0.201"
```

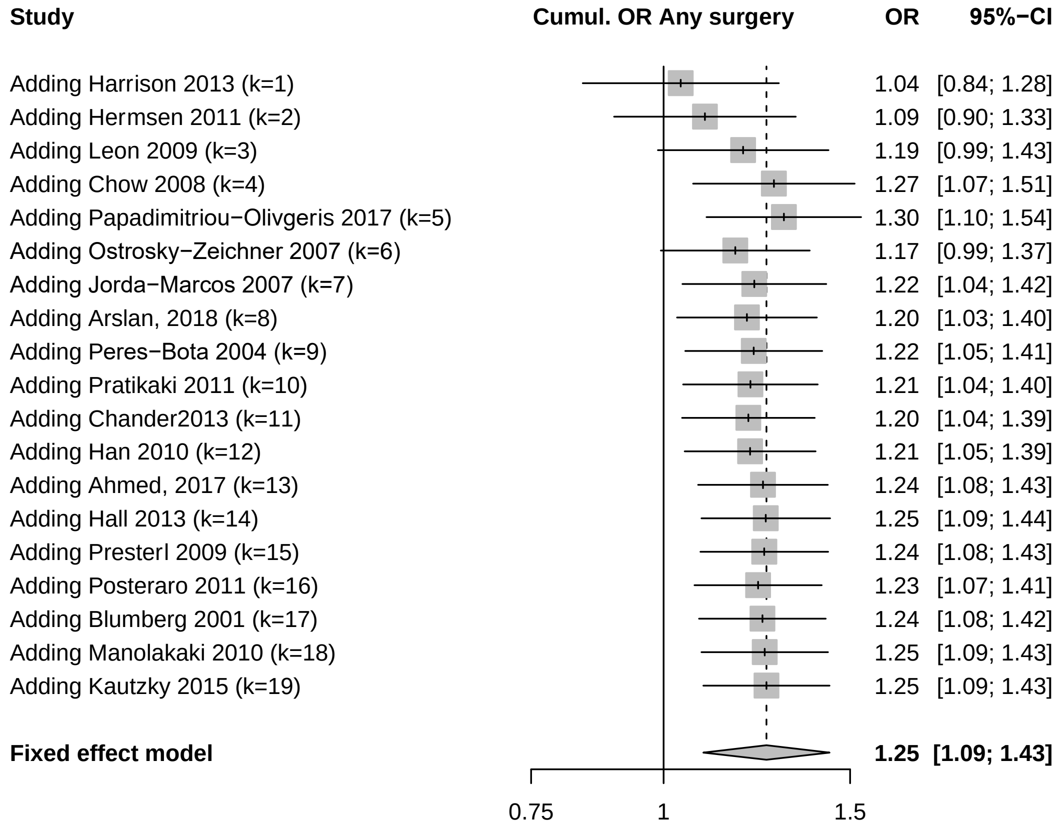
Study
Cumul. OR Any surgery
OR
95%−CI
Adding Harrison 2013 (k=1) 1.04 [0.84; 1.28]
Adding Hermsen 2011 (k=2) 1.09 [0.90; 1.33]
Adding Leon 2009 (k=3) 1.19 [0.99; 1.43]
Adding Chow 2008 (k=4) 1.27 [1.07; 1.51]
Adding Papadimitriou−Olivgeris 2017 (k=5) 1.30 [1.10; 1.54]
Adding Ostrosky−Zeichner 2007 (k=6) 1.17 [0.99; 1.37]
Adding Jorda−Marcos 2007 (k=7) 1.22 [1.04; 1.42]
Adding Arslan, 2018 (k=8) 1.20 [1.03; 1.40]
Adding Peres−Bota 2004 (k=9) 1.22 [1.05; 1.41]
Adding Pratikaki 2011 (k=10) 1.21 [1.04; 1.40]
Adding Chander2013 (k=11) 1.20 [1.04; 1.39]
Adding Han 2010 (k=12) 1.21 [1.05; 1.39]
Adding Ahmed, 2017 (k=13) 1.24 [1.08; 1.43]
Adding Hall 2013 (k=14) 1.25 [1.09; 1.44]
Adding Presterl 2009 (k=15) 1.24 [1.08; 1.43]
Adding Posteraro 2011 (k=16) 1.23 [1.07; 1.41]
Adding Blumberg 2001 (k=17) 1.24 [1.08; 1.42]
Adding Manolakaki 2010 (k=18) 1.25 [1.09; 1.43]
Adding Kautzky 2015 (k=19) 1.25 [1.09; 1.43]
Fixed effect model 1.25 [1.09; 1.43]
0.75
1
1.5

e-Figure 58

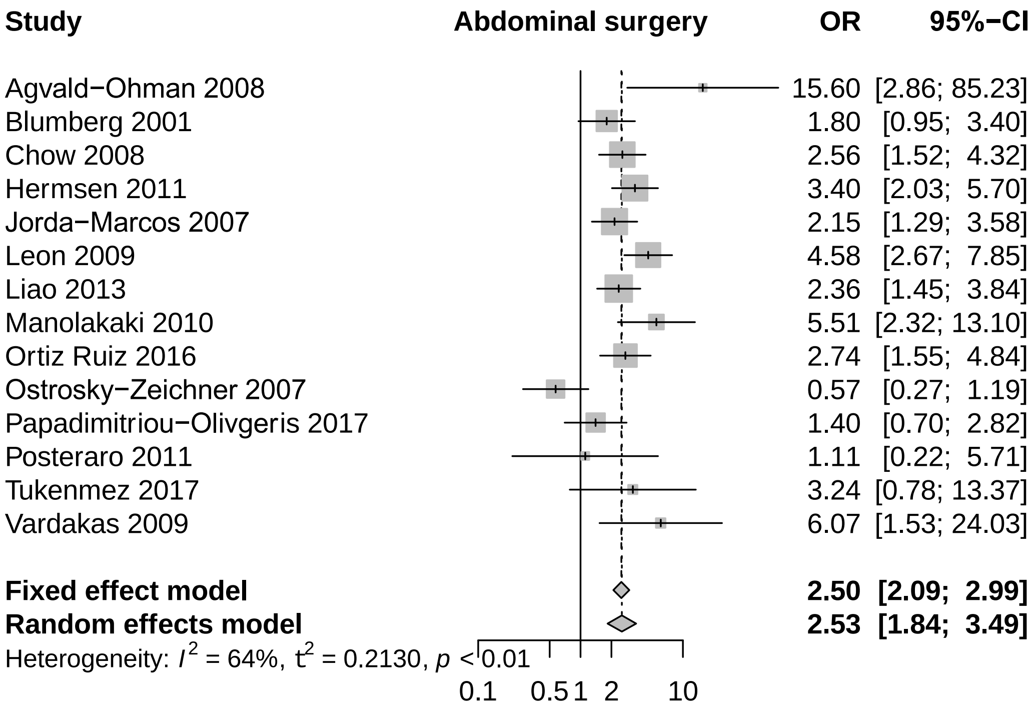

extensive gastro-abdominal surgery in Agvald-Ohman 2008
laparotomy in Manonalaki 2010
d1-3 used from Ostrosky-Zeichner 2007

e-Figure 59

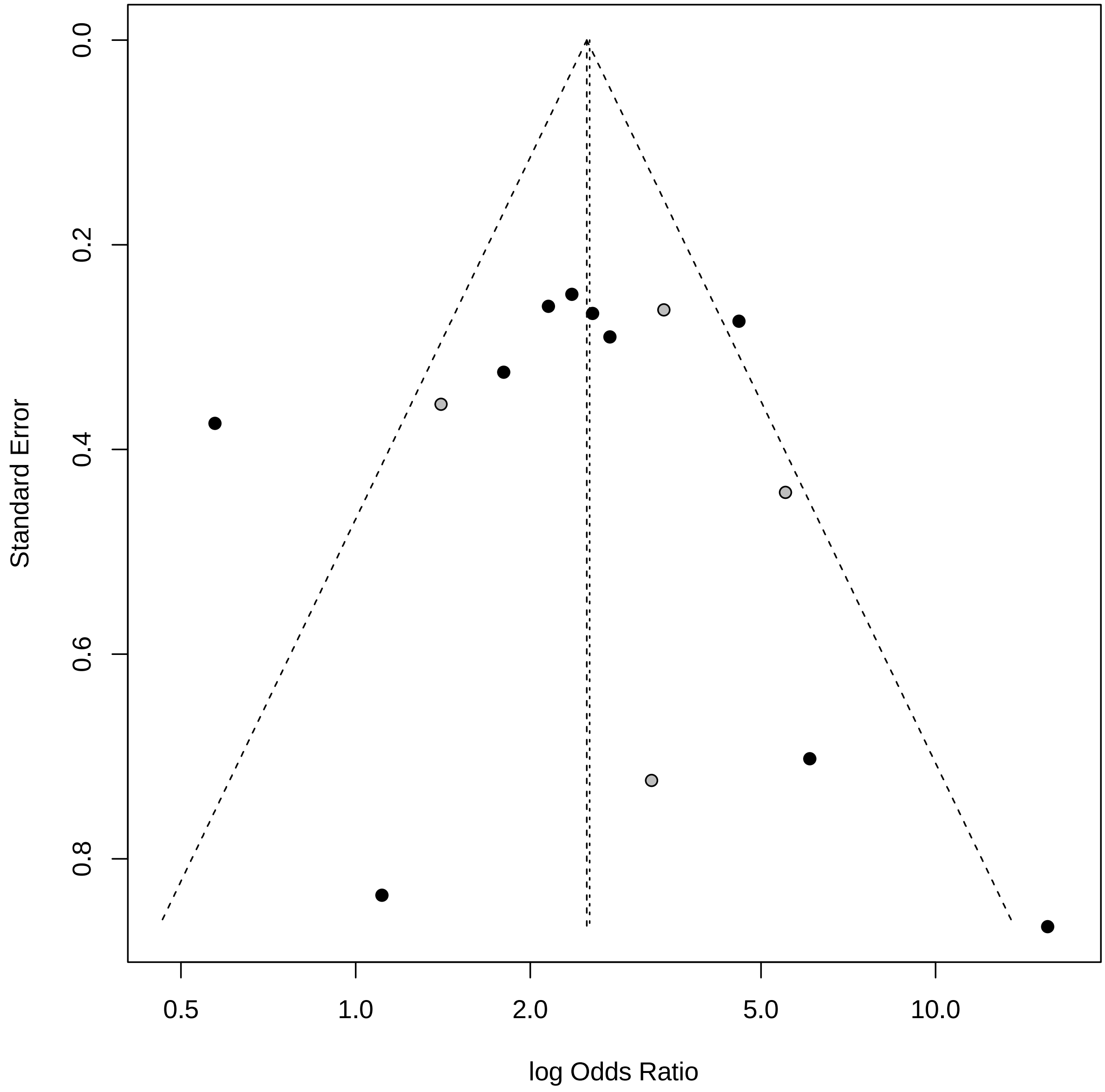

e-Figure 60

```
## [1] "p-value for Funnel-plot asymmetry (Eggers-test):  0.701"
```

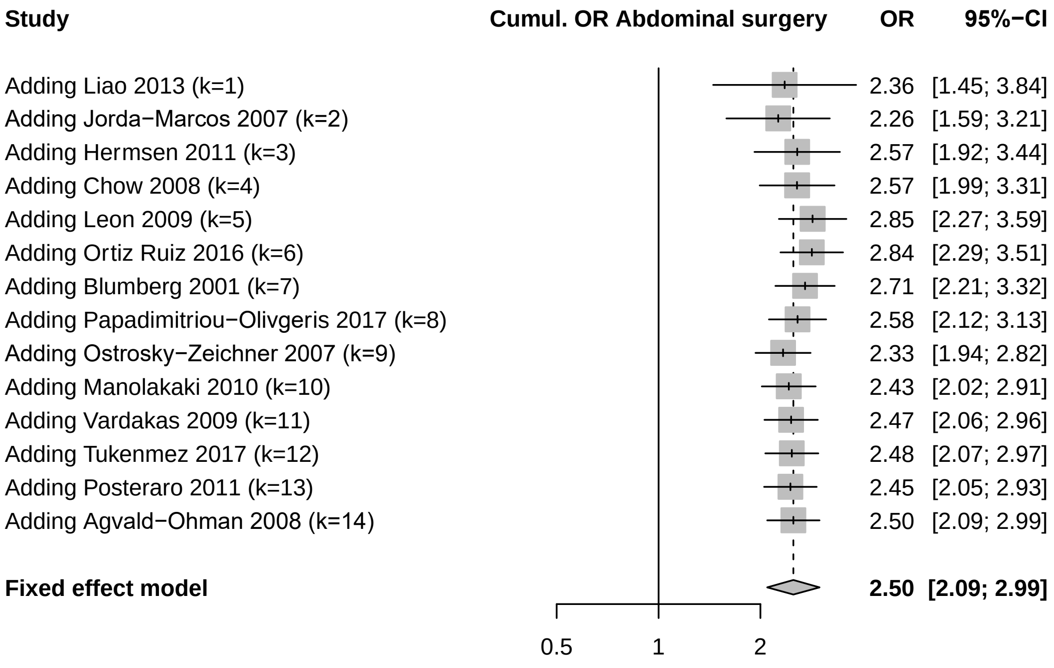
Study
Cumul. OR Abdominal surgery
OR
95%−CI
Adding Liao 2013 (k=1) 2.36 [1.45; 3.84]
Adding Jorda−Marcos 2007 (k=2) 2.26 [1.59; 3.21]
Adding Hermsen 2011 (k=3) 2.57 [1.92; 3.44]
Adding Chow 2008 (k=4) 2.57 [1.99; 3.31]
Adding Leon 2009 (k=5) 2.85 [2.27; 3.59]
Adding Ortiz Ruiz 2016 (k=6) 2.84 [2.29; 3.51]
Adding Blumberg 2001 (k=7) 2.71 [2.21; 3.32]
Adding Papadimitriou−Olivgeris 2017 (k=8) 2.58 [2.12; 3.13]
Adding Ostrosky−Zeichner 2007 (k=9) 2.33 [1.94; 2.82]
Adding Manolakaki 2010 (k=10) 2.43 [2.02; 2.91]
Adding Vardakas 2009 (k=11) 2.47 [2.06; 2.96]
Adding Tukenmez 2017 (k=12) 2.48 [2.07; 2.97]
Adding Posteraro 2011 (k=13) 2.45 [2.05; 2.93]
Adding Agvald−Ohman 2008 (k=14) 2.50 [2.09; 2.99]
Fixed effect model 2.50 [2.09; 2.99]
0.5
1
2

e-Figure 61

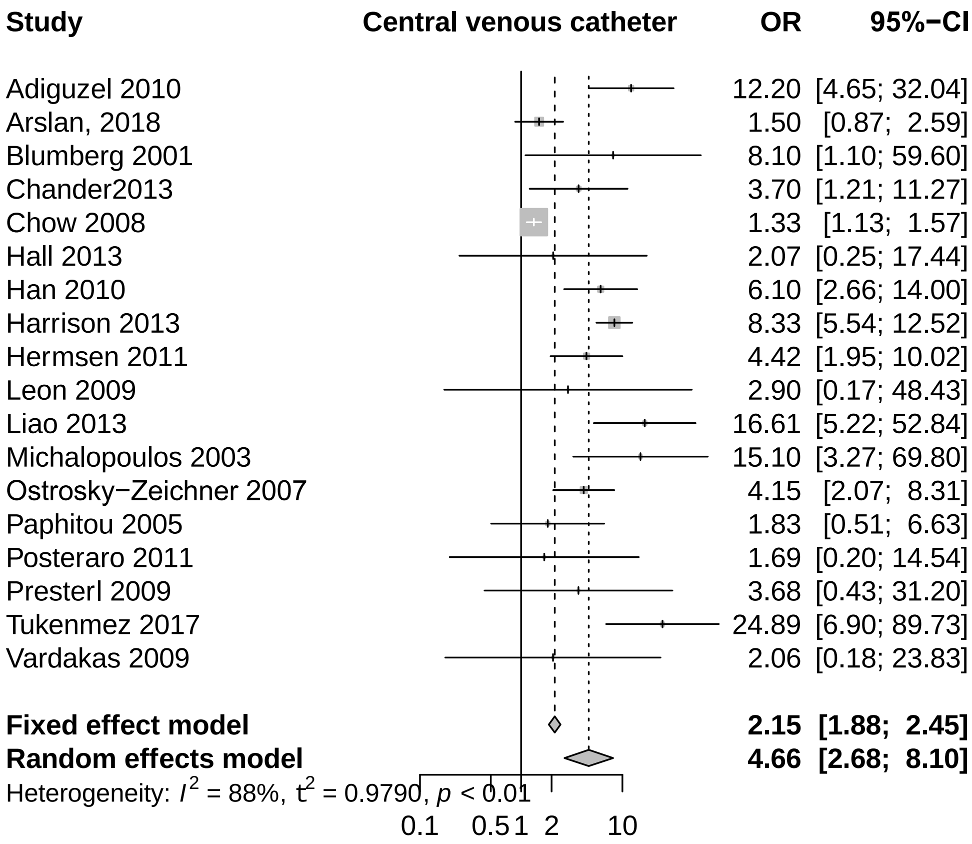

per day at risk in Chow 2008
≥72 hours in Michalopoulos 2003
d1-3 used from Ostrosky-Zeichner 2007

All patients reported to have a CVC by Kautzky 2015

e-Figure 62

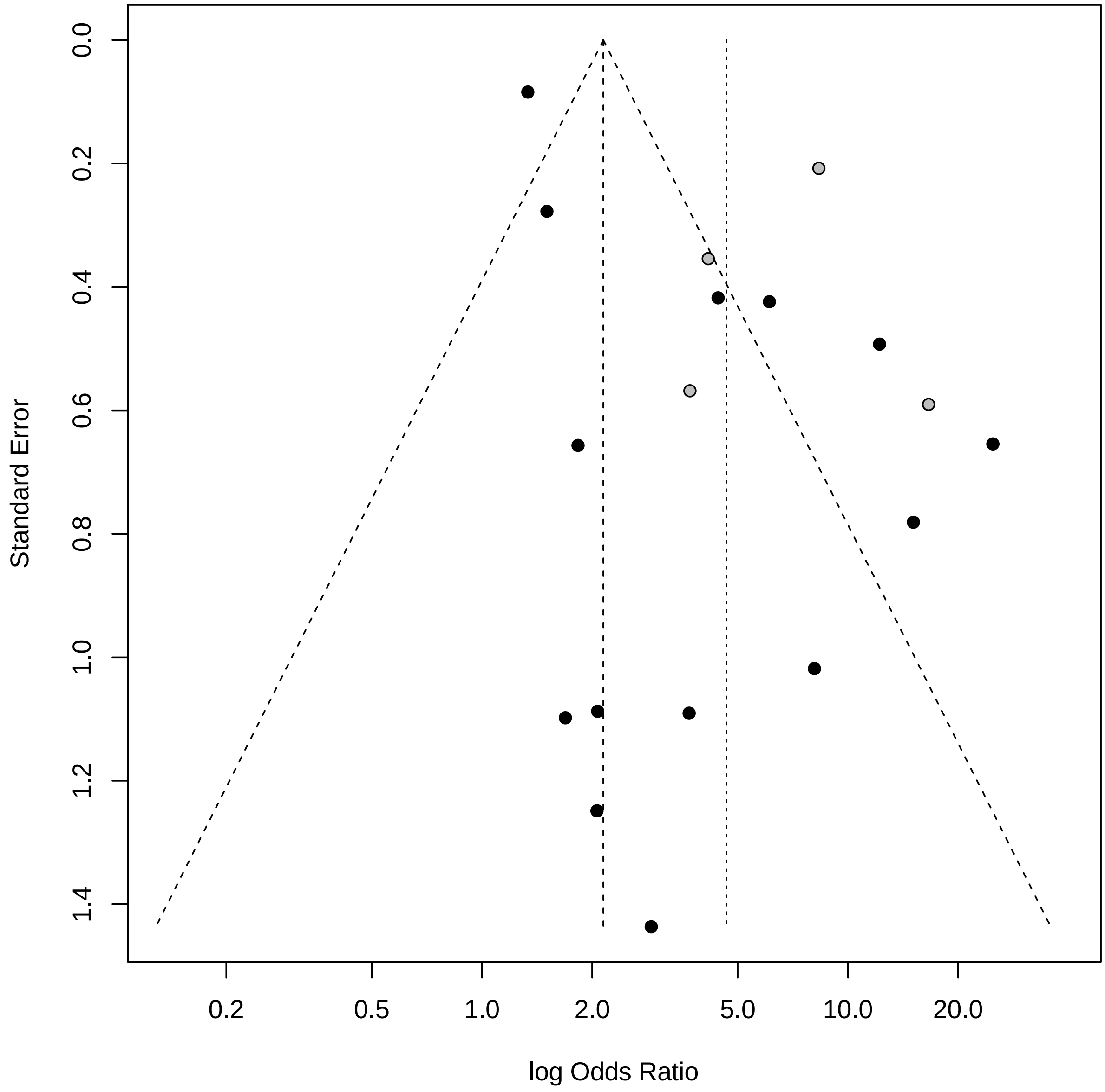

e-Figure 63

```
## [1] "p-value for Funnel-plot asymmetry (Eggers-test):  0.01"

Might be caused by the day at risk information with low OR from Chow
```

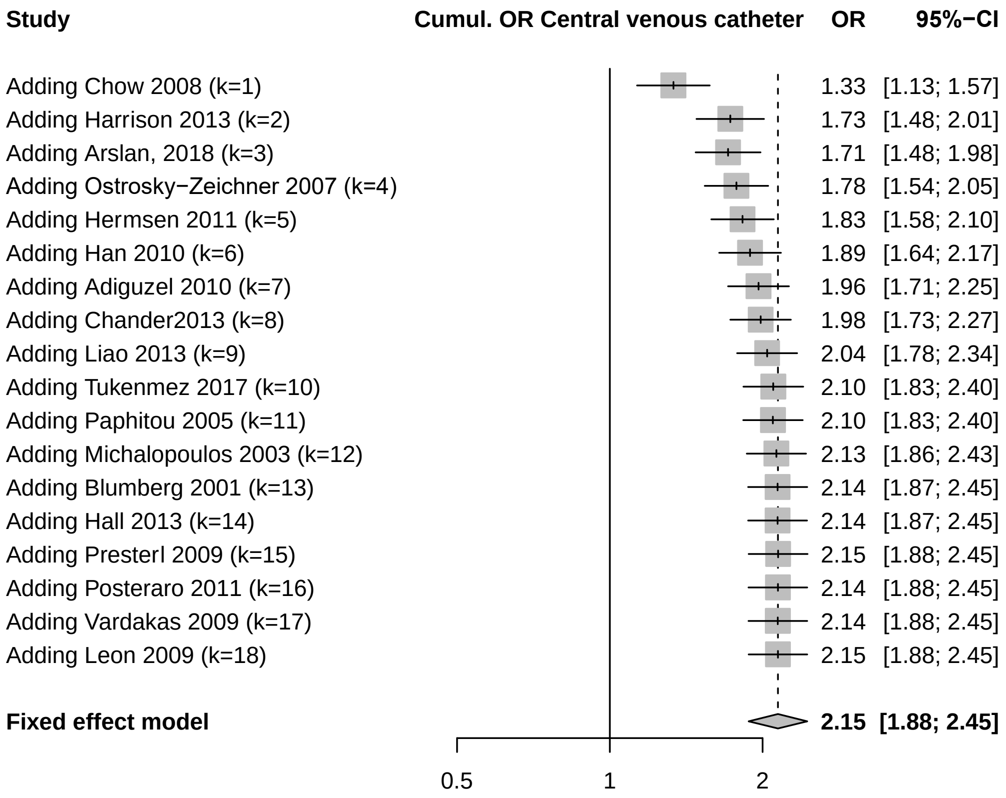
Study
Cumul. OR Central venous catheter
OR
95%−CI
Adding Chow 2008 (k=1) 1.33 [1.13; 1.57]
Adding Harrison 2013 (k=2) 1.73 [1.48; 2.01]
Adding Arslan, 2018 (k=3) 1.71 [1.48; 1.98]
Adding Ostrosky−Zeichner 2007 (k=4) 1.78 [1.54; 2.05]
Adding Hermsen 2011 (k=5) 1.83 [1.58; 2.10]
Adding Han 2010 (k=6) 1.89 [1.64; 2.17]
Adding Adiguzel 2010 (k=7) 1.96 [1.71; 2.25]
Adding Chander2013 (k=8) 1.98 [1.73; 2.27]
Adding Liao 2013 (k=9) 2.04 [1.78; 2.34]
Adding Tukenmez 2017 (k=10) 2.10 [1.83; 2.40]
Adding Paphitou 2005 (k=11) 2.10 [1.83; 2.40]
Adding Michalopoulos 2003 (k=12) 2.13 [1.86; 2.43]
Adding Blumberg 2001 (k=13) 2.14 [1.87; 2.45]
Adding Hall 2013 (k=14) 2.14 [1.87; 2.45]
Adding Presterl 2009 (k=15) 2.15 [1.88; 2.45]
Adding Posteraro 2011 (k=16) 2.14 [1.88; 2.45]
Adding Vardakas 2009 (k=17) 2.14 [1.88; 2.45]
Adding Leon 2009 (k=18) 2.15 [1.88; 2.45]
Fixed effect model 2.15 [1.88; 2.45]
0.5
1
2

e-Figure 64

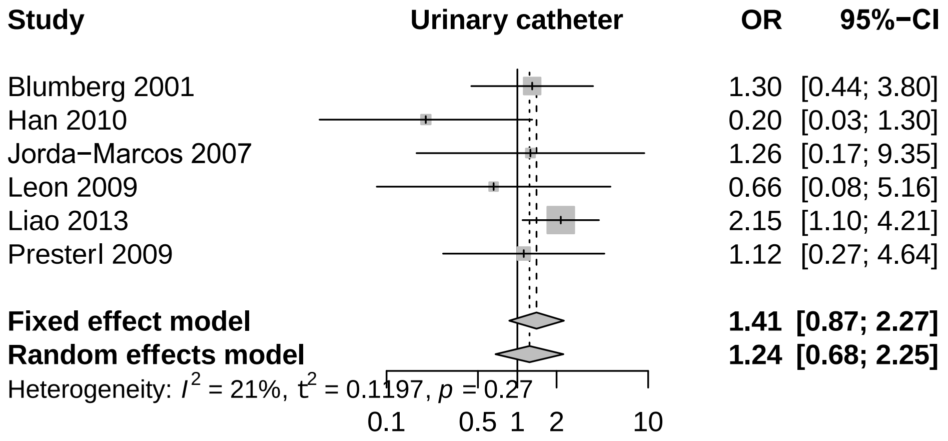
Study
Urinary catheter
OR
95%−CI
Blumberg 2001
Han 2010
Jorda−Marcos 2007
Leon 2009
Liao 2013
Presterl 2009
1.30 [0.44; 3.80]
0.20 [0.03; 1.30]
1.26 [0.17; 9.35]
0.66 [0.08; 5.16]
2.15 [1.10; 4.21]
1.12 [0.27; 4.64]
Fixed effect model
Random effects model
1.41 [0.87; 2.27]
1.24 [0.68; 2.25]
Heterogeneity: I^2 = 21%, τ^2 = 0.1197, p = 0.27
0.1
0.5
1
2
10

e-Figure 65

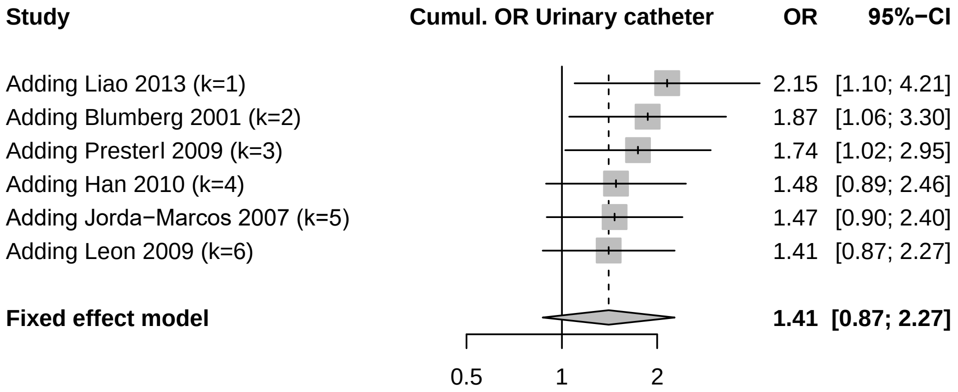
Study
Cumul. OR Urinary catheter
OR
95%−CI
Adding Liao 2013 (k=1) 2.15 [1.10; 4.21]
Adding Blumberg 2001 (k=2) 1.87 [1.06; 3.30]
Adding Presterl 2009 (k=3) 1.74 [1.02; 2.95]
Adding Han 2010 (k=4) 1.48 [0.89; 2.46]
Adding Jorda−Marcos 2007 (k=5) 1.47 [0.90; 2.40]
Adding Leon 2009 (k=6) 1.41 [0.87; 2.27]
Fixed effect model 1.41 [0.87; 2.27]
0.5
1
2

e-Figure 66

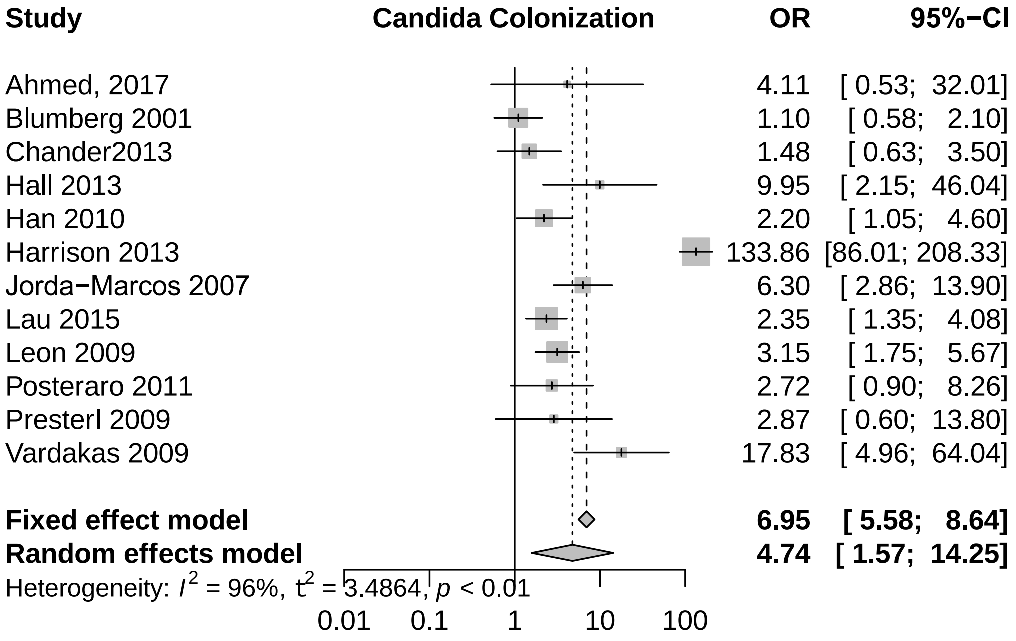

multifocal in Ahmed 2017
urine+rectal used from Blumberg 2001
urine in Chander 2013
colonization identified in unit from Harisson 2013
any site colonized, timepoint 1 used from Lau 2015

e-Figure 67

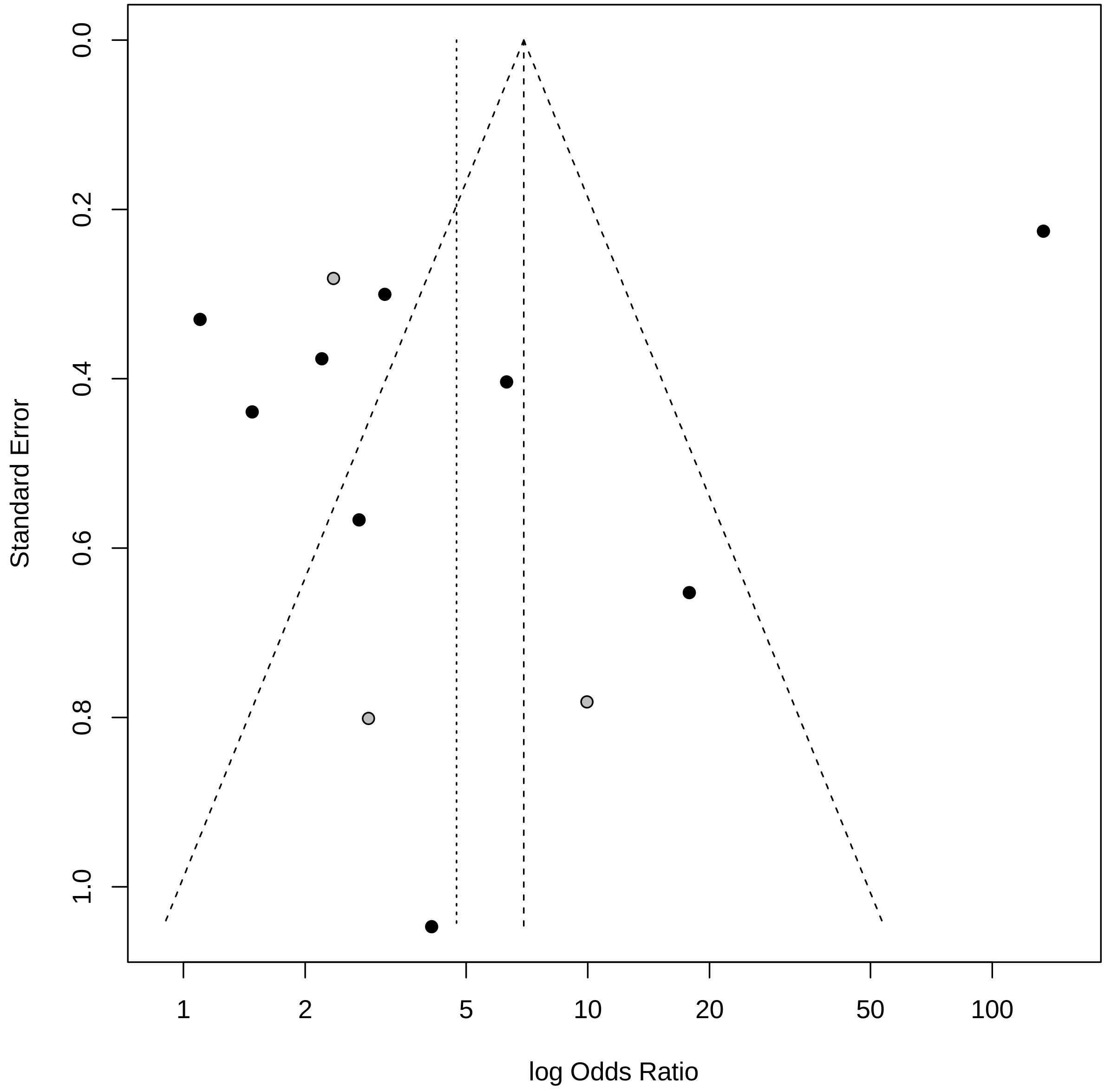

e-Figure 68

```
## [1] "p-value for Funnel-plot asymmetry (Eggers-test):  0.3"
```

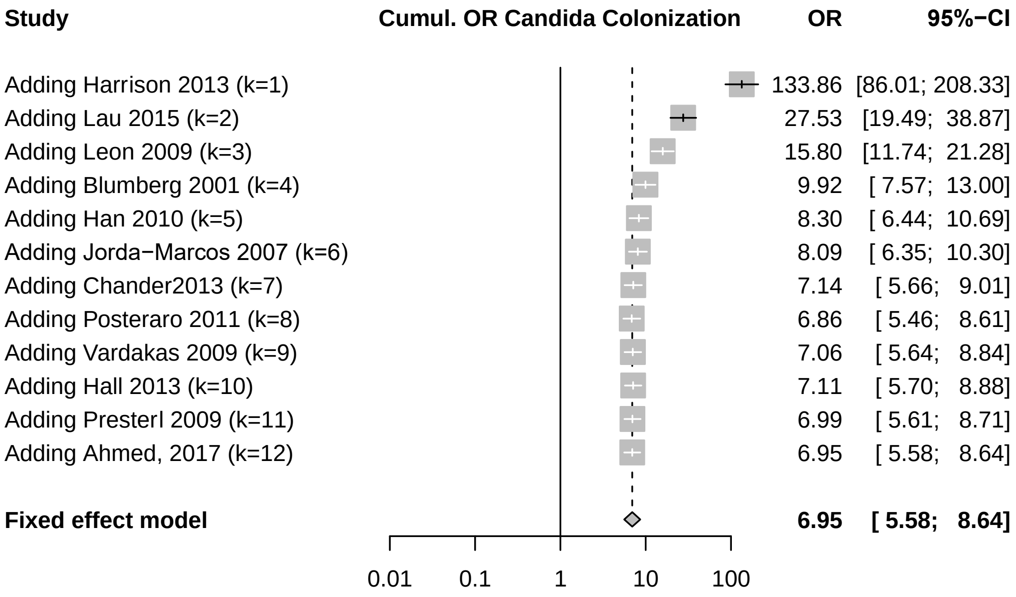
Study
Cumul. OR Candida Colonization
OR
95%−CI
Adding Harrison 2013 (k=1) 133.86 [86.01; 208.33]
Adding Lau 2015 (k=2) 27.53 [19.49; 38.87]
Adding Leon 2009 (k=3) 15.80 [11.74; 21.28]
Adding Blumberg 2001 (k=4) 9.92 [7.57; 13.00]
Adding Han 2010 (k=5) 8.30 [6.44; 10.69]
Adding Jorda−Marcos 2007 (k=6) 8.09 [6.35; 10.30]
Adding Chander2013 (k=7) 7.14 [5.66; 9.01]
Adding Posteraro 2011 (k=8) 6.86 [5.46; 8.61]
Adding Vardakas 2009 (k=9) 7.06 [5.64; 8.84]
Adding Hall 2013 (k=10) 7.11 [5.70; 8.88]
Adding Presterl 2009 (k=11) 6.99 [5.61; 8.71]
Adding Ahmed, 2017 (k=12) 6.95 [5.58; 8.64]
Fixed effect model 6.95 [5.58; 8.64]
0.01
0.1
1
10
100

e-Figure 69

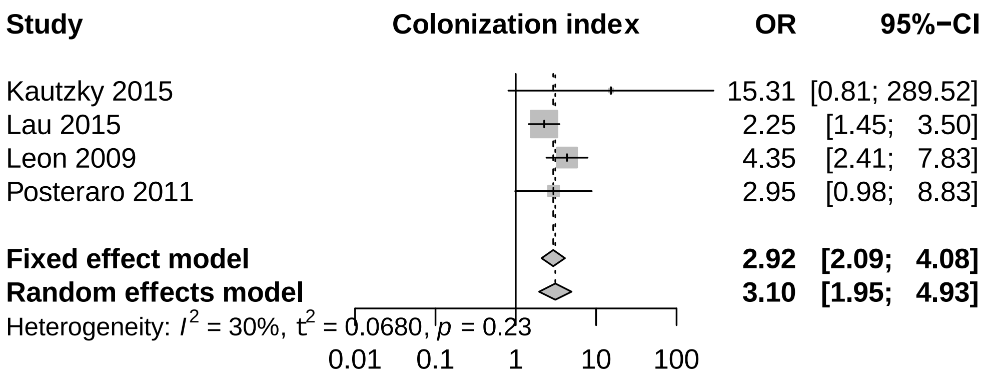

CCI ≥0.5 in Kautzky 2015, Leon 2009 & Posteraro 2011
CCI >0.5 at timepoint 1 taken from Lau 2015

0.6±0.3 points vs. 0.4±0.3 (p=0.04) in Ahmed 2017

e-Figure 70

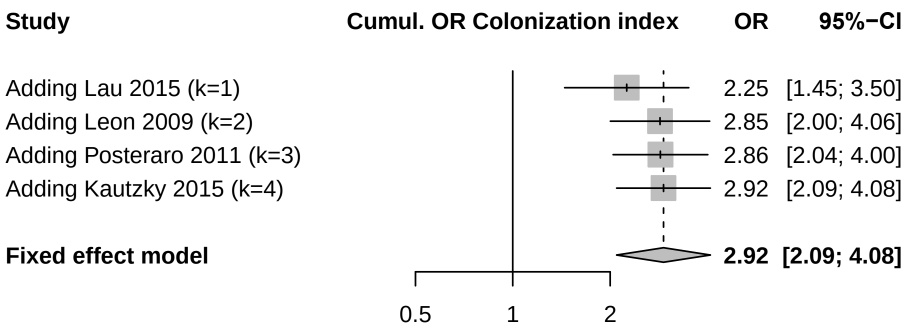
Study
Cumul. OR Colonization index
OR
95%−CI
Adding Lau 2015 (k=1)
2.25
[1.45; 3.50]
Adding Leon 2009 (k=2)
2.85
[2.00; 4.06]
Adding Posteraro 2011 (k=3)
2.86
[2.04; 4.00]
Adding Kautzky 2015 (k=4)
2.92
[2.09; 4.08]
Fixed effect model
2.92
[2.09; 4.08]
0.5
1
2

e-Figure 71

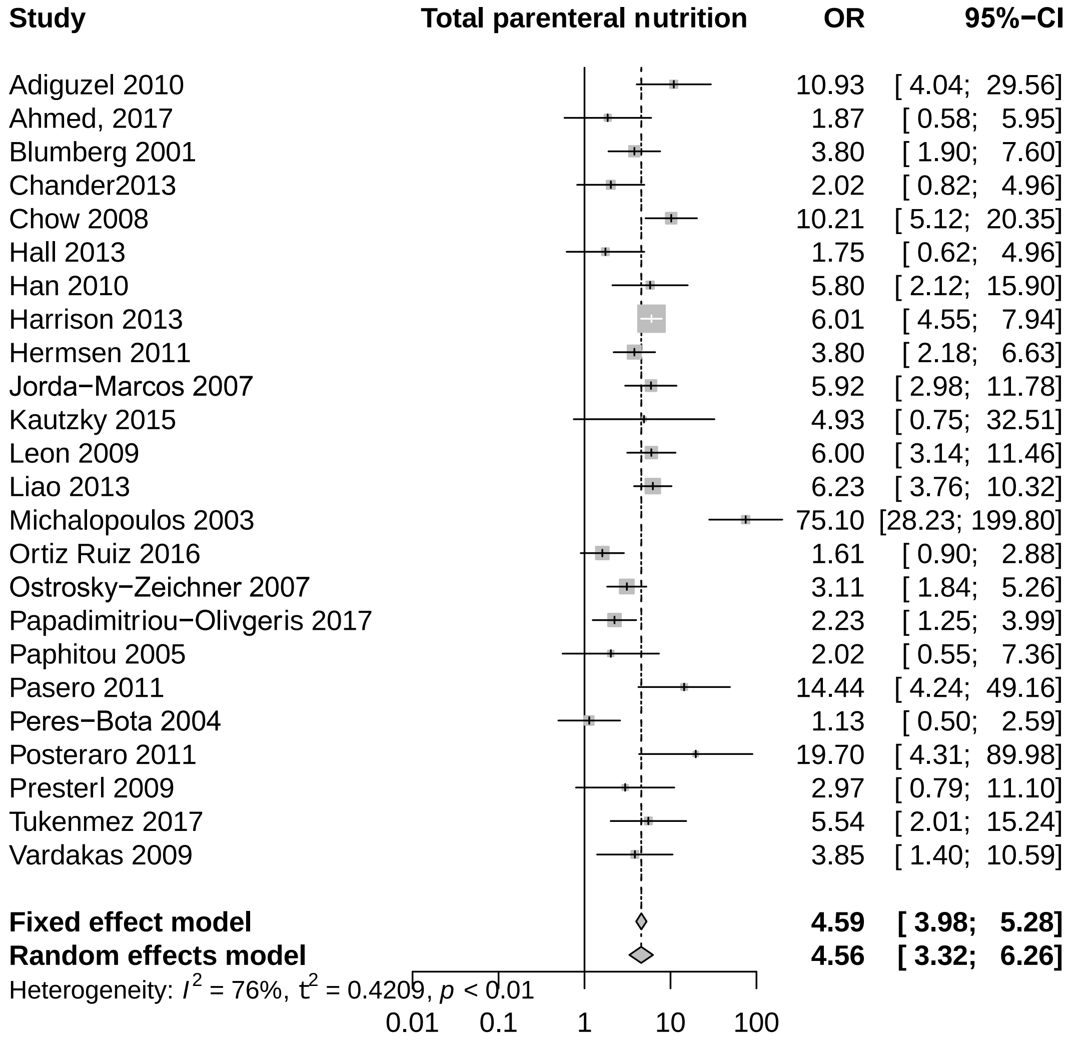

per day at risk in Chow 2008

D 1-3 taken form Ostrosky-Zeichner 2017

e-Figure 72

12±17 days vs. 1±4 (p=0.004) in Manonalaki 2010

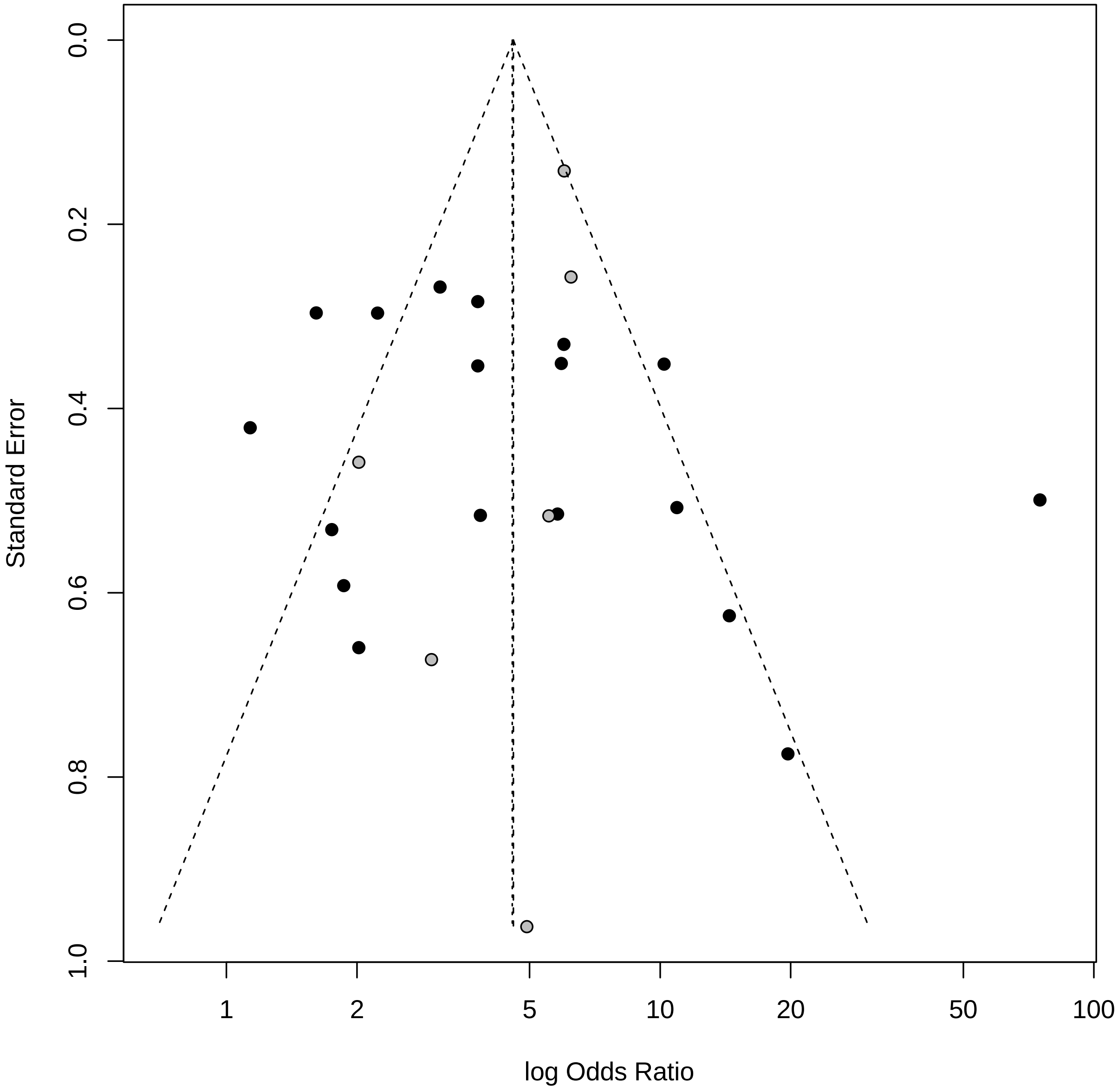

e-Figure 73

```
## [1] "p-value for Funnel-plot asymmetry (Eggers-test):  0.921"
```

Study	Cumul. OR Total parenteral nutrition	OR	95%−CI
Adding Harrison 2013 (k=1)		6.01	[4.55; 7.94]
Adding Liao 2013 (k=2)		6.06	[4.75; 7.73]
Adding Ostrosky−Zeichner 2007 (k=3)		5.39	[4.32; 6.72]
Adding Hermsen 2011 (k=4)		5.14	[4.18; 6.31]
Adding Ortiz Ruiz 2016 (k=5)		4.51	[3.72; 5.48]
Adding Papadimitriou−Olivgeris 2017 (k=6)		4.21	[3.50; 5.06]
Adding Leon 2009 (k=7)		4.32	[3.62; 5.15]
Adding Jorda−Marcos 2007 (k=8)		4.40	[3.71; 5.23]
Adding Chow 2008 (k=9)		4.62	[3.92; 5.46]
Adding Blumberg 2001 (k=10)		4.58	[3.89; 5.38]
Adding Peres−Bota 2004 (k=11)		4.35	[3.71; 5.09]
Adding Chander2013 (k=12)		4.25	[3.63; 4.96]
Adding Michalopoulos 2003 (k=13)		4.56	[3.91; 5.32]
Adding Adiguzel 2010 (k=14)		4.66	[4.00; 5.42]
Adding Han 2010 (k=15)		4.68	[4.02; 5.44]
Adding Vardakas 2009 (k=16)		4.66	[4.01; 5.41]
Adding Tukenmez 2017 (k=17)		4.68	[4.03; 5.42]
Adding Hall 2013 (k=18)		4.59	[3.96; 5.31]
Adding Ahmed, 2017 (k=19)		4.52	[3.91; 5.23]
Adding Pasero 2011 (k=20)		4.60	[3.98; 5.31]
Adding Paphitou 2005 (k=21)		4.55	[3.94; 5.25]
Adding Presterl 2009 (k=22)		4.53	[3.93; 5.22]
Adding Posteraro 2011 (k=23)		4.58	[3.98; 5.28]
Adding Kautzky 2015 (k=24)		4.59	[3.98; 5.28]
**Fixed effect model**		**4.59**	**[3.98; 5.28]**

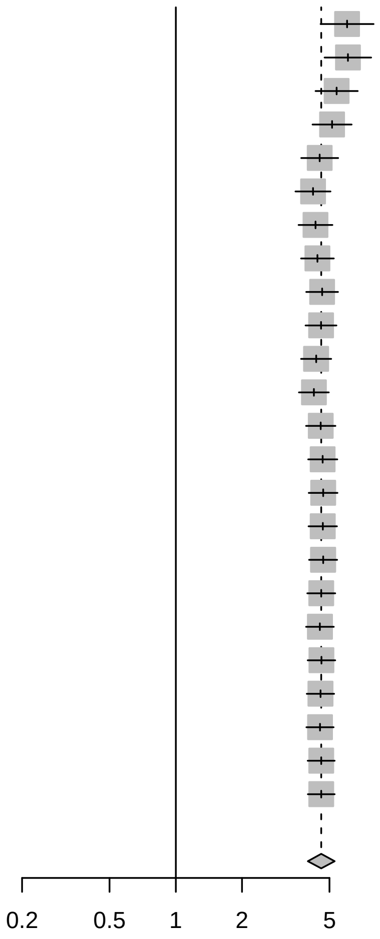

e-Figure 74

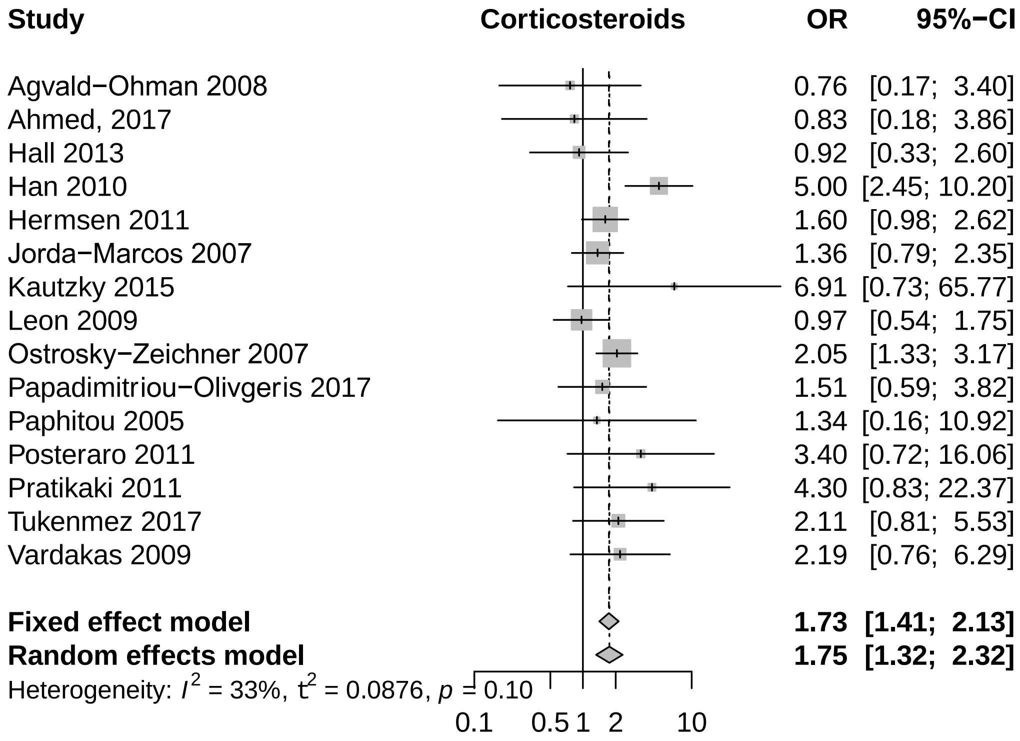
Study
Corticosteroids
OR
95%−CI
Agvald−Ohman 2008 0.76 [0.17; 3.40]
Ahmed, 2017 0.83 [0.18; 3.86]
Hall 2013 0.92 [0.33; 2.60]
Han 2010 5.00 [2.45; 10.20]
Hermsen 2011 1.60 [0.98; 2.62]
Jorda−Marcos 2007 1.36 [0.79; 2.35]
Kautzky 2015 6.91 [0.73; 65.77]
Leon 2009 0.97 [0.54; 1.75]
Ostrosky−Zeichner 2007 2.05 [1.33; 3.17]
Papadimitriou−Olivgeris 2017 1.51 [0.59; 3.82]
Paphitou 2005 1.34 [0.16; 10.92]
Posteraro 2011 3.40 [0.72; 16.06]
Pratikaki 2011 4.30 [0.83; 22.37]
Tukenmez 2017 2.11 [0.81; 5.53]
Vardakas 2009 2.19 [0.76; 6.29]
Fixed effect model 1.73 [1.41; 2.13]
Random effects model 1.75 [1.32; 2.32]
Heterogeneity: I2 = 33%, τ2 = 0.0876, p = 0.10
0.1
0.5
1
2
10

e-Figure 75

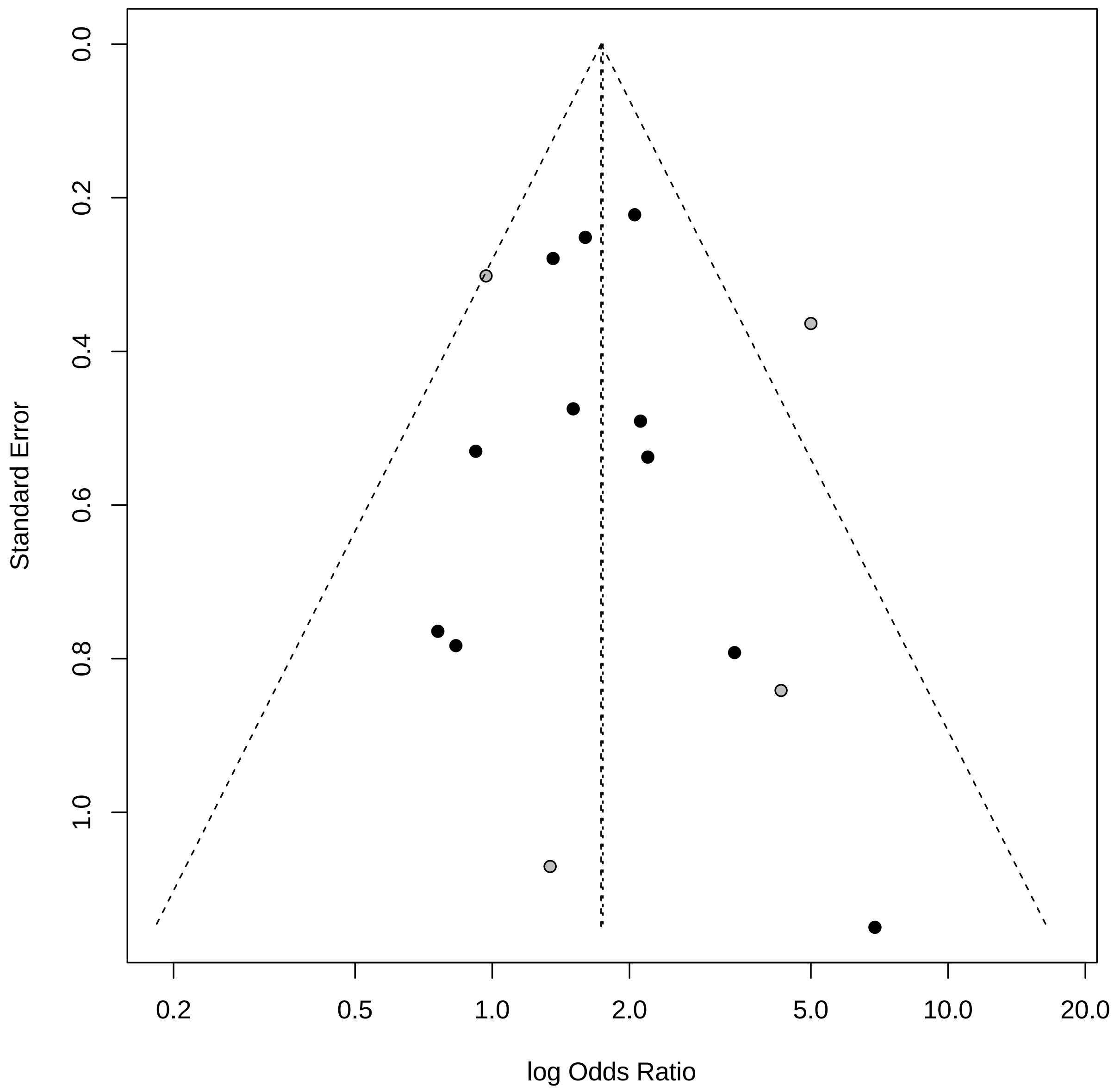

e-Figure 76

```
## [1] "p-value for Funnel-plot asymmetry (Eggers-test):  0.73"
```

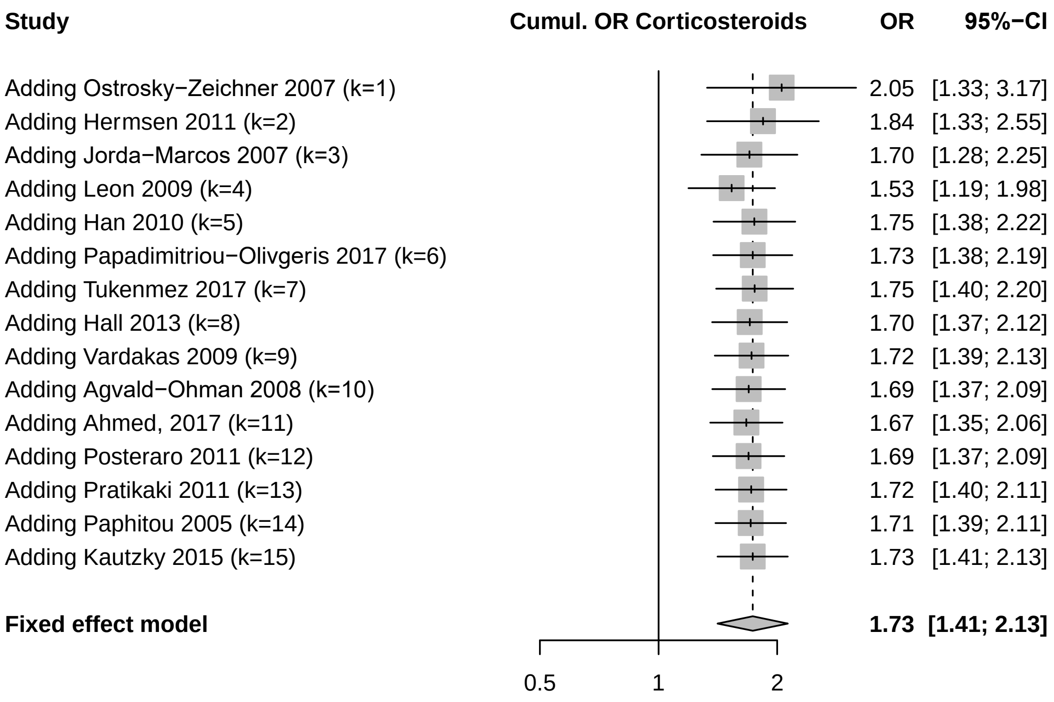
Study
Cumul. OR Corticosteroids
OR
95%−CI
Adding Ostrosky−Zeichner 2007 (k=1) 2.05 [1.33; 3.17]
Adding Hermsen 2011 (k=2) 1.84 [1.33; 2.55]
Adding Jorda−Marcos 2007 (k=3) 1.70 [1.28; 2.25]
Adding Leon 2009 (k=4) 1.53 [1.19; 1.98]
Adding Han 2010 (k=5) 1.75 [1.38; 2.22]
Adding Papadimitriou−Olivgeris 2017 (k=6) 1.73 [1.38; 2.19]
Adding Tukenmez 2017 (k=7) 1.75 [1.40; 2.20]
Adding Hall 2013 (k=8) 1.70 [1.37; 2.12]
Adding Vardakas 2009 (k=9) 1.72 [1.39; 2.13]
Adding Agvald−Ohman 2008 (k=10) 1.69 [1.37; 2.09]
Adding Ahmed, 2017 (k=11) 1.67 [1.35; 2.06]
Adding Posteraro 2011 (k=12) 1.69 [1.37; 2.09]
Adding Pratikaki 2011 (k=13) 1.72 [1.40; 2.11]
Adding Paphitou 2005 (k=14) 1.71 [1.39; 2.11]
Adding Kautzky 2015 (k=15) 1.73 [1.41; 2.13]
Fixed effect model 1.73 [1.41; 2.13]
0.5
1
2

e-Figure 77

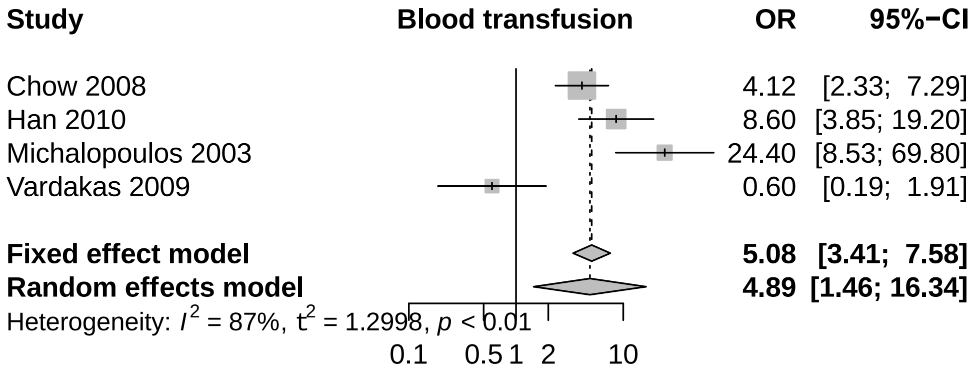

per mean RBC/day in Chow 2008

12±15 vs. 3±5 (p=0.01) RBC transfused 24-hours on admission (sic) in Manonalaki 2010

e-Figure 78

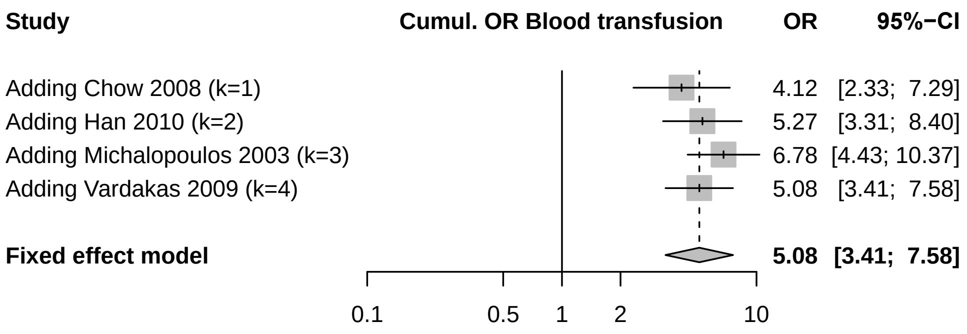
Study
Cumul. OR Blood transfusion
OR
95%−CI
Adding Chow 2008 (k=1)
4.12 [2.33; 7.29]
Adding Han 2010 (k=2)
5.27 [3.31; 8.40]
Adding Michalopoulos 2003 (k=3)
6.78 [4.43; 10.37]
Adding Vardakas 2009 (k=4)
5.08 [3.41; 7.58]
Fixed effect model
5.08 [3.41; 7.58]
0.1
0.5
1
2
10

e-Figure 79

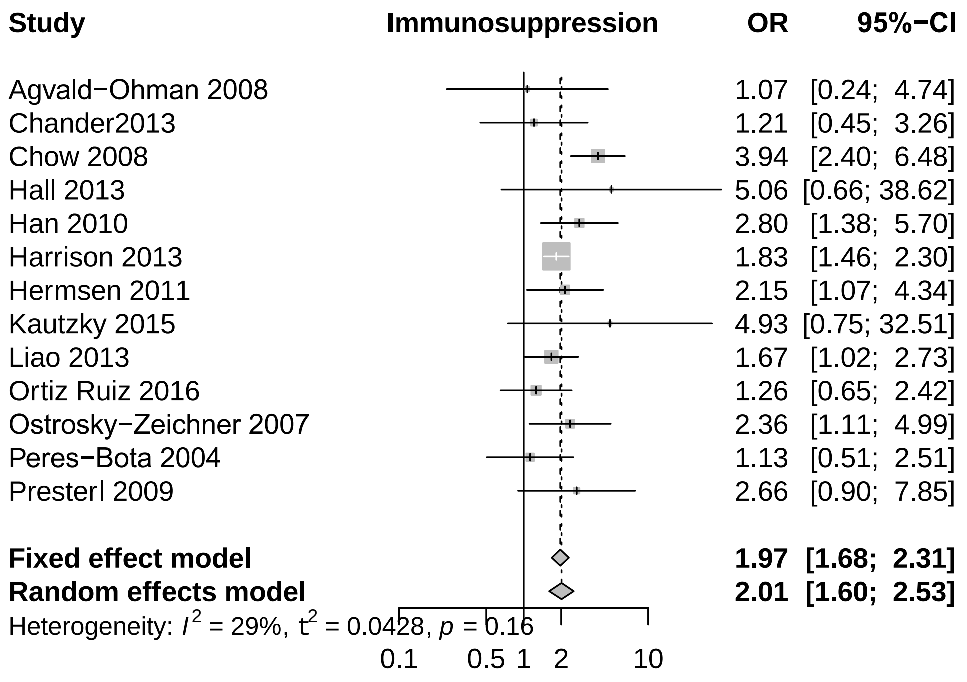
Study
Immunosuppression
OR
95%−CI
Agvald−Ohman 2008 1.07 [0.24; 4.74]
Chander2013 1.21 [0.45; 3.26]
Chow 2008 3.94 [2.40; 6.48]
Hall 2013 5.06 [0.66; 38.62]
Han 2010 2.80 [1.38; 5.70]
Harrison 2013 1.83 [1.46; 2.30]
Hermsen 2011 2.15 [1.07; 4.34]
Kautzky 2015 4.93 [0.75; 32.51]
Liao 2013 1.67 [1.02; 2.73]
Ortiz Ruiz 2016 1.26 [0.65; 2.42]
Ostrosky−Zeichner 2007 2.36 [1.11; 4.99]
Peres−Bota 2004 1.13 [0.51; 2.51]
Presterl 2009 2.66 [0.90; 7.85]
Fixed effect model 1.97 [1.68; 2.31]
Random effects model 2.01 [1.60; 2.53]
Heterogeneity: I^2 = 29%, τ^2 = 0.0428, p = 0.16
0.1
0.5
1
2
10

e-Figure 80

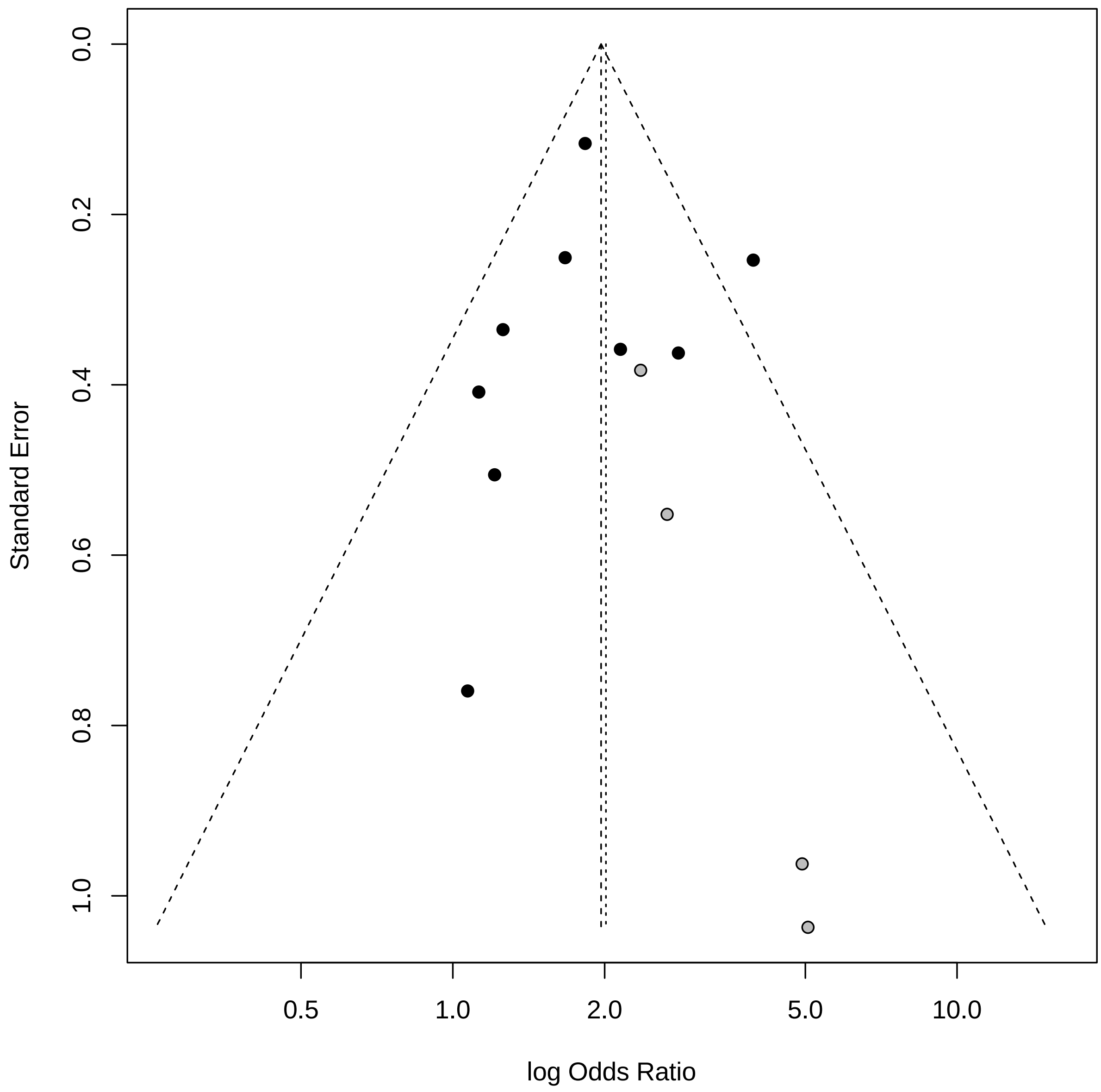

e-Figure 81

```
## [1] "p-value for Funnel-plot asymmetry (Eggers-test):  0.663"
```

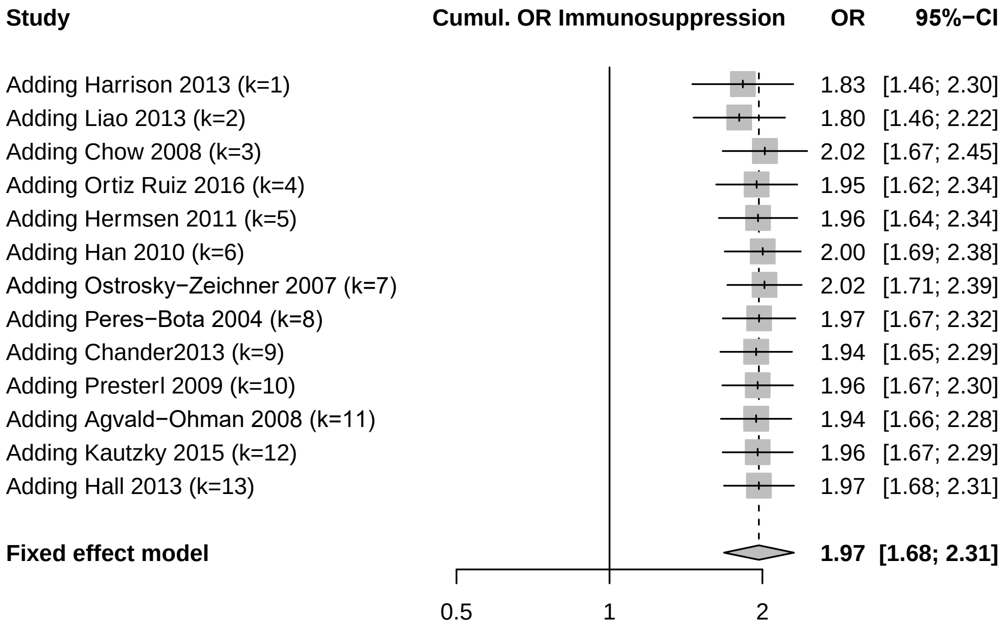
Study
Cumul. OR Immunosuppression
OR
95%−CI
Adding Harrison 2013 (k=1) 1.83 [1.46; 2.30]
Adding Liao 2013 (k=2) 1.80 [1.46; 2.22]
Adding Chow 2008 (k=3) 2.02 [1.67; 2.45]
Adding Ortiz Ruiz 2016 (k=4) 1.95 [1.62; 2.34]
Adding Hermsen 2011 (k=5) 1.96 [1.64; 2.34]
Adding Han 2010 (k=6) 2.00 [1.69; 2.38]
Adding Ostrosky−Zeichner 2007 (k=7) 2.02 [1.71; 2.39]
Adding Peres−Bota 2004 (k=8) 1.97 [1.67; 2.32]
Adding Chander2013 (k=9) 1.94 [1.65; 2.29]
Adding Presterl 2009 (k=10) 1.96 [1.67; 2.30]
Adding Agvald−Ohman 2008 (k=11) 1.94 [1.66; 2.28]
Adding Kautzky 2015 (k=12) 1.96 [1.67; 2.29]
Adding Hall 2013 (k=13) 1.97 [1.68; 2.31]
Fixed effect model 1.97 [1.68; 2.31]
0.5
1
2

e-Figure 82

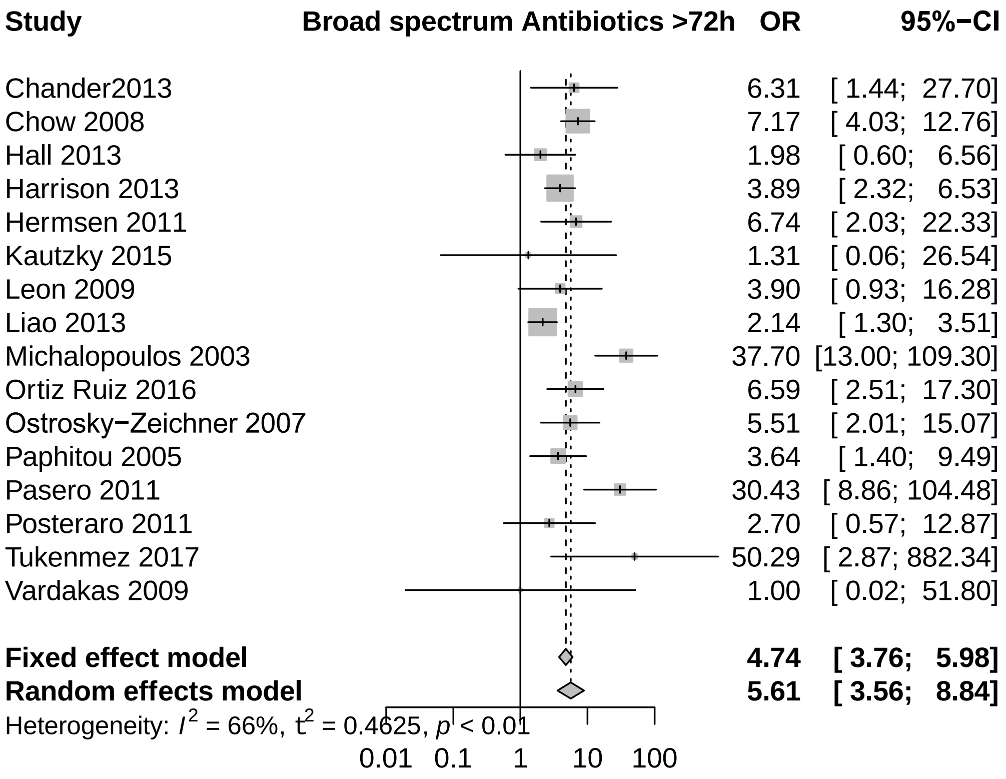

Pip/Taz & any antibiotics pooled in Chow 2008
unspecified antibiotics in Hall 2013, Harrison 2013 & Kautzky 2015
broad spectrum antibiotics in Leon 2009, Liao 2013, Ortiz Ruiz 2016, Ostrosky-Zeichner 2007, Posteraro 2011 & Tukenmenez 2017
day 1-3 taken from Ostrosky-Zeichner 2007
≥4days in Liao 2013
≥2 antibiotics >72 h in Michalopoulos 2003
>2 antibiotics >72 h in Pasero 2011
e-Figure 83 all patients in Vardakas 2009

3.9±2.5 vs. 2.3±1.3 (p=0.005) antibiotics until positive culture for fungus in Manonalaki 2010

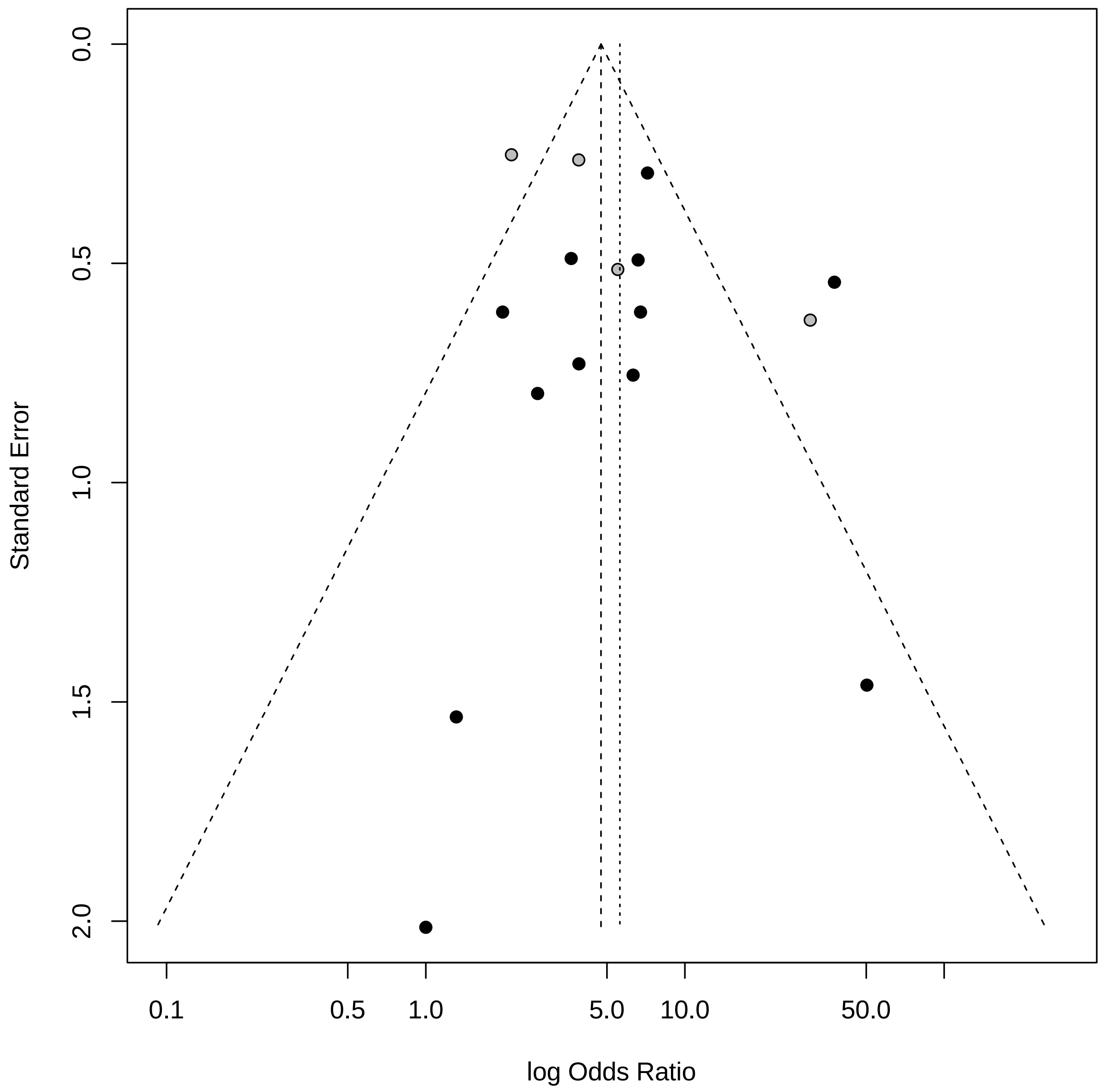

e-Figure 84

```
## [1] "p-value for Funnel-plot asymmetry (Eggers-test):  0.338"
```

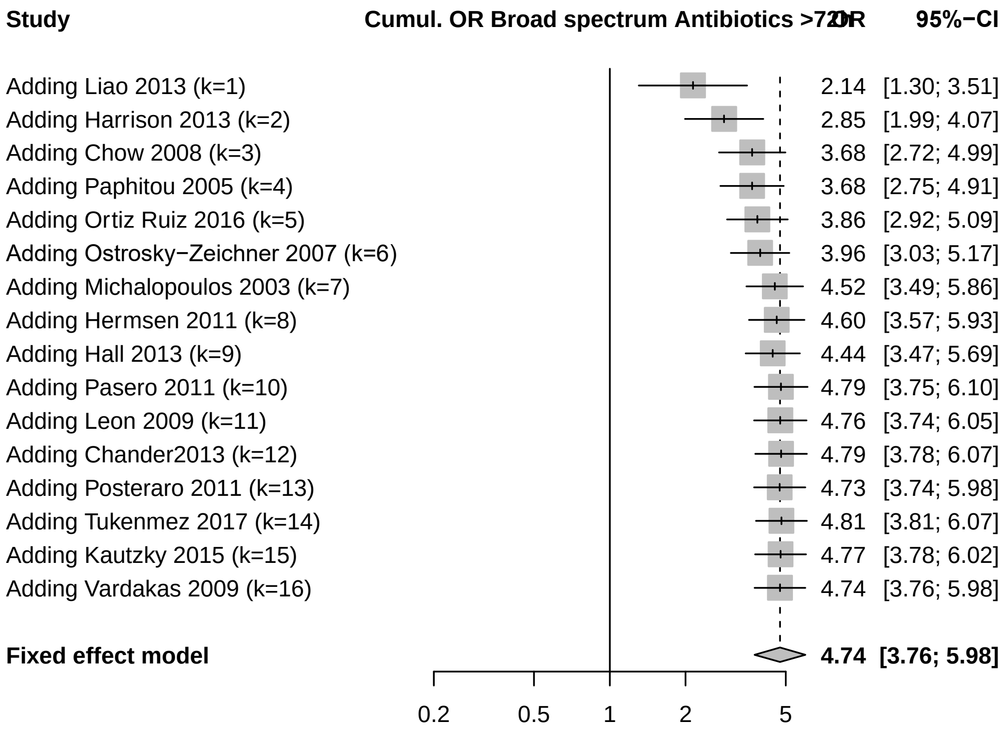
Study
Cumul. OR Broad spectrum Antibiotics >72h
OR
95%−CI
Adding Liao 2013 (k=1) 2.14 [1.30; 3.51]
Adding Harrison 2013 (k=2) 2.85 [1.99; 4.07]
Adding Chow 2008 (k=3) 3.68 [2.72; 4.99]
Adding Paphitou 2005 (k=4) 3.68 [2.75; 4.91]
Adding Ortiz Ruiz 2016 (k=5) 3.86 [2.92; 5.09]
Adding Ostrosky−Zeichner 2007 (k=6) 3.96 [3.03; 5.17]
Adding Michalopoulos 2003 (k=7) 4.52 [3.49; 5.86]
Adding Hermsen 2011 (k=8) 4.60 [3.57; 5.93]
Adding Hall 2013 (k=9) 4.44 [3.47; 5.69]
Adding Pasero 2011 (k=10) 4.79 [3.75; 6.10]
Adding Leon 2009 (k=11) 4.76 [3.74; 6.05]
Adding Chander2013 (k=12) 4.79 [3.78; 6.07]
Adding Posteraro 2011 (k=13) 4.73 [3.74; 5.98]
Adding Tukenmez 2017 (k=14) 4.81 [3.81; 6.07]
Adding Kautzky 2015 (k=15) 4.77 [3.78; 6.02]
Adding Vardakas 2009 (k=16) 4.74 [3.76; 5.98]
Fixed effect model 4.74 [3.76; 5.98]
0.2
0.5
1
2
5

e-Figure 85

Sensitivity Analysis

Cohort studies only

Plot show analysis with and without ICU length of stay as risk factor for **cohort studies only** (n = 24).

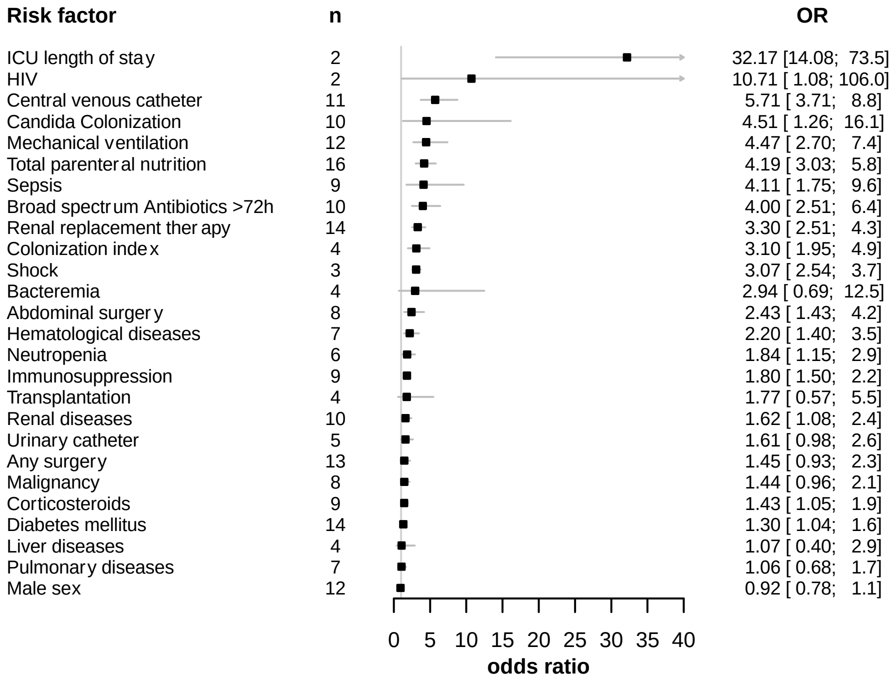

e-Figure 86: n: number of studies assessing this risk factor; or: odds ratio with 95% confidence intervals

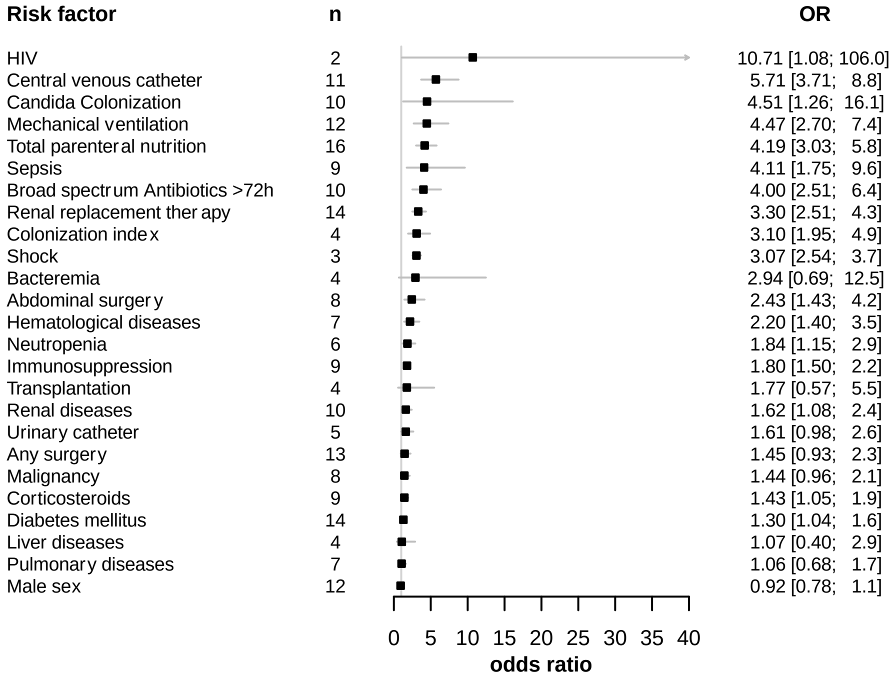

e-Figure 87: n: number of studies assessing this risk factor; or: odds ratio with 95% confidence intervals

Studies with acceptable quality only

Plot show analysis with and without ICU length of stay as risk factor with a **quality index of at least 6** (n = 23).

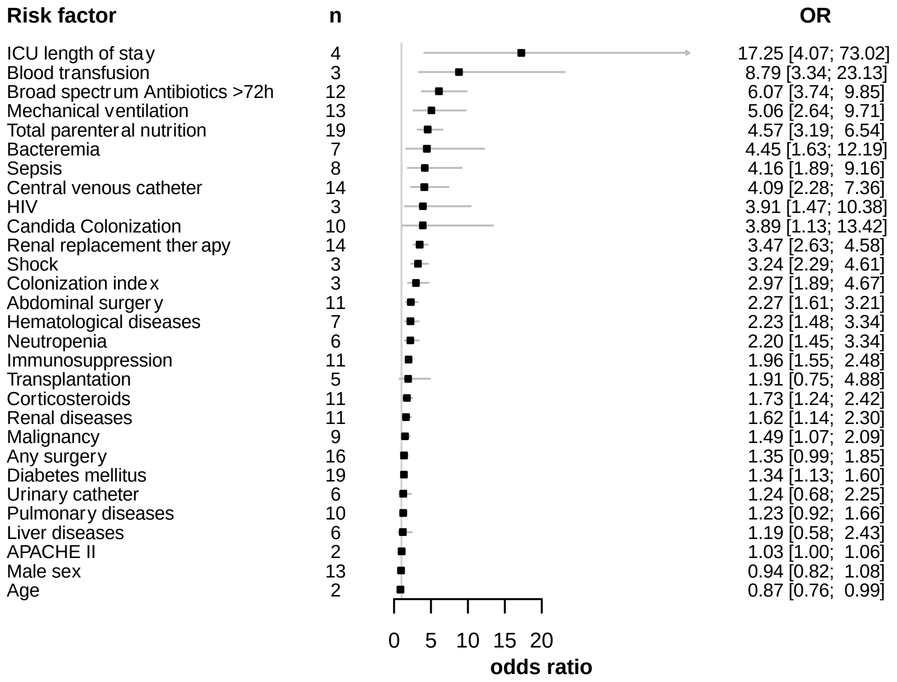

e-Figure 88: n: number of studies assessing this risk factor; or: odds ratio with 95% confidence intervals

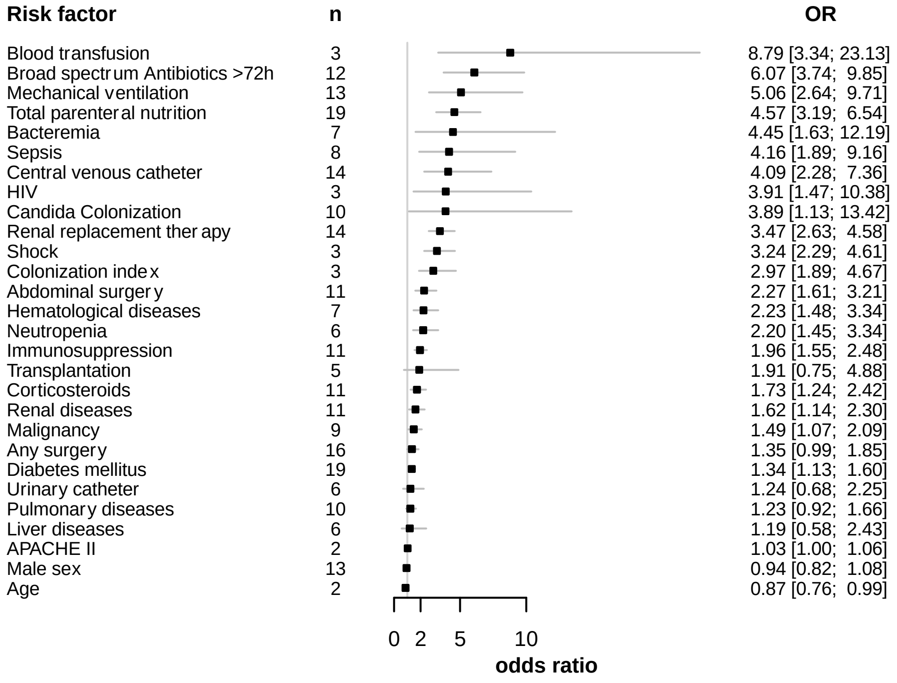

e-Figure 89: n: number of studies assessing this risk factor; or: odds ratio with 95% confidence intervals

Studies with high quality only

Plot show analysis with a **quality index of at least 9** (n = 5).

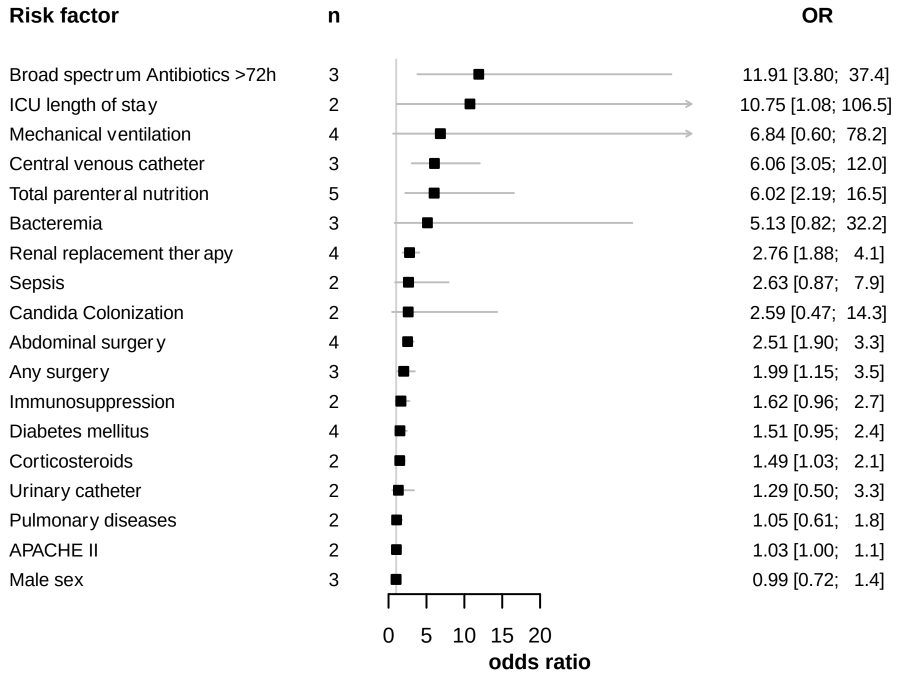

e-Figure 90: n: number of studies assessing this risk factor; or: odds ratio with 95% confidence intervals

Results adjusted data

Data were obtained from the publication as described above in the unadjusted analysis. Standard error of OR was calculated and metaanalysis was performed likewise.

Assessment of bias

This metaanalysis inludes 17 studies: 11 cohort and 6 case control studies. Overall quality was moderate with a median of 8 where 4 studies were of low, 9 studies of acceptable and 4 studies of high quality.

e-Table 5: Bias assessment for each study

Study	Design	Quality indicator
Agvald-Ohman 2008	cohort	6
Blumberg 2001	cohort	9
Burghi 2011*	cohort	2
Chow 2008	case control	8
Hall 2013	cohort	5
Han 2010	case control	7
Harrison 2013	cohort	8
Jorda-Marcos 2007	cohort	9
Kontopoulou 2014*	cohort	1
Liao 2013	cohort	8
Manolakaki 2010	cohort	5
Michalopoulos 2003	case control	9
Ortiz Ruiz 2016	case control	9
Papadimitriou-Olivgeris 2017	case control	8
Paphitou 2005	cohort	6
Pasero 2011	cohort	8
Pratikaki 2011	case control	6

*: study with abstract only

Complete analysis

The following figure shows the common odds ratios (fixed effect model) of all obtained adjusted risk factors for invasive candida infection. Risk factors represented in one study only are excluded. Forest plot for all single risk factors are shown in the appendix.

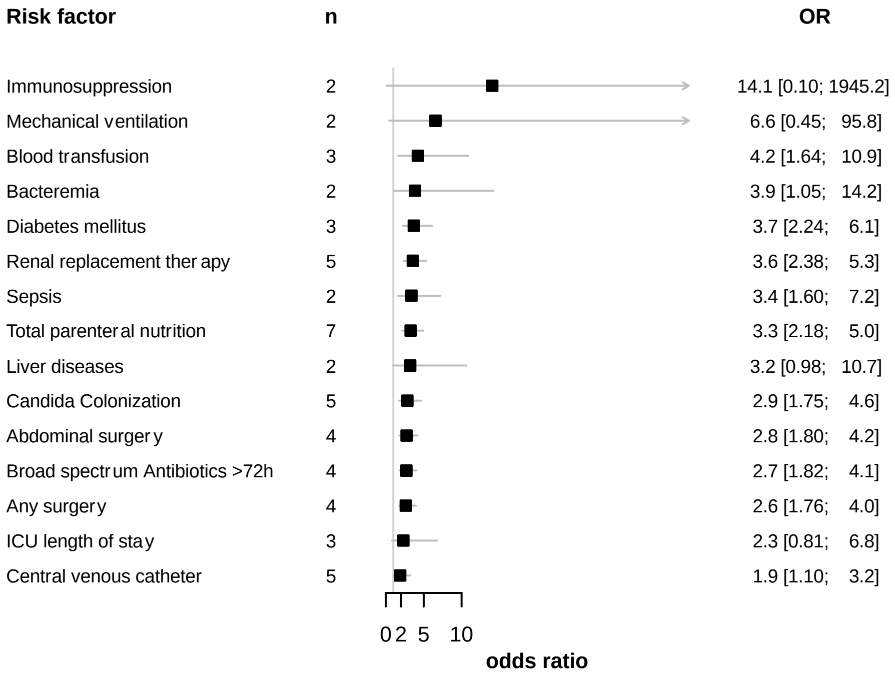

e-Figure 91: n: number of studies assessing this risk factor; or: odds ratio with 95% confidence intervals

Adjusted Meta-analyses of single risk factors

Cumulative meta-analysis is only done if two studies observed the risk factor.

Funnel plot is presented if at least seven studies reported the rsik factor and Egger test for Funnel plot asymmetry was not performed as no risk factor was reported by least ten studies.

Were only the final model after selection was available we report risk factors not selected into the final model in the footnotes. Were a model with all risk factors entered into modelling was available on request we used those, therefore there might be differences from the odds ratios reported in the original papers.

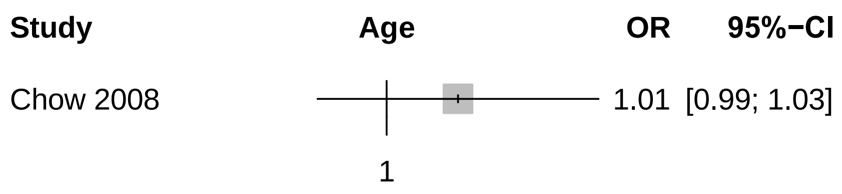

Not selected into the final model in Pratikaki 2011

e-Figure 92

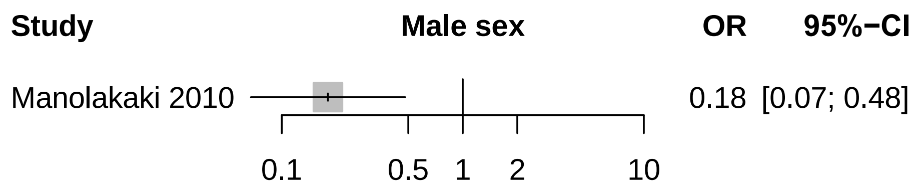

Not selected into the final model in Agvald-Ohman 2008 & Pratikaki 2011

e-Figure 93

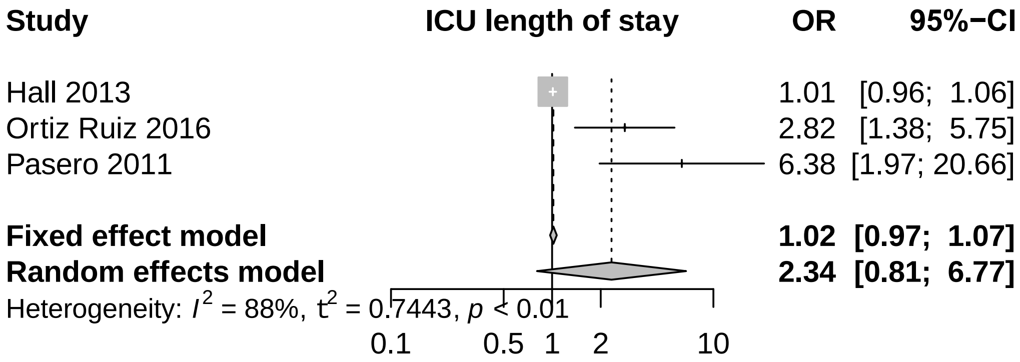

Not selected into the final model in Michalopoulos 2003

Per day of ICU LOS in Hall 2013
ICU LOS > 14 days in Ortiz Ruiz 2016
ICU LOS > 20 days in Pasero 2011

e-Figure 94

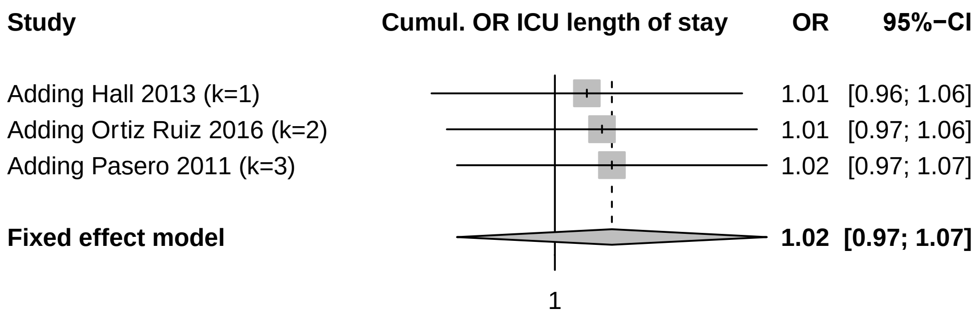
Study
Cumul. OR ICU length of stay
OR
95%−CI
Adding Hall 2013 (k=1)
1.01 [0.96; 1.06]
Adding Ortiz Ruiz 2016 (k=2)
1.01 [0.97; 1.06]
Adding Pasero 2011 (k=3)
1.02 [0.97; 1.07]
Fixed effect model
1.02 [0.97; 1.07]
1

e-Figure 95

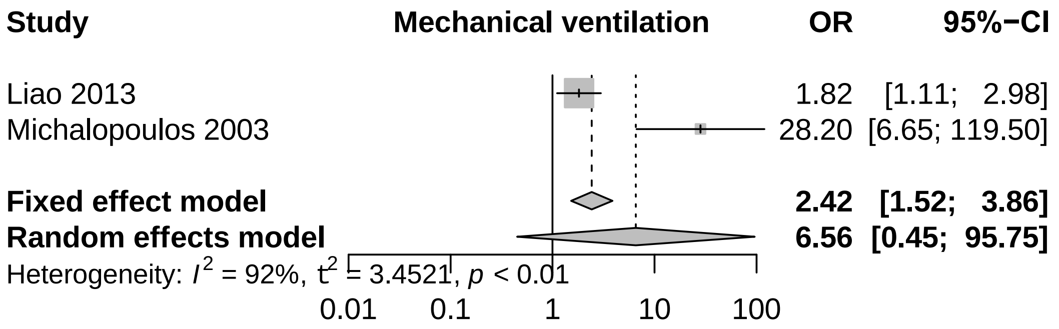

Not selected into the final model in Pasero 2011 & Pratikaki 2011

Mechanical ventilation >2 days in Liao 2013
Mechanical ventilation ≥10 days in Michalopoulos 2003

e-Figure 96

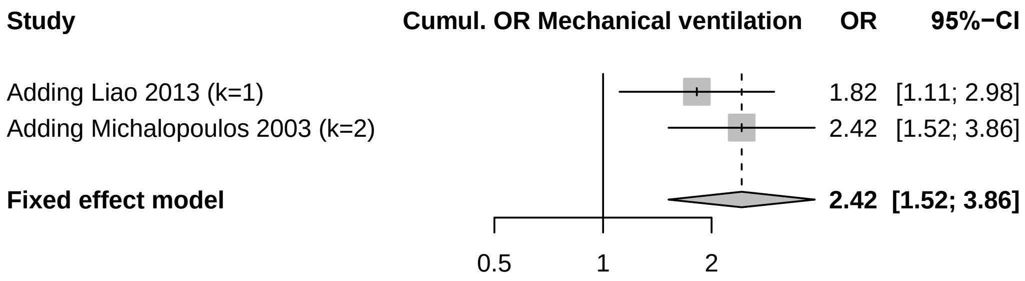
Study
Cumul. OR Mechanical ventilation
OR
95%−CI
Adding Liao 2013 (k=1)
1.82 [1.11; 2.98]
Adding Michalopoulos 2003 (k=2)
2.42 [1.52; 3.86]
Fixed effect model
2.42 [1.52; 3.86]
0.5
1
2

e-Figure 97

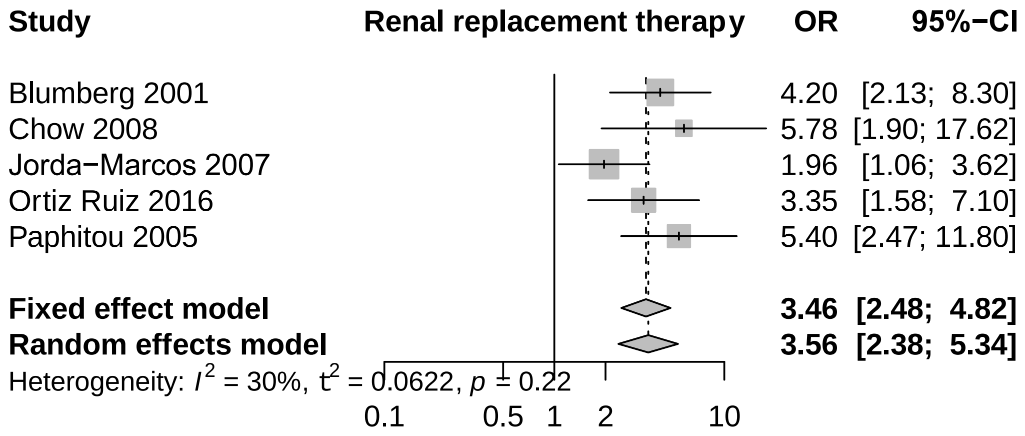

Not selected into the final model in Harrison 2013 & Pasero 2011

Hemodyalisis duration / days at risk in Chow 2008
New onset RRT day 1-3 in Papithou 2005

e-Figure 98

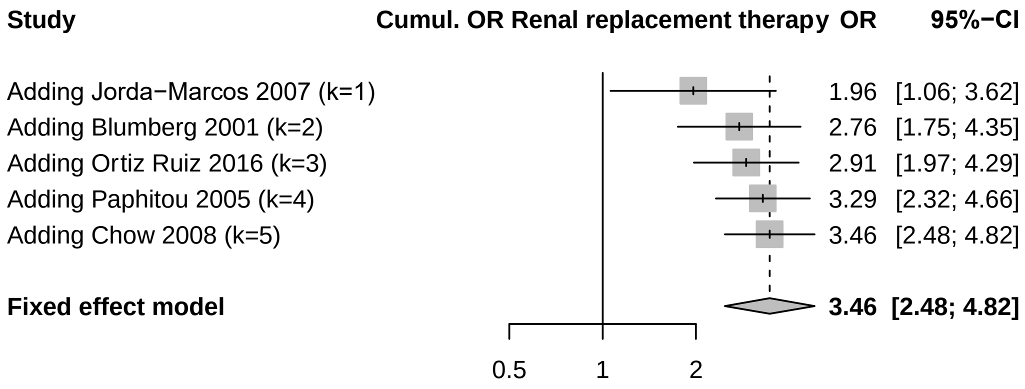
Study
Cumul. OR Renal replacement therapy
OR
95%−CI
Adding Jorda−Marcos 2007 (k=1)
1.96 [1.06; 3.62]
Adding Blumberg 2001 (k=2)
2.76 [1.75; 4.35]
Adding Ortiz Ruiz 2016 (k=3)
2.91 [1.97; 4.29]
Adding Paphitou 2005 (k=4)
3.29 [2.32; 4.66]
Adding Chow 2008 (k=5)
3.46 [2.48; 4.82]
Fixed effect model
3.46 [2.48; 4.82]
0.5
1
2

e-Figure 99

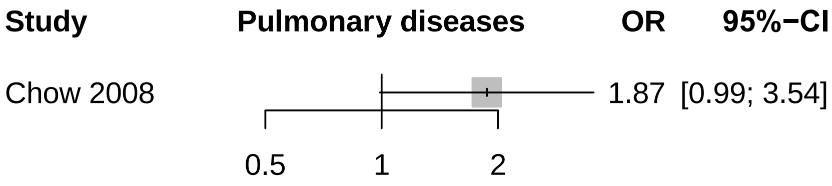

e-Figure 100

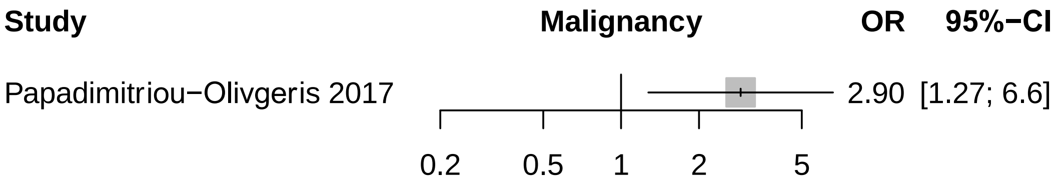

Not selected into the final model in Liao 2013 that analyzed hematological disease separately

Papadimittriou-Olivgeris 2017 assessed solid tumor or hematologic disease together

e-Figure 101

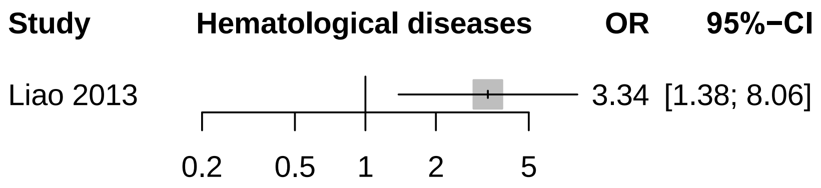
Study
Hematological diseases
OR
95%−CI
Liao 2013
3.34
[1.38; 8.06]
0.2
0.5
1
2
5

e-Figure 102

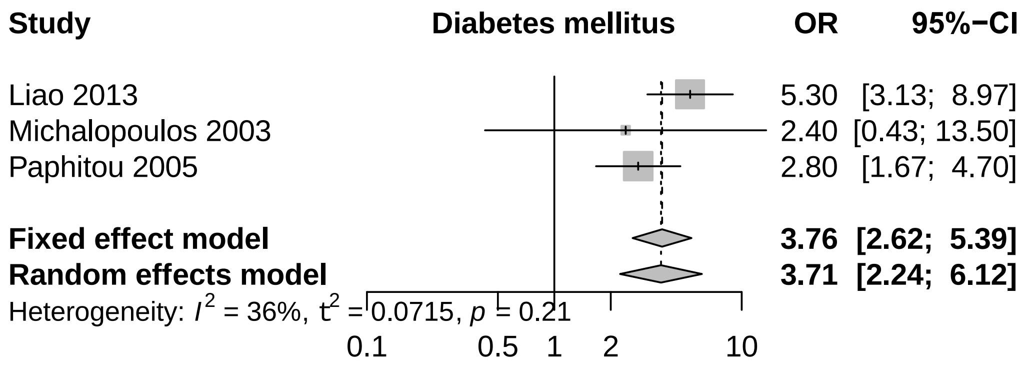

Not selected into the final model in Agvald-Ohman 2008, Pasero 2011 & Pratikaki 2011

e-Figure 103

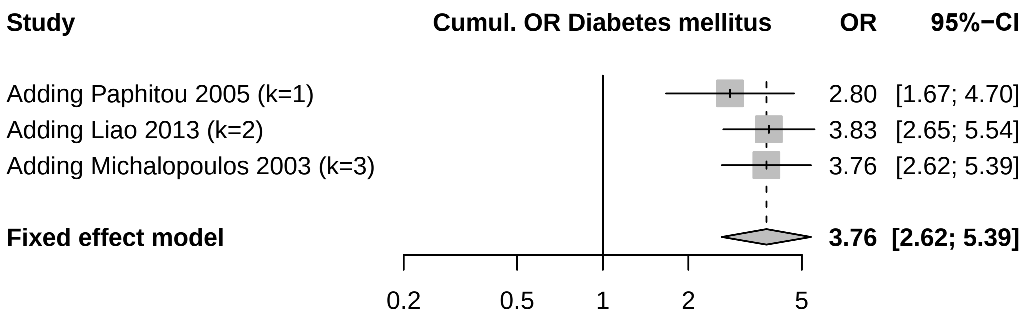
Study
Cumul. OR Diabetes mellitus
OR
95%−CI
Adding Paphitou 2005 (k=1)
2.80 [1.67; 4.70]
Adding Liao 2013 (k=2)
3.83 [2.65; 5.54]
Adding Michalopoulos 2003 (k=3)
3.76 [2.62; 5.39]
Fixed effect model
3.76 [2.62; 5.39]
0.2
0.5
1
2
5

e-Figure 104

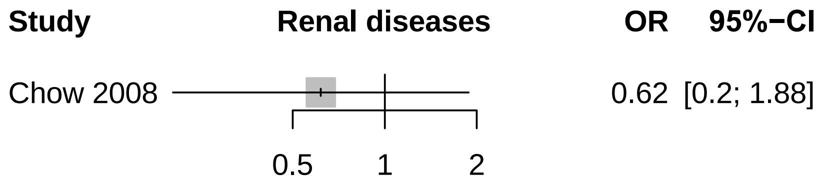

Not selected into the final model in Agvald-Ohman 2008 & Pasero 2011

Not selected into the final model in Michalopoulos 2003 & Pratikaki 2011 using the term acute renal failure

e-Figure 105

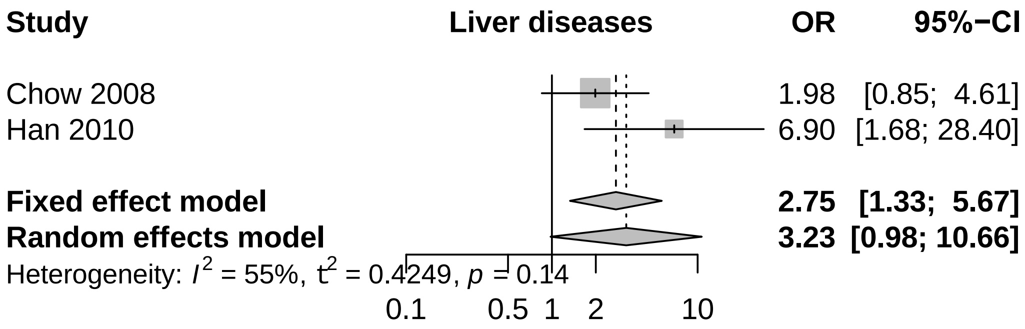

Only cirrhosis in Chowl 2009
Hepatic failure on admission in Han 2010

e-Figure 106

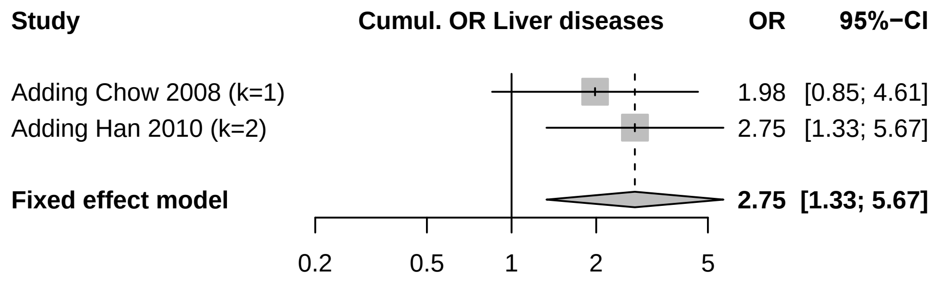
Study
Cumul. OR Liver diseases
OR
95%−CI
Adding Chow 2008 (k=1)
1.98
[0.85; 4.61]
Adding Han 2010 (k=2)
2.75
[1.33; 5.67]
Fixed effect model
2.75
[1.33; 5.67]
0.2
0.5
1
2
5

e-Figure 107

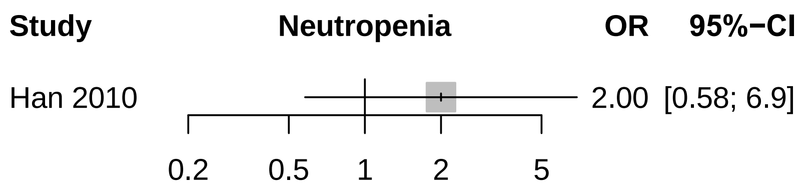

e-Figure 108

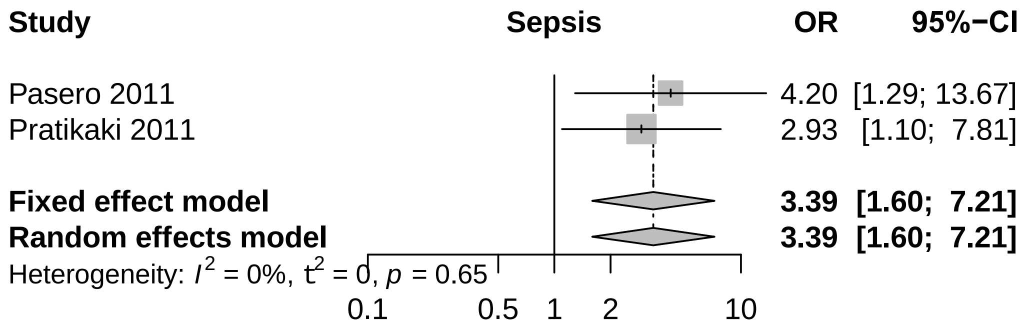

Not selected into the final model in Ortiz Ruiz 2016

e-Figure 109

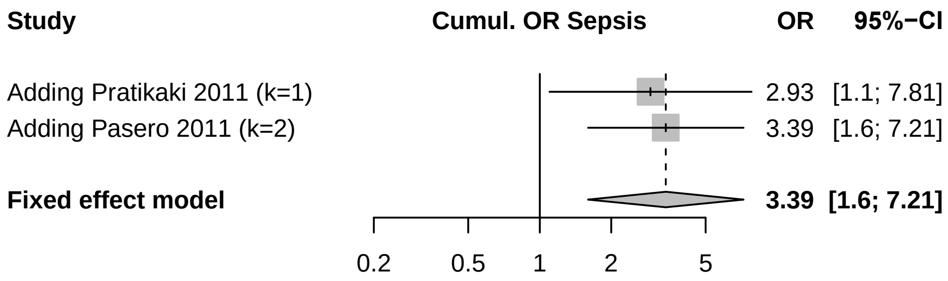

Not selected into the final model in Pasero 2011 & Pratikaki 2011

e-Figure 110

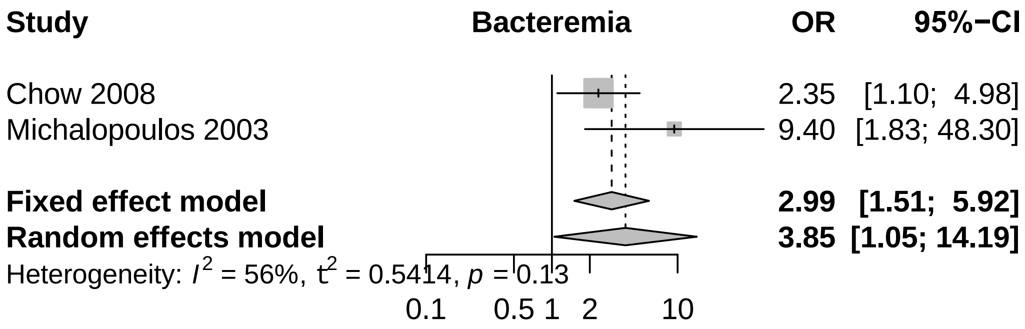
Study
Bacteremia
OR
95%−CI
Chow 2008
2.35 [1.10; 4.98]
Michalopoulos 2003
9.40 [1.83; 48.30]
Fixed effect model
2.99 [1.51; 5.92]
Random effects model
3.85 [1.05; 14.19]
Heterogeneity: I^2 = 56%, τ^2 = 0.5414, p = 0.13
0.1
0.5
1
2
10

e-Figure 111

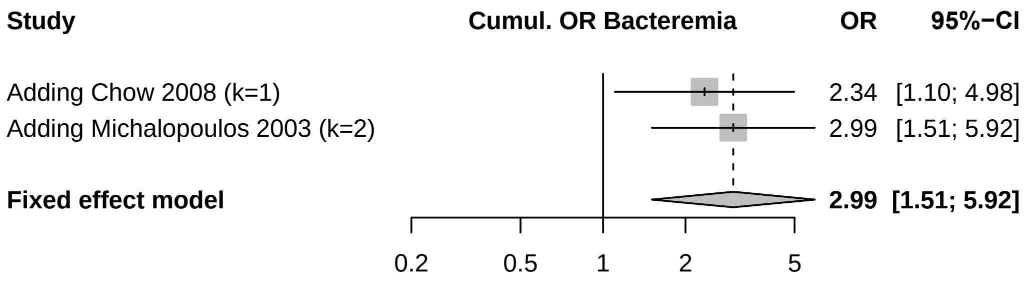
Study
Cumul. OR Bacteremia
OR
95%−CI
Adding Chow 2008 (k=1)
2.34
[1.10; 4.98]
Adding Michalopoulos 2003 (k=2)
2.99
[1.51; 5.92]
Fixed effect model
2.99
[1.51; 5.92]
0.2
0.5
1
2
5

e-Figure 112

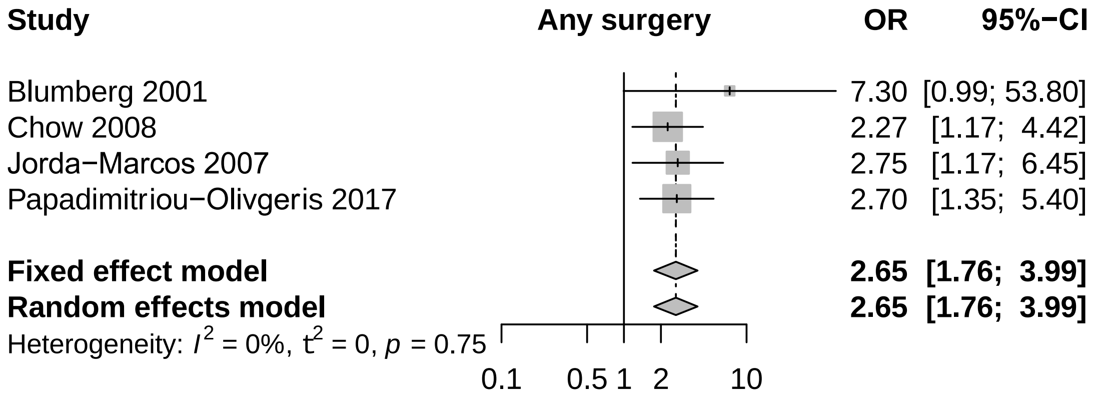

Not selected into the final model in Burghi 2011* & Harrison 2013

Elective surgery in Jorda-Marcos 2007
Prior emergency surgery in Papadimitriou-Olivgeris 2017

e-Figure 113

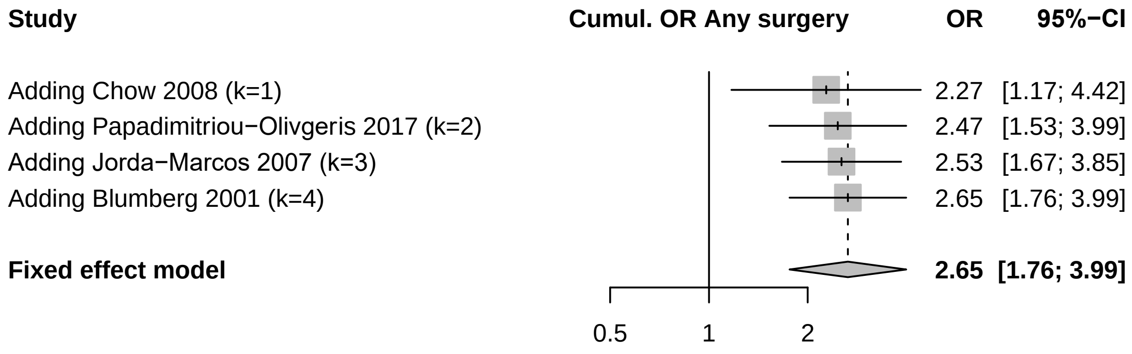
Study
Cumul. OR Any surgery
OR
95%−CI
Adding Chow 2008 (k=1)
2.27 [1.17; 4.42]
Adding Papadimitriou−Olivgeris 2017 (k=2)
2.47 [1.53; 3.99]
Adding Jorda−Marcos 2007 (k=3)
2.53 [1.67; 3.85]
Adding Blumberg 2001 (k=4)
2.65 [1.76; 3.99]
Fixed effect model
2.65 [1.76; 3.99]
0.5
1
2

e-Figure 114

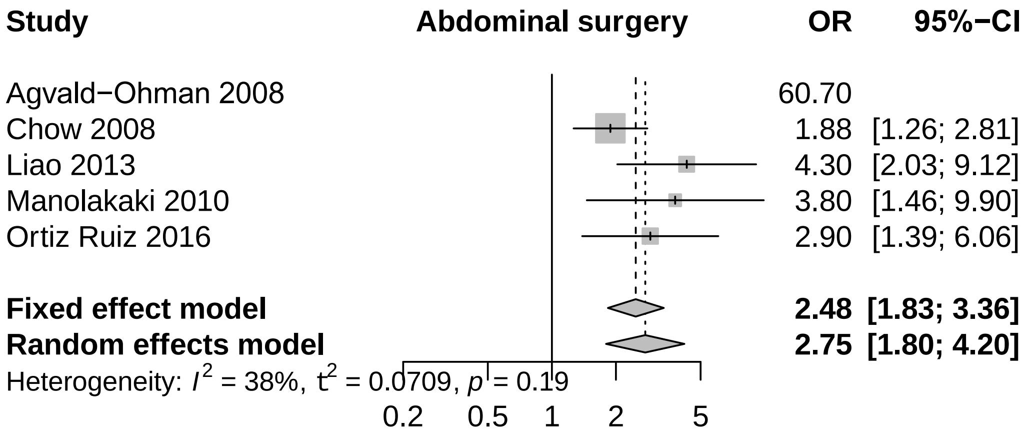

Agvald-Ohman 2008 reports an 95%-CI of 7.3 – infinity that could not be handled by the software

e-Figure 115

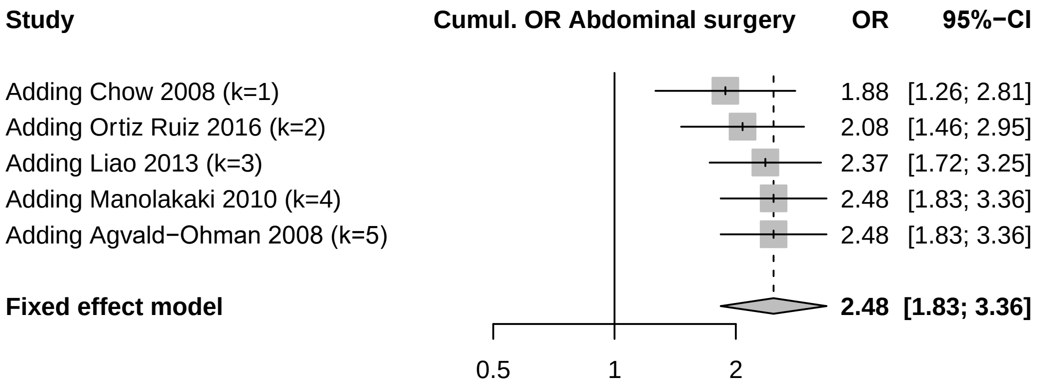
Study
Cumul. OR Abdominal surgery
OR
95%−CI
Adding Chow 2008 (k=1)
1.88
[1.26; 2.81]
Adding Ortiz Ruiz 2016 (k=2)
2.08
[1.46; 2.95]
Adding Liao 2013 (k=3)
2.37
[1.72; 3.25]
Adding Manolakaki 2010 (k=4)
2.48
[1.83; 3.36]
Adding Agvald−Ohman 2008 (k=5)
2.48
[1.83; 3.36]
Fixed effect model
2.48
[1.83; 3.36]
0.5
1
2

e-Figure 116

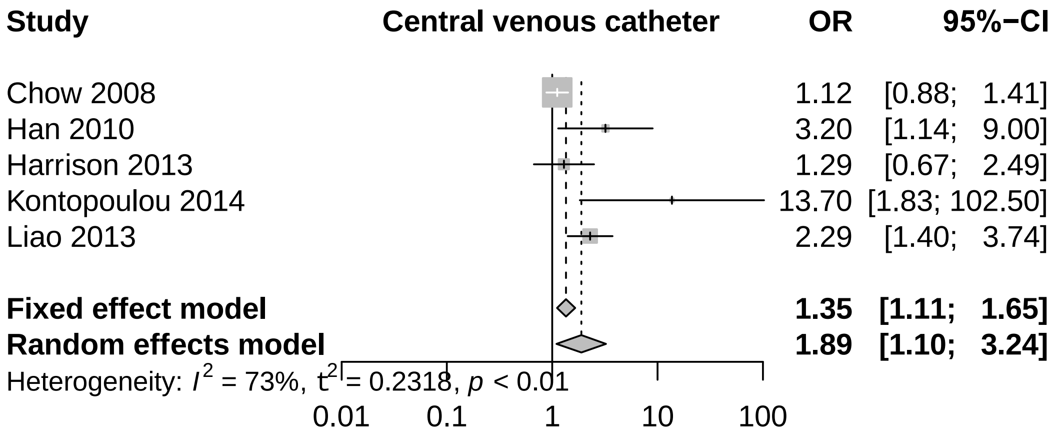

Not selected into the final model in Burghi 2011*

Not selected into the final model in Michalopoulos 2003, assessing CVC >72h

Chow 2008 calculated the OR per CVC day in their analysis

e-Figure 117

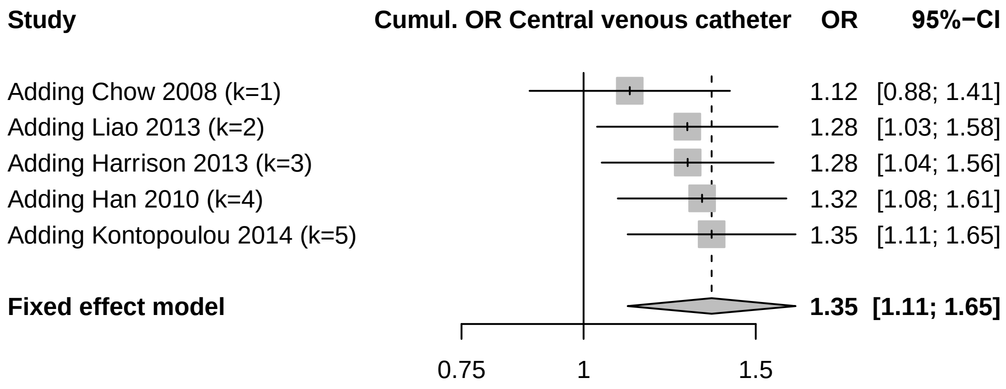
Study
Cumul. OR Central venous catheter
OR
95%−CI
Adding Chow 2008 (k=1) 1.12 [0.88; 1.41]
Adding Liao 2013 (k=2) 1.28 [1.03; 1.58]
Adding Harrison 2013 (k=3) 1.28 [1.04; 1.56]
Adding Han 2010 (k=4) 1.32 [1.08; 1.61]
Adding Kontopoulou 2014 (k=5) 1.35 [1.11; 1.65]
Fixed effect model 1.35 [1.11; 1.65]
0.75
1
1.5

e-Figure 118

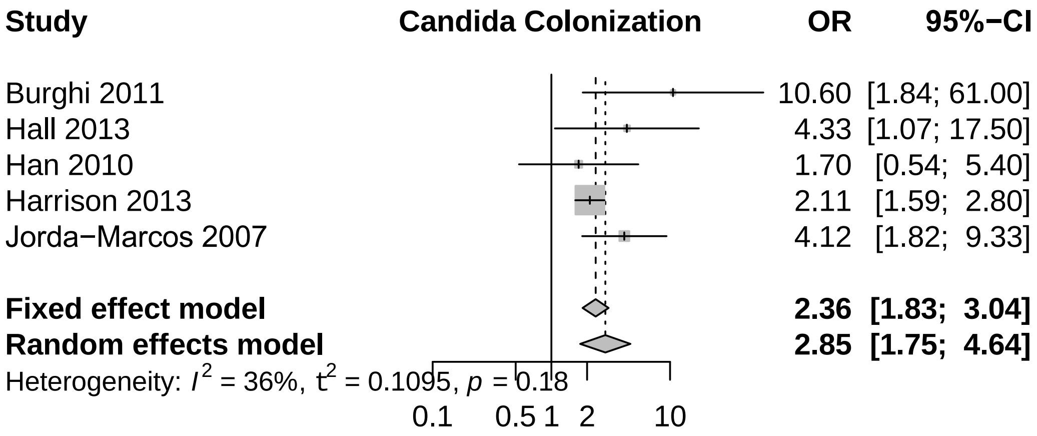

Colonization by at least two sites assessed by Burghi 2011*

e-Figure 119

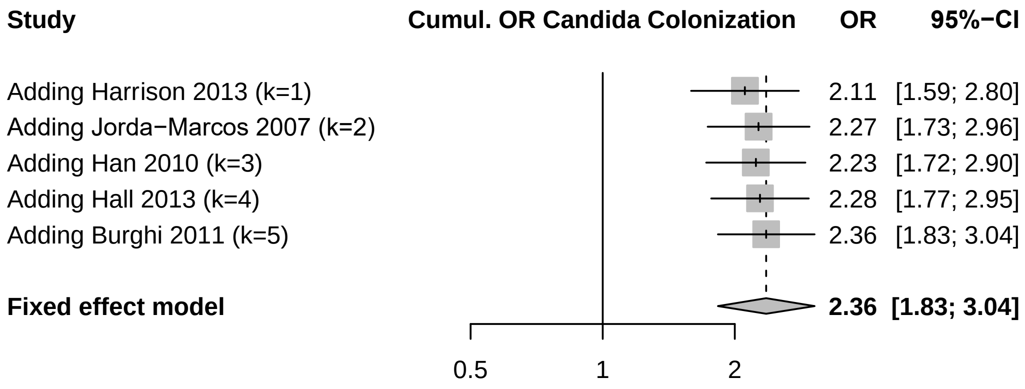
Study
Cumul. OR Candida Colonization
OR
95%−CI
Adding Harrison 2013 (k=1)
Adding Jorda−Marcos 2007 (k=2)
Adding Han 2010 (k=3)
Adding Hall 2013 (k=4)
Adding Burghi 2011 (k=5)
Fixed effect model
2.11 [1.59; 2.80]
2.27 [1.73; 2.96]
2.23 [1.72; 2.90]
2.28 [1.77; 2.95]
2.36 [1.83; 3.04]
2.36 [1.83; 3.04]
0.5
1
2

e-Figure 120

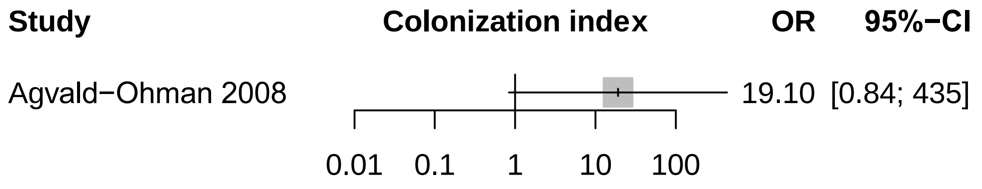

Colonization index >0.5

e-Figure 121

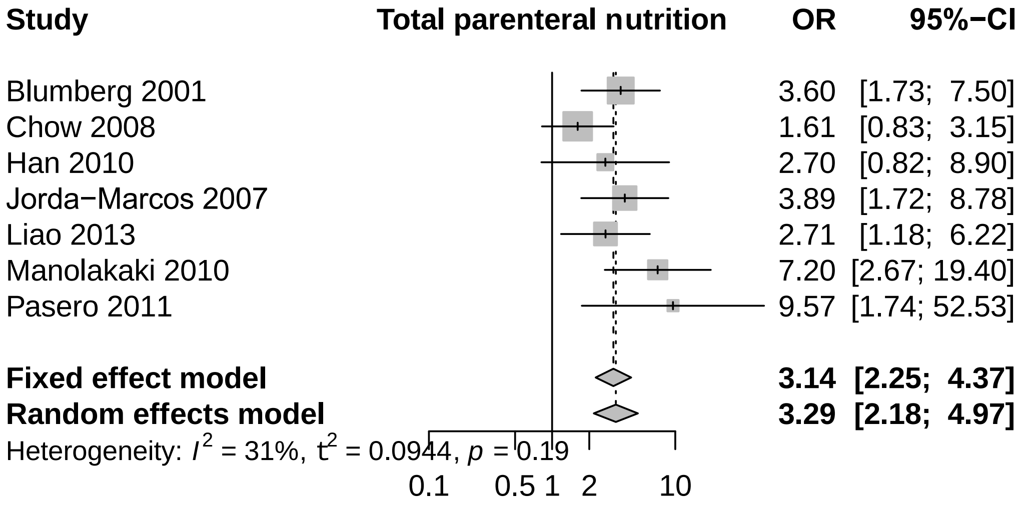

Total parenteral nutrition duration / days at risk in Chow 2008

e-Figure 122

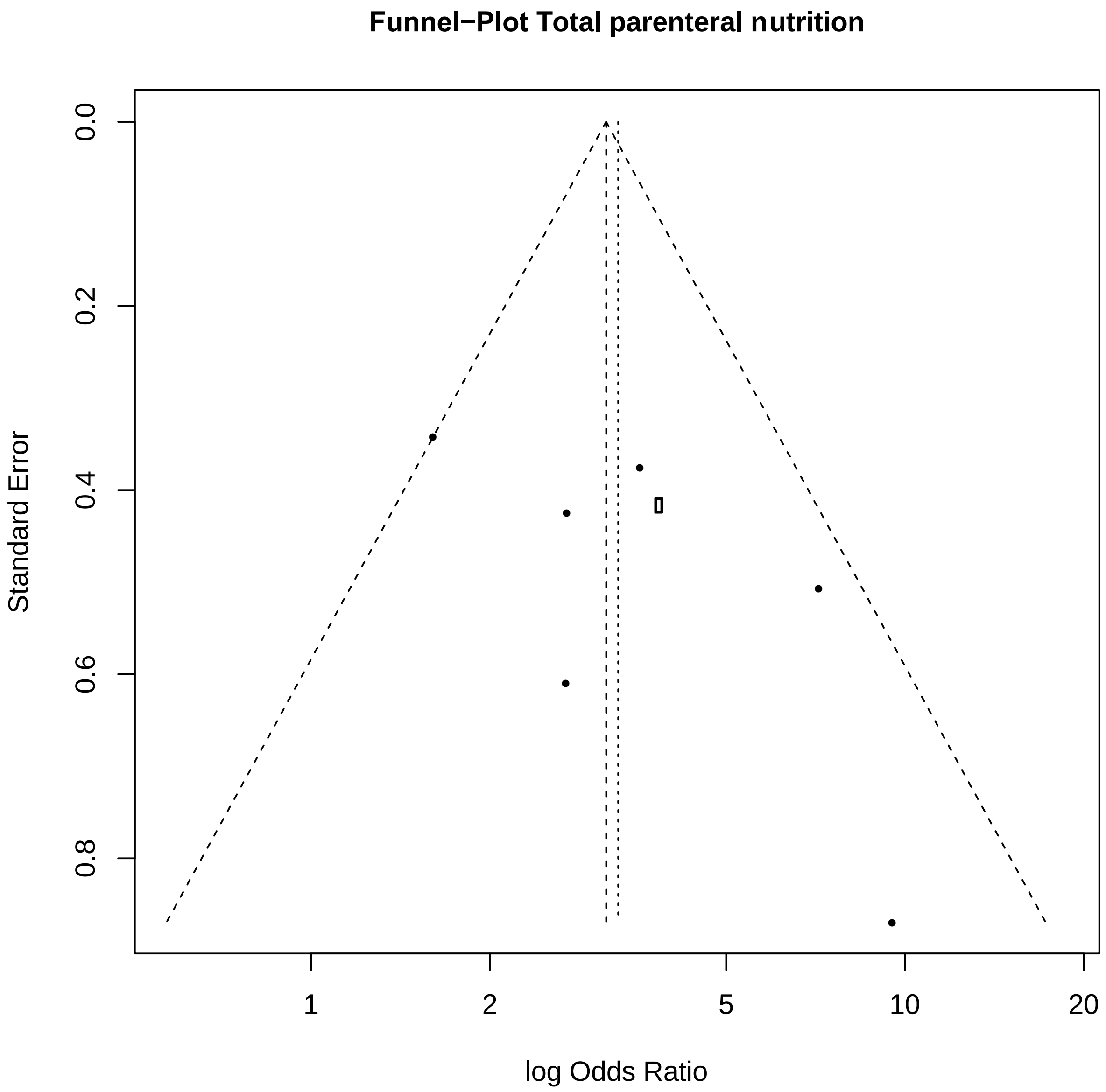

e-Figure 123

```
## [1] "Eggers test not available for less that 10 studies"
```

Study	Cumul. OR Total parenteral nutrition	OR	95%−CI
Adding Chow 2008 (k=1)		1.61	[0.83; 3.15]
Adding Blumberg 2001 (k=2)		2.32	[1.42; 3.81]
Adding Jorda−Marcos 2007 (k=3)		2.67	[1.75; 4.07]
Adding Liao 2013 (k=4)		2.68	[1.84; 3.90]
Adding Manolakaki 2010 (k=5)		3.03	[2.13; 4.31]
Adding Han 2010 (k=6)		3.00	[2.14; 4.21]
Adding Pasero 2011 (k=7)		3.14	[2.25; 4.37]
**Fixed effect model**		**3.14**	**[2.25; 4.37]**

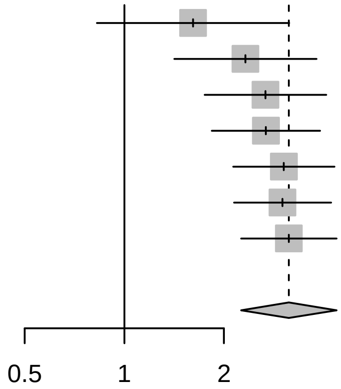

e-Figure 124

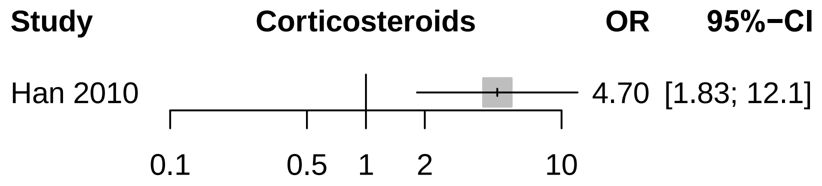

Not selected into the final model in Agvald-Ohman 2008, Burghi 2011* & Paratikaki 20011

e-Figure 125

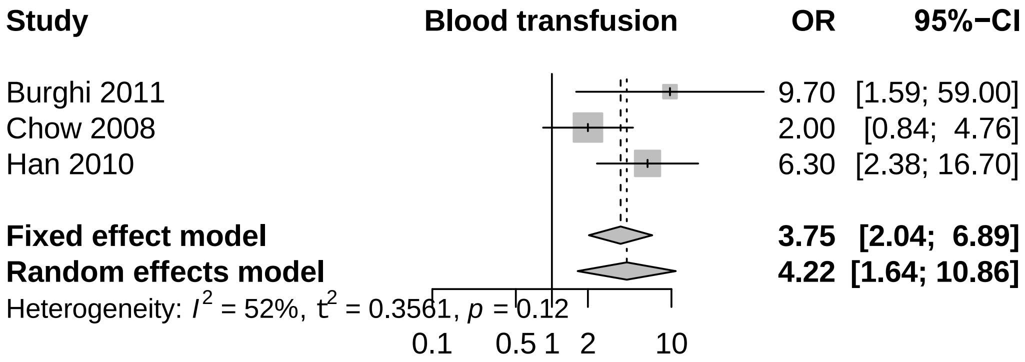

≥ 4 RBCs in Burghi 2011*
Mean RBCs/day in Chow 2008

e-Figure 126

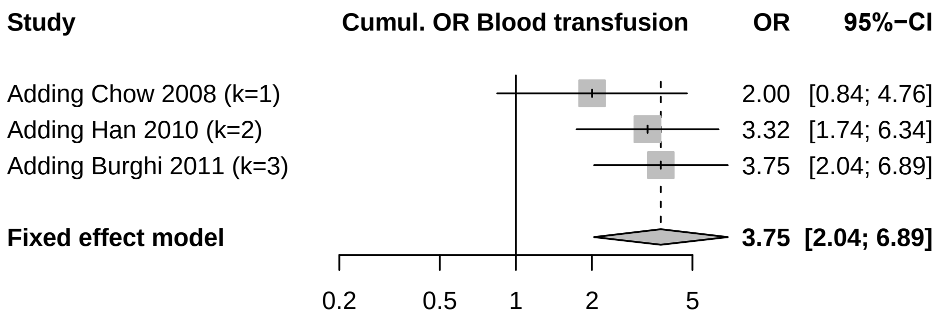
Study
Cumul. OR Blood transfusion
OR
95%−CI
Adding Chow 2008 (k=1)
2.00 [0.84; 4.76]
Adding Han 2010 (k=2)
3.32 [1.74; 6.34]
Adding Burghi 2011 (k=3)
3.75 [2.04; 6.89]
Fixed effect model
3.75 [2.04; 6.89]
0.2
0.5
1
2
5

e-Figure 127

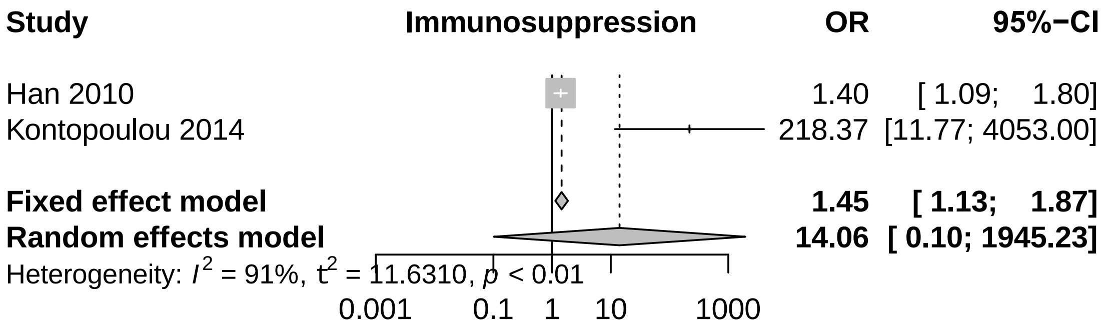
Study
Immunosuppression
OR
95%−CI
Han 2010
1.40
[1.09; 1.80]
Kontopoulou 2014
218.37
[11.77; 4053.00]
Fixed effect model
1.45
[1.13; 1.87]
Random effects model
14.06
[0.10; 1945.23]
Heterogeneity: I^2 = 91%, τ^2 = 11.6310, p < 0.01
0.001
0.1
1
10
1000

e-Figure 128

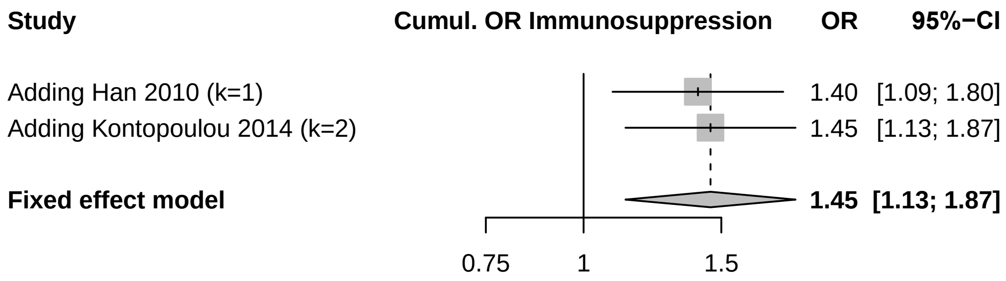

Study
Cumul. OR Immunosuppression
OR
95%−CI
Adding Han 2010 (k=1)
1.40 [1.09; 1.80]
Adding Kontopoulou 2014 (k=2)
1.45 [1.13; 1.87]
Fixed effect model
1.45 [1.13; 1.87]
0.75
1
1.5

e-Figure 129

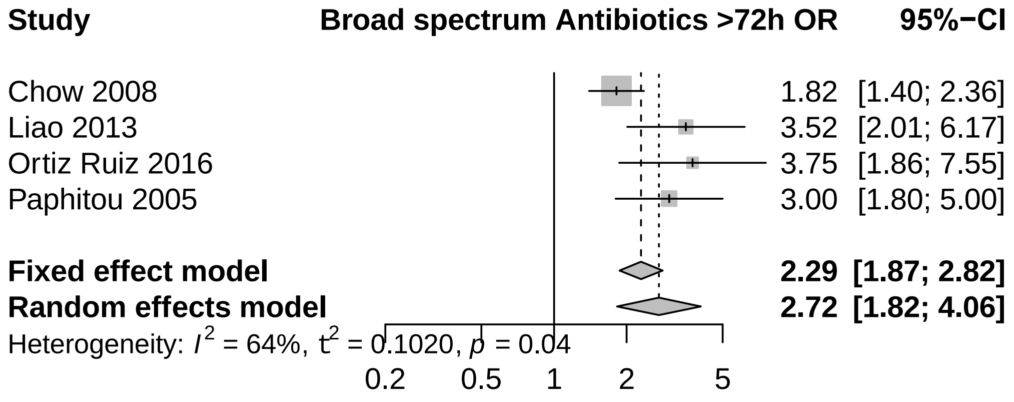

Not selected into the final model in Burghi 2011*, Michalopoulos 2003 & Pasero 2011

Antibiotic duration / days at risk in Chow 2008
> 4 days in Liao 2013
Meropenem selected into model in Ortiz Ruiz 2016

e-Figure 130

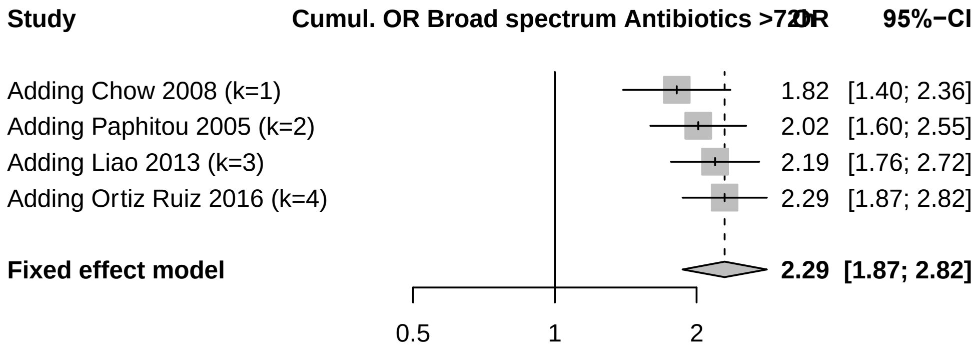
Study
Cumul. OR Broad spectrum Antibiotics >7
OR
95%−CI
Adding Chow 2008 (k=1)
1.82 [1.40; 2.36]
Adding Paphitou 2005 (k=2)
2.02 [1.60; 2.55]
Adding Liao 2013 (k=3)
2.19 [1.76; 2.72]
Adding Ortiz Ruiz 2016 (k=4)
2.29 [1.87; 2.82]
Fixed effect model
2.29 [1.87; 2.82]
0.5
1
2

e-Figure 131
